# Shared and tailored common bean transcriptomic responses to combined fusarium wilt and water deficit

**DOI:** 10.1038/s41438-021-00583-2

**Published:** 2021-07-01

**Authors:** Susana T. Leitão, Carmen Santos, Susana de Sousa Araújo, Diego Rubiales, Maria Carlota Vaz Patto

**Affiliations:** 1 Instituto de Tecnologia Química e Biológica António Xavier, Universidade Nova de Lisboa, Oeiras, Portugal; 2 Association BLC3 - Technology and Innovation Campus, Centre Bio R&D Unit, Lagares da Beira, Portugal; 3 Institute for Sustainable Agriculture, CSIC, Córdoba, Spain

**Keywords:** Biotic, Abiotic

## Abstract

Common bean (*Phaseolus vulgaris* L.), one of the most consumed food legumes worldwide, is threatened by two main constraints that are found frequently together in nature, water deficit (WD) and fusarium wilt (*Fop*). To understand the shared and unique responses of common bean to *Fop* and WD, we analyzed the transcriptomic changes and phenotypic responses in two accessions, one resistant and one susceptible to both stresses, exposed to single and combined stresses. Physiological responses (photosynthetic performance and pigments quantification) and disease progression were also assessed. The combined *Fop*WD imposition negatively affected the photosynthetic performance and increased the susceptible accession disease symptoms. The susceptible accession revealed a higher level of transcriptional changes than the resistant one, and WD single stress triggered the highest transcriptional changes. While 89 differentially expressed genes were identified exclusively in combined stresses for the susceptible accession, 35 were identified in the resistant one. These genes belong mainly to “stress”, “signaling”, “cell wall”, “hormone metabolism”, and “secondary metabolism” functional categories. Among the up-regulated genes with higher expression in the resistant accession, the cysteine-rich secretory, antigen 5 and Pr-1 (CAP) superfamily protein, a ribulose bisphosphate carboxylase family protein, and a chitinase A seem promising targets for multiple stress breeding.

## Introduction

In nature, plants are simultaneously exposed to a combination of different stresses that influence both crop growth and productivity. Global warming is increasing the frequency of heatwaves, especially in the Mediterranean area and facilitating pathogen spread, altering the habitat range of pathogens^1^. This forecasted chance to be exposed to multi-stress interactions^2^ is a challenge for plant breeding programs.

Limited data is available on plant responses in general—and on grain legumes in particular—under abiotic and biotic stress combinations hampering breeding progress. The molecular mechanisms behind plant multiple stress interactions started to be studied using mainly model species like *Arabidopsis*, *Medicago truncatula*, tomato, and tobacco^2–5^. Thus, it is essential and urgent to better understand the molecular mechanisms also used by grain legumes to improve fitness by balance growth and defense against diverse combinations of environmental constraints^6^.

The response of plants to simultaneous stress conditions is of particular interest, as one stress response pathway might interact and antagonize another, a process mainly controlled by phytohormones^4^. Besides phytohormone balance, the tradeoff between development and defense under abiotic and biotic stress conditions relies on soluble sugars, transcription factors activation, and interconnected signaling pathways, including Ca^2+^ sensing, production of reactive oxygen species (ROS) and secondary metabolites, and activation of protein kinase cascades^5,7–9^. Furthermore, unique responses occur in plants simultaneously exposed to two or more stresses. These responses—called tailored—are different from the responses that occur in plants exposed to the same individual stresses^10^. In the particular case of a biotic-abiotic stress combination, several studies demonstrated that a preceding abiotic stress may induce pathogen resistance responses in plants^11,12^. For instance, the generation of ROS and the accumulation of abscisic acid (ABA) caused by moderate drought stress was reported to enhance plant defense against pathogens, by inducing the expression of defense-related genes^13^.

However, it has been also claimed that many abiotic stress conditions, such as water deficit (WD) and heat, weaken the defense mechanisms of plants and enhanced their susceptibility to both aerial and root pathogen infection^14,15^. Likewise, pathogen infection has been shown to reduce photosynthesis and water use efficiency^16^ and induce abnormal stomata opening patterns^17^, with consequences in plant tolerance to abiotic stress. Indeed, and despite some existing overlap, each stress condition induces a unique mechanism of response and each combination of two or more different stresses triggers a more complex plant response than the individual stresses^18^.

Common bean (*Phaseolus vulgaris* L.) is one of the most important grain legumes for human consumption in the world, with over 36 Mha cultivated in 2018 (FAOSTAT). This crop is threatened by a series of biotic and abiotic constraints during life cycle, severely limiting its yield. Diseases and pests are frequently important factors compromising common bean production^19^. Among these, Fusarium wilt, caused by the soilborne fungus *Fusarium oxysporum* f. sp. *phaseoli* (*Fop*), has been detected in most of the bean-growing regions of the world, causing significant yield losses^20,21^. Also, drought stress affects over 60% of the common bean production worldwide^22^. The wilt fungi are known to interfere with the water relations of plants by colonizing the xylem vessels where they proliferate and obstruct the transportation of water and nutrients^23^. Additionally, the fungal species distribution is strongly associated with the ability of pathogenic strains to survive periods of drought^24^. However, few studies exist on the phenotypic interaction of abiotic and biotic stresses in common bean. One of them characterized phenotypically the simultaneous exposure of four common bean cultivars to drought stress and a fungal pathogen, *Macrophomina phaseolina* (causal agent of charcoal rot)^25^. The combined stresses resulted in a higher transpiration rate and leaf temperature as compared to plants only subjected to drought stress. Other described, on the other hand, the negative effect of combined drought stress and *Ophiomyia* spp. (bean fly) infestation on the mean seed yield and leaf chlorophyll content of two diverse common bean recombinant inbred line populations^26^. Moreover, the transcriptional profile linked to concurrent stresses adaptation in common bean is also still poorly understood as well as the central hub genes and key pathways controlling abiotic and biotic stress interaction. As a consequence, it is difficult to predict core stress-signature pathways overlapping single and combined stresses that could be relevant for the development of multi-stress resistant plants.

The present work was designed to identify common bean transcriptional changes in response to single and combined water-deficit and fusarium wilt stresses. The identification of molecular signatures common to different stresses will be relevant for the development of multi-stress resistant varieties.

To attain this objective, we focused on two Portuguese common bean accessions, with contrasting responses to both WD and *Fop*^27,28^. The two accessions were exposed to single and combined stresses and the root transcriptome profiling was compared using massive analysis of cDNA ends (MACE). Roots were chosen since both WD and fusarium wilt interact primarily with this tissue. The identification of common bean differentially expressed genes (DEGs) and key pathways activated against *Fop* and WD interaction will contribute to a better understanding of the combined common bean responses to these two stresses frequently concurrent in nature.

## Results

Portuguese common bean accessions R-645 and S-1955, previously reported as resistant and susceptible, respectively against both fusarium wilt (*Fop*) and early water deficit (WD) were evaluated under single stresses (*Fop* or *WD*), and combined fusarium wilt and water deficit (*Fop*WD) stresses. Using plants at early vegetative growth stage (V1 to V2), the following photosynthesis-related traits were evaluated at three time points (T1: 48 h, T2: 96 h, and T3: 8 days after stress imposition): stomatal CO_2_ conductance (gs), net CO_2_ assimilation rate (A), transpiration rate (E), instantaneous and intrinsic water-use efficiencies (WUE = A/E and WUEi = A/gs, respectively), chlorophylls a (C*a*) and b (C*b*) contents, carotene and xanthophyll (C*cx*) contents. Leaf relative water content (RWC) was also evaluated, as well as disease visual symptoms caused by inoculation with *Fusarium oxysporum* f. sp. *phaseoli* isolate FOP-SP1 race 6 (*Fop*). Roots collected at T2 (96 h after stress imposition) from both accessions under control (non-inoculation and well-watered), single, and combined stresses, were used for transcriptome analysis using RNA-seq variant MACE – massive analysis of cDNA ends.

### Phenotypic traits variation

At T1 (48 h after stress imposition) and T2 (96 h after stress imposition), no visible symptoms were observed in the plants from both susceptible (S-1955) and resistant (R-645) accessions, under single *Fop*, WD, and combined *Fop*WD stresses. Eight days after stress imposition (T3), only the plants from the susceptible accession showed wilting and chlorotic symptoms in the leaves under *Fop* single stress, with additional signs of necrosis under *Fop*WD combined stresses. Additionally, at T3, and under single WD, plants from the susceptible accession presented wilting symptoms that were not observed in the plants from the resistant accession. Since the roots of all plants were removed at the defined time points, three extra plants of each accession were inoculated to verify the full development of the disease in the susceptible accession. As expected from our previous work^27^, accession S-1955 was susceptible to *Fop* and the three extra plants were dead 20 days after inoculation, while the plants from accession R-645 only presented minor symptoms (slight yellowing of leaves margins) that did not aggravate.

The REML variance components analysis showed that there were significant differences between accessions in C*a*, C*b*, C*cx*, A, A/gs, and A/E (Supplementary Table S1). For all these traits, the resistant accession had a higher mean value compared to the susceptible accession. Significant differences were also detected among treatments for all the photosynthetic pigments contents and ratios and for the gas exchange parameters A, E, gs, and A/E. In particular, the most significant decreases in relation to the control conditions were found for A under the combined stress and for the resistant accession, and for E and gs under *Fop* single stress and under combined stresses, in both accessions. The reduction of E under combined stresses was significantly higher than the observed under *Fop* single stress only for the susceptible accession. In this accession, early water deficit also caused a significant reduction of gs in comparison to the control conditions. The photosynthetic pigments most significant changes in relation to the control conditions were observed for C*b* that increased under combined stresses in both accessions, and in the susceptible accession also under *Fop* single stress. C*a* and C*cx* decreased under water deficit single stress in the susceptible accession. Despite the decrease in soil water content and physiological differences observed, no significant variation on leaf RWC was detected between accessions or among treatments (Supplementary Table S1).

When analyzing the progression over time, an increase in the photosynthetic pigment contents was observed from T1 (48 h) to T2 (96 h), except for C*b* that maintained similar values, followed by a decrease from T2 to T3 (8 days) in all the three pigment contents. Moreover, T2 was the time point that phenotypically differentiated the most the different accessions and treatments (Supplementary Fig. S1 and Supplementary Table S1).

Indeed, the most contrasting responses (for A, E, gs, C*a*, C*b* and C*cx*) between accessions and treatments were observed at T2 (Supplementary Fig. S1 and Supplementary Tables S1–S3). At this time point, the two accessions behaved very differently under single early WD, with the A value in the resistant accession being 8 times higher than in the susceptible accession (Supplementary Table S2). Also, at T2 and in relation to control conditions, gs and E were significantly reduced under all the treatments in the susceptible accession (Supplementary Table S3). The same was not observed for the resistant accession (Supplementary Table S3). Still at T2 and for both accessions, C*a*, C*b*, C*cx*, and C*a* + C*b* values under the three treatments were not significantly different from the control samples.

Eight days (T3) after stress imposition, the differences among treatments within accessions decreased, and only the C*b* values of the resistant accession under *Fop*WD were significantly different (higher) from the control samples (Supplementary Table S3).

**Fig. 1:**
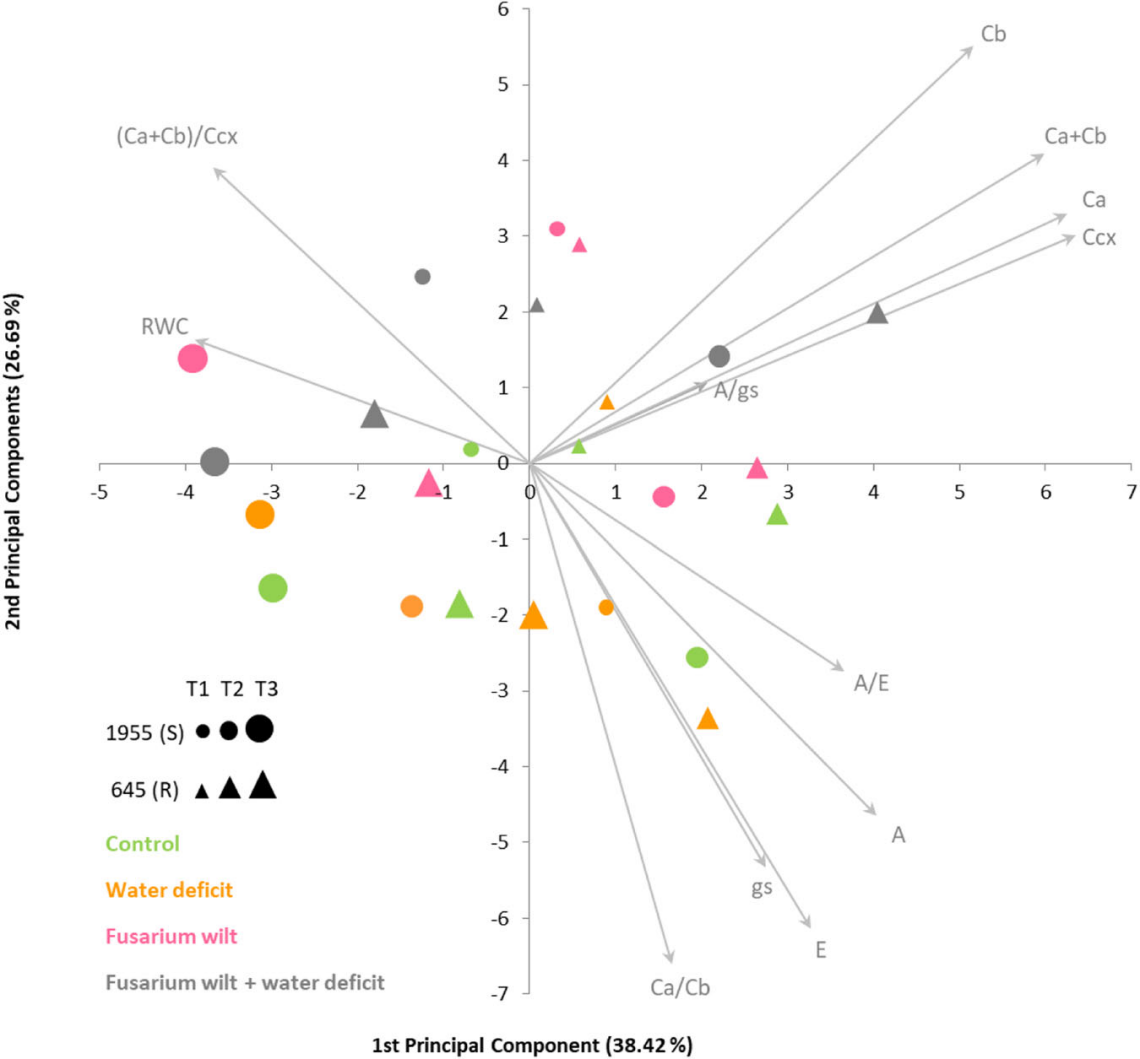
Principal components analysis based on the photosynthetic parameters average values and leaf relative water content, for each accession, treatment, and time point. The resistant accession (R-645) is represented by triangles and the susceptible accession (S-1955) by circles. Treatments: control in green, water deficit in orange, fusarium wilt in pink, fusarium wilt + water deficit in grey. Time points: T1 = 48 h after stress imposition, smaller symbols; T2 = 96 h after stress imposition, intermediate symbols; and T3 = 8 days after stress imposition, larger symbols. The first two principal components explained 65.11% of the variance observed

A principal component analysis was performed to assess the relationship among the two contrasting common bean accessions stress responses and to visualize if the different treatments and time points clustered separately, based on the results from the 12 traits measured (Fig. 1). All traits contributed with a similar weight, except for intrinsic water-use efficiency (A/gs) with a smaller vector. The first principal component, explaining 38.42% of the total variation observed, separated the majority of the samples by time point (T1 with an intermediate position between T3 and T2). The exception was the susceptible accession at T2 under WD (single stress) that was displayed closer to the accessions at T3. The second principal component, explaining 26.69% of the total variance observed, mostly separated the samples under control and WD single stress from the samples under *Fop* single stress and *Fop*WD combined stress. From the biplot, we inferred that in general samples at T3 had the smallest photosynthetic pigment concentrations and A/gs, samples from *Fop* and *Fop*WD treatments had the smallest A, E, gs, and A/E, and those parameters were more differentiated among treatments at T2. This led us to choose T2 (96 h) as the time point to perform the transcriptomics analysis.

### MACE libraries characterization

Twenty-four MACE libraries were constructed to outline transcriptomic changes in roots collected 96 h (T2) after stress imposition. These corresponded to two accessions (R-645 resistant and S-1955 susceptible to both stresses), four treatments (Ctrl, WD, *Fop*, and *Fop*WD), and three biological replicates for each condition. The number of raw reads per condition ranged from 11,601,286 (sample R-645 Ctrl) to 20,749,104 (sample R-645 WD) (Table 1).

**Table 1:** List of raw, cleaned, and map read counts obtained for each library after Massive Analysis of cDNA Ends (MACE)

Sample/MACE library	Raw reads	Cleaned reads	Mapped reads	% Mapped reads
R Ctrl	11,601,286	6,336,407	6,264,779	98.87
R WD	20,749,104	11,150,960	11,024,583	98.87
R *Fop*	13,001,084	7,193,821	7,112,113	98.86
R *Fop*WD	12,847,265	6,899,819	6,821,410	98.86
S Ctrl	16,234,892	8,506,887	8,411,388	98.88
S WD	12,954,307	6,305,977	6,236,012	98.89
S *Fop*	12,533,221	6,419,584	6,346,990	98.87
S *Fop*WD	12,846,063	6,465,866	6,392,371	98.86

The percentage of mapped reads was calculated using the number of mapped reads/cleaned reads. *R* resistant accession (R-645), *S* susceptible accession (S-1955), *Ctrl* control, *WD* water deficit single stress, *Fop* fusarium wilt single stress, *Fop*WD combined fusarium wilt + water deficit stresses. The values are averages of the three root biological replicates per condition

Phvul.003G109300—MLP423 MLP-like protein 423 (144,602 counts in average), Phvul.002G207400 - bifunctional inhibitor/lipid-transfer protein/seed storage 2 S albumin superfamily protein (71,536 counts in average), and Phvul.007G099700—RSH extensin 3 (48,212 counts in average) were amongst the genes with highest total raw read counts, suggesting no ribosomal RNA (rRNA) contamination during library preparation.

### Differentially expressed genes (DEGs) between accessions and treatments

A total of 22,553 genes were expressed and identified at T2 among all the root samples analyzed in this study. Of those, 1,171 genes were considered DEGs for each stress condition (WD, *Fop*, *Fop*WD) compared with control (Ctrl), based on the imposed thresholds (Supplementary File S1).

**Fig. 2:**
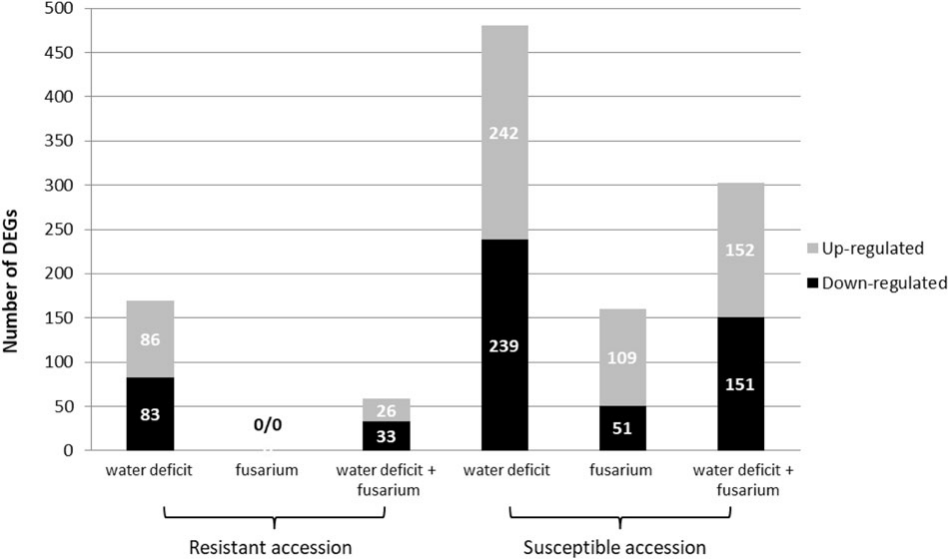
Differentially expressed genes between accessions and treatments. The up and down-regulated genes were identified in roots 96 h after stress imposition in comparison with the control condition in a double resistant (R-645) and a double susceptible (S-1955) common bean accessions. The thresholds applied were *P*-value ≤ 0.05, FDR ≤ 0.01, |log_2_FC| ≥ 1.5

In response to the applied treatments, a higher level of root transcriptional changes was detected in the susceptible accession. While 944 DEGs were found in the susceptible accession, only 228 DEGs were found in the resistant accession. In the susceptible accession, early WD was the treatment with more DEGs identified (242 up-regulated and 239 down-regulated genes), followed by *Fop*WD combined stress (152 up-regulated and 151 down-regulated) (Fig. 2). Similarly, in the resistant accession, WD was also the treatment with more DEGs identified (86 up-regulated and 83 down-regulated) followed by the *Fop*WD combined stress where 26 up-regulated and 33 down-regulated genes were identified. However, in the resistant accession, no DEGs were observed under fusarium wilt single stress at T2. In general, the number of genes up-regulated and down-regulated in each condition was similar. The exception was the condition *Fop* for the susceptible accession in which the number of up-regulated genes was twice the number of down-regulated genes (109 vs 51) (Fig. 2).

A PCA to assess the relative distribution of all biological replicates of each experimental condition was performed (Supplementary Fig. S2) using as dataset the raw read counts of 294 randomly selected DEGs, instead of the total 1,171 DEGs, due to statistical software limitations. The first two principal components (PCs) explained 35 and 23% of the variance, respectively. In general, the three biological replicates per accession/condition clustered reasonably together. However, PC1 separated three replicates of the resistant accession, one at each condition, from the remaining replicates (Supplementary Fig. S2).

#### Functional categorization of DEGs

**Fig. 3:**
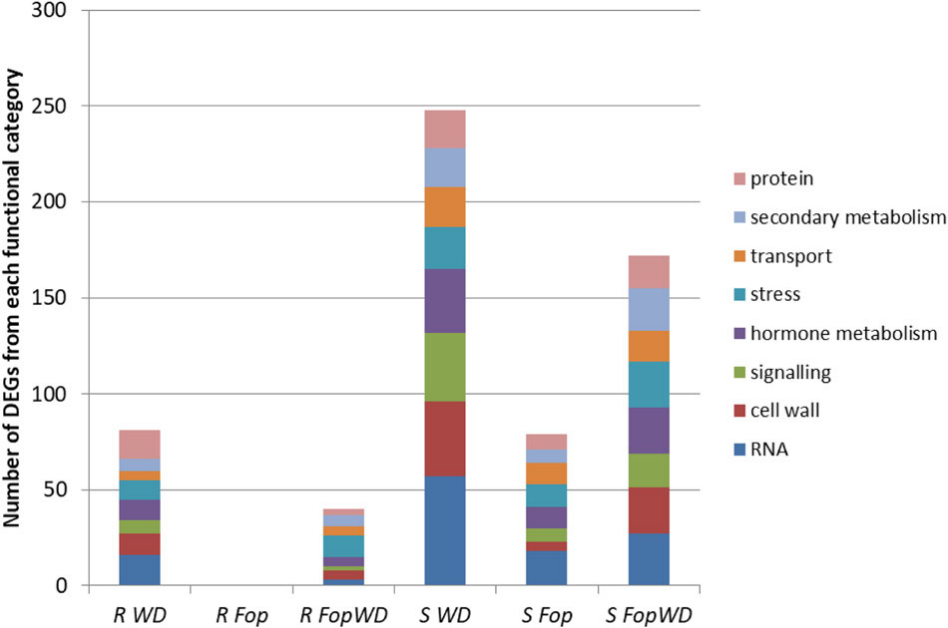
Number of DEGs in the most represented functional categories, assigned by MapMan, expressed in the roots of the resistant and susceptible accessions in the different conditions. R = resistant accession (R-645), S = susceptible accession (S-1955), WD = water deficit single stress, *Fop* = fusarium wilt single stress, *Fop*WD = combined fusarium wilt + water deficit stress

Functional categories were assigned to the identified root DEGs using the MapMan web tools (Supplementary Table S4 and Supplementary Fig. S3). The most represented categories in all the conditions were “miscellaneous”, “RNA”, “secondary metabolism”, “stress”, “signaling”, “hormone metabolism”, transport”, “protein”, and “cell wall” (Fig. 3). In general, the same categories were found in up- and down-regulated genes. The functional category “not assigned” accounted for 27.23% of the DEGs.

A higher percentage of DEGs from the functional category “stress” (15.28%) was identified for the combined stress situation in the roots of the resistant accession, in comparison with the other conditions where the “stress”-related DEGs ranged from 4.12 to 7.23% (Supplementary Fig. S4).

Some functional categories less represented were only assigned to the susceptible accession, namely, “amino acid metabolism” and “redox”, categorizing DEGs under WD and *Fop*WD, and “cell” under all treatments. Moreover, some functional categories were only assigned to DEGs exclusively up or down-regulated. For instance, “minor CHO metabolism” was only assigned to up-regulated genes in all the treatments for both accessions. On the other side, “OPP.non-reductive PP.transaldolase” was only assigned to down-regulated genes, in *Fop*WD for the resistant accession, and in WD and *Fop*WD for the susceptible accession.

One hundred ninety-eight genes were simultaneously expressed in roots from both accessions under control conditions and were not differentially expressed at any stress condition. Their main functional categories were “secondary metabolism”, “signaling”, “protein”, “RNA”, “hormone metabolism”, “stress”, and “cell wall”. Comparing the expression levels of the two accessions under control conditions (R Ctrl vs S Ctrl), several genes were higher expressed in the resistant accession. Among the stress-related ones, we identified a chaperone DnaJ-domain superfamily protein (Phvul.001G226300), a MLP-like protein 43 (Phvul.005G058600), two NB-ARC domain-containing disease resistance proteins (Phvul.004G015800 and Phvul.008G071300), a multidrug resistance-associated protein 12 (Phvul.005G015500), a purple acid phosphatase 29 (Phvul.009G260300), a delta tonoplast integral (TIP) protein (Phvul.005G170300), a calmodulin-like protein 11 (Phvul.001G155400), and a signaling wall-associated kinase 2 (Phvul.001G066200).

#### Expression profiles of genes from selected functional categories

**Fig. 4:**
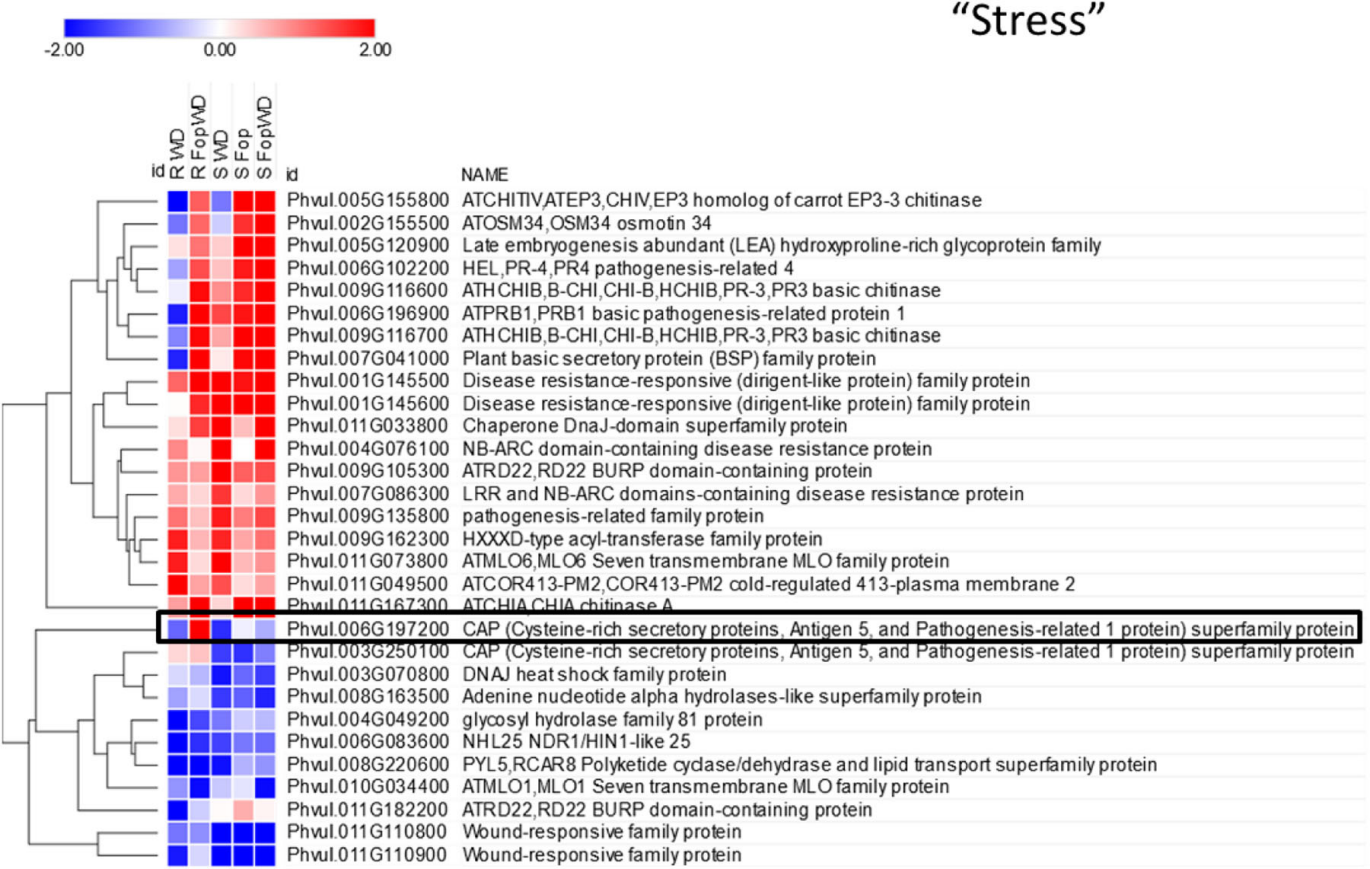
Heat map and hierarchical clustering of the root differentially expressed genes belonging to the “stress” functional category, in the resistant (R-645) and susceptible (S-1955) common bean accessions. WD = water deficit single stress, Fop = fusarium wilt single stress, FopWD = combined fusarium wilt + water deficit stress. The color scale represents the log_2_ fold change in relation to control conditions. The gene Phvul.006G197200 is highlighted due to its contrasting profile in the resistant accession for the combined *FopWD* condition

Heat maps with root gene expression profiles were analyzed separately for each gene functional category (Supplementary Fig. S5). As an example, the heat map and hierarchical clustering of the DEGs from the functional category “stress” are shown in Fig. 4. The gene Phvul.006G197200 coding for CAP (cysteine-rich secretory proteins, antigen 5, and pathogenesis-related 1 protein) superfamily protein is highlighted as the only up-regulated in the combined *FopWD* stress for the resistant accession.

#### DEGs with the highest expression levels

Ten genes had a log_2_ fold change superior to 5. The gene with the highest log_2_ fold change (6.58) was Phvul.005G071400 which codes for a low-molecular-weight cysteine-rich 68, up-regulated in the resistant accession under the combined *Fop*WD stresses. The second highest up-regulated (log_2_ fold change = 5.74) gene in the resistant accession was Phvul.003G096700 for WD single stress, which encodes for a drought-induced protein (Di21). Both genes were also up-regulated in the susceptible accession under the same stress conditions, but with lower log_2_ fold changes (4.74 and 3.17, respectively). The other eight highly-expressed root genes were identified for the susceptible accession. Two highly up-regulated genes (Phvul.009G161000 and Phvul.009G225000) encoded for integrase-type DNA-binding superfamily proteins, namely APETALA2/Ethylene-responsive element transcription factors. The first gene was up-regulated in the susceptible accession under *Fop* single stress and *Fop*WD, whereas the second was up-regulated in the susceptible accession under WD single stress. Also, Phvul.007G203400 that encodes a galactinol synthase 2 was highly up-regulated in the susceptible accession under *Fop* (log2 fold change = 5.57) and *Fop*WD (log_2_ fold change = 6.03). The following three genes were highly up-regulated in the susceptible accession under *Fop*WD: Phvul.001G067300 (log_2_ fold change = 6.05), Phvul.001G143100 (log_2_ fold change = 5.30), Phvul.009G211200 (log_2_ fold change = 5.33), coding for an alcohol dehydrogenase 1, a late embryogenesis abundant 4-5, and a bifunctional inhibitor/lipid-transfer protein/seed storage 2 S albumin superfamily protein. Finally, Phvul.007G081200 (log_2_ fold change = 5.45) and Phvul.009G240600 (log_2_ fold change = 5.01) had no available annotation and were up-regulated in the susceptible accession under *Fop* single stress and *Fop*WD, respectively.

#### Shared and unique DEGs between accessions and conditions

**Fig. 5:**
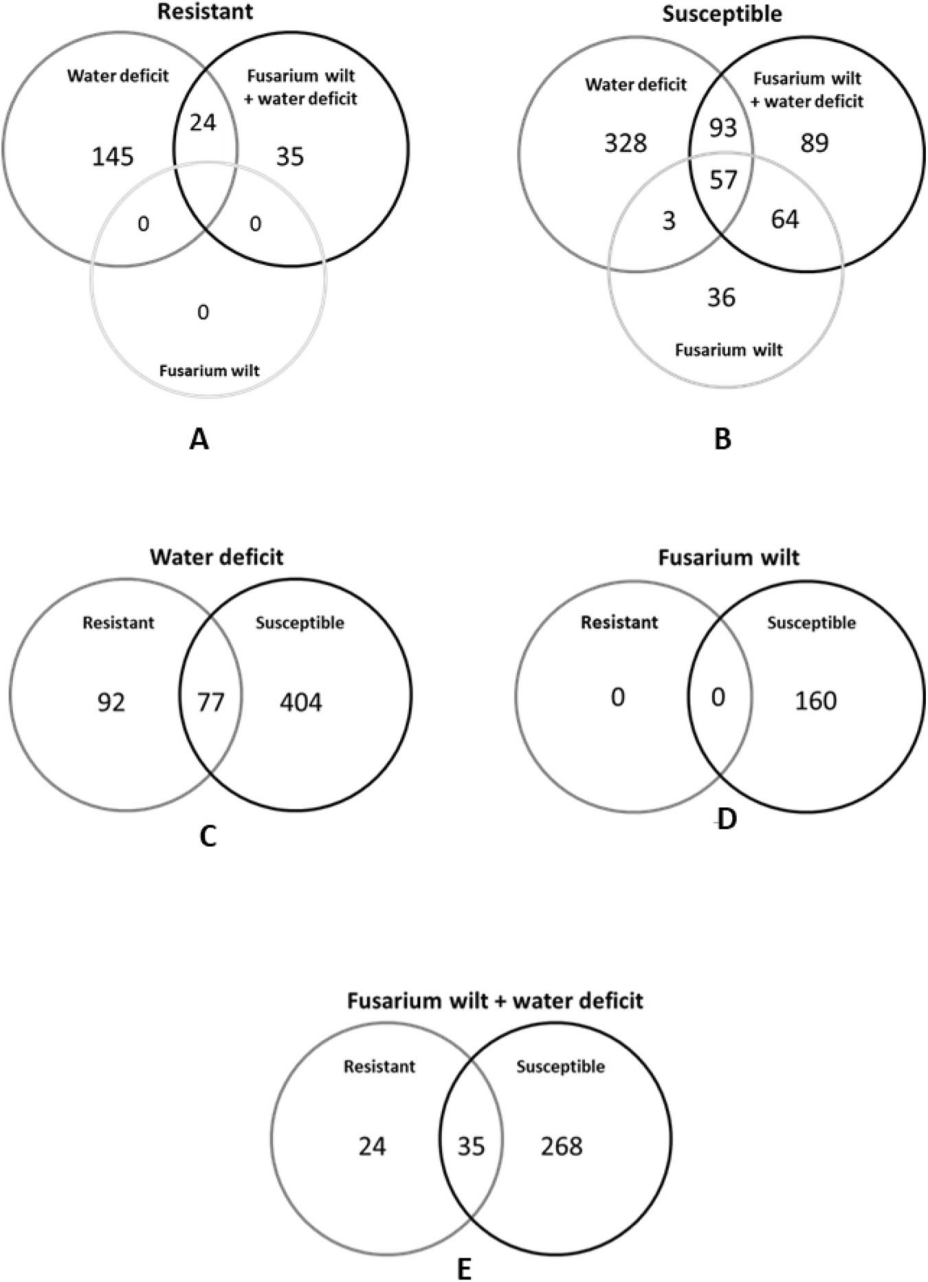
Venn diagrams with the unique and shared root differentially expressed genes (DEGs) in the common bean resistant accession (R-645) versus the common bean susceptible accession (S-1955), under the different treatments. **A** DEGs in the resistant accession; **B** DEGs in the susceptible accession; **C** DEGs under water-deficit conditions; **D** DEGs after *Fop* inoculation; and **E** DEGs under the combined stresses fusarium wilt + water deficit. Each treatment was compared with control conditions

In the resistant accession, 24 DEGs were found in common between WD and *Fop*WD, most of them down-regulated, belonging to the functional categories “cell wall”, “minor CHO metabolism”, “stress”, and “hormone metabolism” (Fig. 5A and Supplementary File S1). The up-regulated genes common to WD and *Fop*WD (Phvul.001G135200 and Phvul.007G203400) encoded for seed imbibition 2 (SIP2) and galactinol synthase 2, respectively, both belonging to the raffinose family. Five out of the 24 genes were exclusively differentially expressed in the resistant accession and included the already mentioned SIP2 (Phvul.001G135200), as well as a TT6 flavanone 3-hydroxylase (Phvul.003G261900), a PLAC8 family protein (Phvul.005G169200), a NDR1/HIN1-like 25 (Phvul.006G083600), and a TEM1 AP2/B3 transcription factor family protein (Phvul.007G102800). The remaining 19 DEGs were also identified in the susceptible accession with a similar expression pattern.

Still regarding the resistant accession, 35 DEGs were exclusively found under the combined stress condition. Their main functional categories were “transport”, “stress”, “secondary metabolism”, “hormone metabolism”, “RNA”, and “signaling”. Among the ones up-regulated, we identified a SIP2 (Phvul.001G135200), a UDP-glucosyl transferase 74B1 (Phvul.002G075200), an endonuclease 2 (Phvul.003G030500), a ribulose bisphosphate carboxylase (small chain) family protein (Phvul.004G064800), a CAP (cysteine-rich secretory proteins, antigen 5, and pathogenesis-related 1 protein) superfamily protein (Phvul.006G197200), a pectin lyase-like superfamily protein (Phvul.007G165300), a cytochrome P450 (Phvul.010G012700), and a chitinase A (Phvul.011G167300).

On the other hand, in the susceptible accession, 57 DEGs were found in common to all the three stressed conditions and mostly down-regulated (Fig. 5B and Supplementary File S1). Fourteen of the 57 DEGs were up-regulated and related to “stress”, “RNA”, “protein”, “hormone metabolism”, and “transport”.

Among the up-regulated root genes common to *Fop* and WD single stresses on the susceptible accession with a potential role on resistance/susceptibility, we identified a cytokinin response factor 4 (Phvul.008G246000), two disease resistance-responsive family proteins (Phvul.001G145500 and Phvul.001G145600), and a myb domain protein 15 (Phvul.007G273400). Among the down-regulated ones, we identified wound-responsive family proteins (Phvul.011G110800 and Phvul.011G110900), a beta-1,3-glucanase 1 (Phvul.001G128500), alcohol dehydrogenase 1 (Phvul.009G149500), a xyloglucan endotransglucosylase/hydrolase 32 (Phvul.001G179900), and a chloroplast-targeted copper chaperone protein (Phvul.002G207200).

Seventy-seven DEGs were common to both accessions under WD conditions (Fig. 5C). From those 77 DEGs, 41 were down-regulated and 34 were up-regulated in both susceptible and resistant accessions, whereas two were up-regulated in the resistant accession and down-regulated in the susceptible one. These two particularly interesting genes, Phvul.009G134300 and Phvul.004G054100, encode for a Nodulin MtN3 family protein and an unknown protein, respectively. From the 35 DEGs found common to both accessions under *Fop*WD (Fig. 5E), 17 were down-regulated and 18 up-regulated. On the other hand, three DEGs were down-regulated in the resistant accession under WD but in the susceptible accession under *Fop* and *Fop*WD were up-regulated. Those were Phvul.003G109800, Phvul.005G155800, and Phvul.009G140700 that coded for MLP-like protein 423, (ATCHITIV, ATEP3, CHIV, EP3) homolog of carrot EP3-3 chitinase, and a peroxidase superfamily protein, respectively.

Ninety-two DEGs were unique to the resistant accession under WD (Fig. 5A), belonging to the functional categories “protein”, “stress”, “RNA”, “transport”, “hormone metabolism”, “secondary metabolism”, and “signaling”. Among the ones upregulated were identified a lipoxygenase 3 (Phvul.002G228700), an ABA-responsive elements-binding factor 2 (Phvul.009G065500), a highly ABA-induced PP2C (Protein Phosphatase 2C) gene 2 (Phvul.008G231200) and highly ABA-induced PP2C gene 3 (Phvul.001G075400 and Phvul.001G23600), a HAB1 (Hypersensitive To Aba1) homology to ABI (Abscisic Acid-Insensitive)1 (Phvul.009G229900), a cold-regulated 413-plasma membrane 2 (Phvul.011G049500), a phytoene synthase (Phvul.008G241500), a sucrose synthase 3 (Phvul.001G209600), and a late embryogenesis abundant protein group 6 (Phvul.003G237400).

Thirty-five DEGs were common to both accessions under *Fop*WD, from which 17 down-regulated and 18 up-regulated in both accessions (Fig. 5E). On the other hand, 24 DEGs were unique to the resistant accession under *Fop*WD, and from those 11 were not differentially expressed in the susceptible accession under the imposed single stresses, being exclusive of the resistant accession under the combined stresses.

The above mentioned root genes are just a few examples of DEGs that might be involved in the resistance mechanisms deployed by common bean. The comprehensive list with all the DEGs identified is available in Supplementary File S1.

### Network analysis

**Fig. 6:**
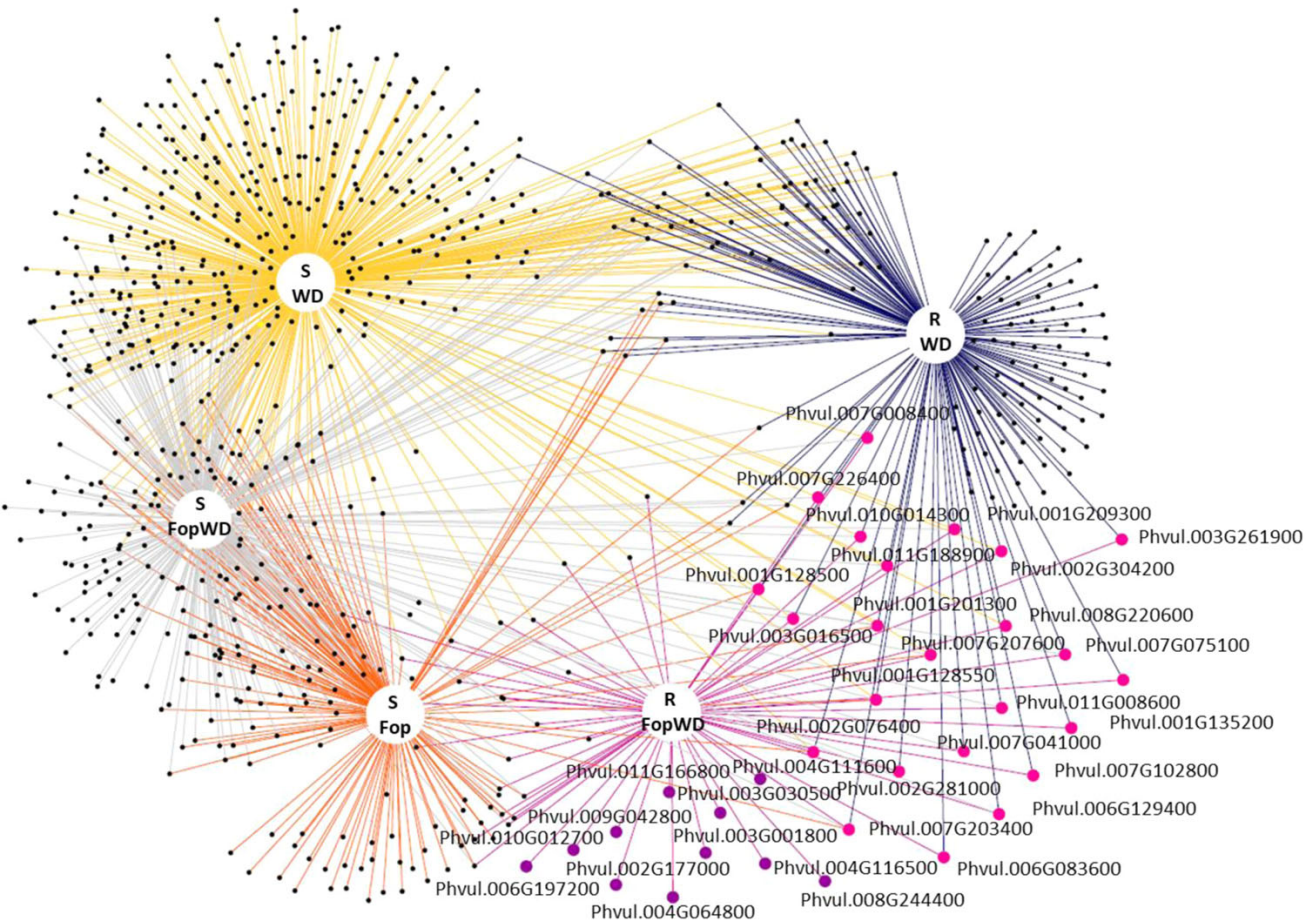
Network analysis of the differentially expressed root genes identified in each RNA MACE library. DEGs shared by single and combined stresses (pink circles), and unique DEGs for the combined stresses (purple circles), in the resistant common bean accession, are highlighted and respective gene IDs shown. R = common bean resistant accession (R-645), S = common bean susceptible accession (S-1955), WD = water deficit single stress, Fop = fusarium wilt single stress, FopWD = combined fusarium wilt + water deficit stresses

Network analysis was performed to better visualize connections between the DEGs at each condition (Fig. 6). It was clear that several genes bridged responses between the applied stresses in both accessions (potential hub genes, with a role in different stress responses), whereas others were expressed only under the combined stress (potential tailored responses). Also on both accessions and in the case of WD, the majority of the DEGs under this single stress are not shared with the combined stress condition. Finally, the susceptible accession responded more actively (higher number of DEGs) to the stresses imposition (individually or combined) than the resistant accession.

### MACE validation by qRT-PCR

The expression stability (M-value) of the potential reference genes for the qRT-PCR validation using the geNorm and NormFinder software package was tested. The best reference genes were Phvul.002G104100, encoding for RNA-binding (RRM/RBD/RNP motifs) family protein, and Phvul.003G082700, encoding for indole-3-butyric acid response 1, both with M-value (anti log_2_) of 0.4 (Supplementary File S2).

**Fig. 7:**
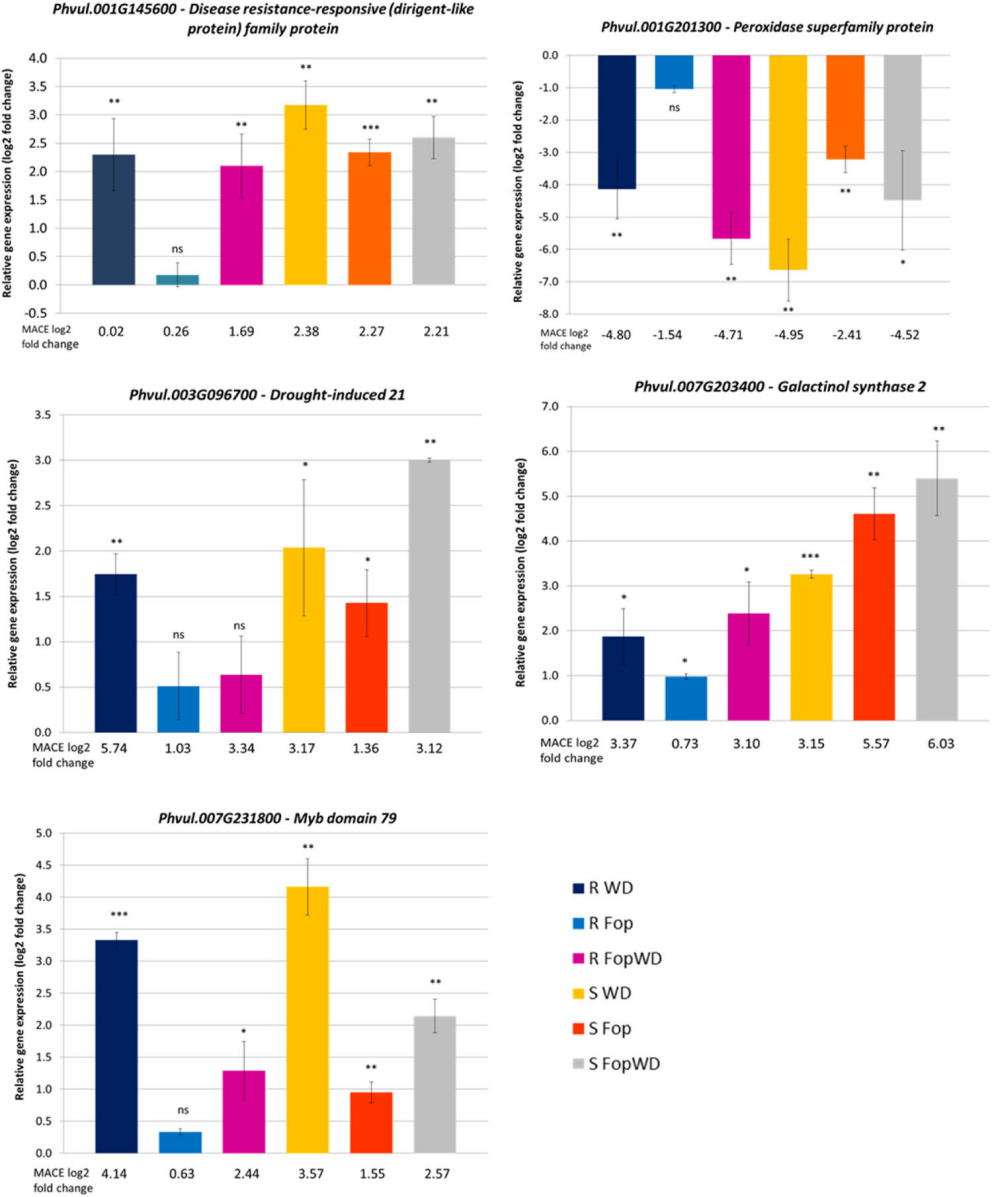
Expression patterns of five root genes in the common bean resistant (R-645) and susceptible accession (S-1955), under three treatments, obtained by quantitative real-time PCR. The log_2_ fold change values are represented in relation to the non-inoculated and well-watered control. A comparison with MACE log_2_ fold change is shown. R = resistant accession, S = susceptible accession, WD = water deficit single stress, *Fop* = fusarium wilt single stress, *Fop*WD = combined fusarium wilt + water deficit stress. Error bars correspond to the standard deviation of the mean of the three biological replicates. Paired Student’s t-test indicates the significance between the expression levels of each sample in relation to control samples. ns – not significant α > 0.05, * 0.01 ≤ α < 0.05, ** 0.001 < α < 0.01, *** α ≤ 0.001

To validate the MACE results, the expression of five selected DEGs were analyzed by qRT-PCR, using three root biological replicates per stress condition and normalized to the control conditions (Fig. 7).

A high Pearson’s correlation (0.93) was observed between the log_2_ fold change values obtained by MACE and qRT-PCR for the five genes in all conditions. Additionally, linear regressions were fitted to compare log_2_ fold change values obtained by MACE and qRT-PCR separately for each accession. The R^2^ values of the regressions were 0.75 and 0.96, for the resistant and susceptible accessions, respectively (Supplementary Fig. S6).

## Discussion

A better knowledge of the molecular mechanisms behind plant multiple stress interactions is important for a more precise breeding because plants exhibit tailored physiological and molecular responses when exposed to simultaneous stresses, which are not the addition of the responses observed in individual stresses imposition^10^. Fusarium wilt (*Fop*) and water deficit (WD) are two stresses frequently concurrent in nature in common bean^29^. However, few studies exist on the interaction of abiotic and biotic stresses in this crop, and a scarce understanding of key genes and signaling pathways triggered by concurrent stresses is available. To identify shared and unique phenotypic and transcriptomic responses to fusarium wilt and early water deficit, a double resistant and a double susceptible common bean accessions were analyzed under the two stresses applied individually or in combination. Overall, common bean showed to have both shared (hub genes) and unique (tailored) responses to combined biotic and abiotic stresses in comparison to single stress imposition, facilitating the identification of interesting targets for multiple stress resistance breeding, and clearly anticipating a more complex response to abiotic-biotic multiple stresses than to single stress imposition in this crop.

The combined fusarium wilt and early water deficit stress (*Fop*WD) imposition resulted in a stronger impact in the photosynthetic performance compared to plants only subjected to *Fop* and even more to WD single stresses. Also visually, the combined stress imposition enhanced leaf damage (necrosis as well as chlorosis) mainly on the susceptible accession. Overall, photosynthetic responses such as CO_2_ assimilation rate (A), transpiration rate (E), and stomatal conductance (gs) decreased more under *Fop*WD than under *Fop* and especially than under WD single stresses, when compared to control conditions. Interestingly, the photosynthetic pigment concentrations decreased under WD single stress, increasing under *Fop* single stress, with the highest value observed under the combined *FopWD* stress. This is indicative that the response to the combined stresses is not simply the sum of the individual responses to single stresses, which is in agreement with the conclusions of a review of multi-stress studies^5^. In our study, the phenotypic biotic (*Fop*) stress responses were more similar to the combined stress imposition than the abiotic (WD) single stress responses. The same trend was observed at the transcriptional level, with combined stress activating unique responses not triggered by individual stresses. Moreover, early WD single stress showed a much higher number of DEGs than *Fop*WD or *Fop* single stress, not sharing the majority of its DEGs with the other conditions, anticipating a more complex and differentiated transcriptomics response under WD. Nevertheless, the most common functional categories of the genes differentially expressed were identical to all the stresses applied, with the majority of the annotated DEGs belonging to “RNA”, “signaling”, “protein”, “secondary metabolism”, “hormone metabolism”, “cell wall”, “transport”, and “stress” categories.

The assessment of the accessions photosynthetic performance allowed us to choose T2 (96 h) as the time point for gene expression analysis since this was the time point that in general showed the highest variation in pigment contents and gas exchange parameters among accessions and treatments.

As for the physiological evaluation, the two phenotypic contrasting accessions were also clearly distinguishable at transcriptomic level with higher gene expression levels detected in the susceptible accession after 96 h of stress imposition. Indeed, the total number of DEGs, in relation to control conditions, was four times higher for the susceptible accession than for the resistant accession. Similar results were described in common bean response to a cyst nematode infection (caused by *Heterodera glycines* Ichinohe) where 353 DEGs were found in a resistant accession and 990 DEGs in a susceptible accession^30^.

In the scope of this discussion, it is not possible to discuss every DEGs identified in this work. Thus, we will highlight the ones with higher fold change, and the ones exclusive of the resistant accession or with a contrasting differential expression on the susceptible accession, common to the different stresses or specific to the combined stresses studied. We will focus mainly on those whose functional annotation seemed to be more relevant to unveil the molecular mechanisms behind the studied stress interactions.

### DEGs with higher fold change

Among the ten genes with a log_2_ fold change superior to 5, only two were up-regulated in the resistant accession. The gene with the highest log_2_ fold change (6.58) coded for a low-molecular-weight cysteine-rich 68, up-regulated in the resistant accession under the combined *Fop*WD stresses. This protein belongs to the plant defensin family that includes many antimicrobial peptides involved in host plant resistance to pathogens, such as fungi^31^. The second highest up-regulated (log_2_ fold change = 5.74) gene in the resistant accession for WD single stress encoded a drought-induced protein (Di21). Both of these two genes were also up-regulated in the susceptible accession under the same stress conditions, but with lower log_2_ fold changes (4.74 and 3.17, respectively). Other eight highly-expressed genes were identified for the susceptible accession. Two highly up-regulated genes encoded for integrase-type DNA-binding superfamily proteins, namely APETALA2/Ethylene-responsive element transcription factors. The first gene was up-regulated in the susceptible accession under *Fop* single stress and *Fop*WD, whereas the second was up-regulated in the susceptible accession under WD single stress. These transcription factors are integral components of signaling cascades and regulate the expression of genes related to stress response through different mechanisms^32^.

### DEGs shared by single and combined stresses in the resistant common bean accession

No DEGs were identified in the resistance accession response to *Fop* with the log_2_ fold change, FDR, and p-values threshold applied.

Twenty-four common players were identified in the resistant accession response to early WD and *Fop*WD. Only two of them were up-regulated genes and both coded for proteins belonging to the raffinose family. Seed imbibition 2 and galactinol synthase 2 are raffinose family oligosaccharides reported to have a role in plant survival after exposure to stress conditions, for instance, drought, acting as osmoprotectants in plant cells^33^. Those genes might be considered candidate hub genes for biotic and abiotic stresses, with a potential application on multiple stress breeding, and their importance in the stress network, together with their biosynthesis pathways, should be further explored. It is worth mentioning that both up- and down-regulated DEGs were assigned to multiple functional categories, disregarding the accession and the stress applied.

### Unique DEGs in the combined FopWD stresses in the resistant common bean accession

From the 24 DEGs from the resistant accession identified only in the combined *Fop*WD condition (not present in the single stress conditions), 11 were exclusive and not expressed in the susceptible accession under any of the stresses applied, 5 up-regulated and 6 down-regulated. The remaining DEGs that were also identified in the susceptible accession under single stresses did not reveal a contrasting expression profile comparing to the resistant accession.

As indicative of the complex integrated regulatory network involved in multiple stress response, the up-regulated genes identified in the resistant accession under *Fop*WD were assigned to five different functional categories: “stress”, “photosystem (PS)”, “hormone metabolism”, “cell wall”, and “minor CHO metabolism”. Among the up-regulated, we found a CAP (Cysteine-rich secretory proteins, Antigen 5, and Pathogenesis-related 1 protein (PR-1)) superfamily protein. The implication of PR-1 in sterol binding and the identification of the CAP-derived peptide suggest multiple roles in immune defense, from antimicrobial function and defense signal amplification to potential sterol or effector recognition^34^. Moreover, a ribulose bisphosphate carboxylase (small chain) family protein was also up-regulated only in the combined stresses for the resistant accession. RuBisCO is the major protein in the stroma of chloroplasts and in higher plants exists as a complex of 8 large and 8 small subunits. The small subunit is coded by several different genes, which are distributed in a tissue-specific manner, but their function is unknown^35^. The increase in the abundance of its small subunit transcripts may indicate an increased RuBisCO synthesis, and thus an improved photosynthetic rate. Finally, in this particular subset of DEGs, we identified a CYP76C4 cytochrome P450, family 76, subfamily C, polypeptide 4. In *Arabidopsis*, several cytochrome P450 genes were induced by both abiotic and biotic stresses and are known to participate in the regulation of plant defense^36^. CYP76C4 cytochrome P450 seems to be involved in the terpenoid metabolism and has been previously reported as expressed in *Arabidopsis* roots^37^. It is known that several terpenoids have a role in plant defense against biotic and abiotic stresses^38^. On the other hand, the down-regulated genes were assigned to the same functional categories plus signaling, transport, secondary metabolism, and RNA which included two transcription factors (APETALA2/Ethylene-responsive element-binding protein family, and myb domain protein 19) regulators of stress tolerance^39^.

### Unique DEGs in single stress in the resistant common bean accession

The resistant accession under single WD stress revealed 145 DEGs (not present in the combined *Fop*WD stresses), 58% of them up-regulated. From those 145, 69 were also identified in the susceptible accession under at least one of the stresses applied. Only four had a contrasting expression pattern between accessions. We identified up-regulated in the resistant and down-regulated in the susceptible accession, a nodulin MtN3 family protein previously reported to modulate plants’ physiological response to environmental stresses by facilitating ion transport via interaction with ion transporters or as sugar transporters^40^. On the other hand, we identified, down-regulated in the resistant and up-regulated in the susceptible accession, a MLP-like protein 423, a peroxidase superfamily protein, and a ATCHITIV, ATEP3, CHIV, EP3 homolog of carrot EP3-3 chitinase.

Interestingly, in our work, the only ABA-induced genes differentially expressed were detected in the resistant accession under water deficit (single stress). HAI2 highly ABA-induced PP2C gene 2 and HAI3 highly ABA-induced PP2C genes 3 encode protein phosphatases described to be involved in the negative regulation of ABA signaling in *Arabidopsis*^41^. It is well reported that ABA, a major phytohormone, is involved in the regulation of abiotic stress pathways in plants and that plants have to adjust ABA levels constantly in response to changing physiological and environmental conditions, experienced, for instance, under drought^42^.

For the resistant accession, no DEGs were identified in *Fop* single stress in comparison to the control condition at 96 h after inoculation. This result suggests that this accession may display a constitutive defense against the single stress, with resistance genes constitutively expressed. In agreement with this, in *Arabidopsis*, constitutive expression of ERF (Ethylene Responsive Factor) 1 was described to mediate and confer enhanced resistance to *F. oxysporum* f. sp. *conglutinans* and *F. oxysporum* f. sp. *lycopersici*^43^. The presence of a basal resistance in this accession, activated independently of infection, might have hampered DEGs detection. There is also the possibility that this time point, although with the highest photosynthetic response variation among accessions and treatments, was not optimal to detect DEGs in response to *Fop* in the resistant common bean accession we tested. Studies in cucumber and banana, for example, reported transcriptional responses to *Fusarium oxysporum* spp. at a wide range of time points, from 24 h until 14 days after inoculation^44,45^. A histological assay to follow the fungus infection process could help in the future to choose a more adequate time point. Finally, another possible justification is the variation found among biological replicates of *Fop* MACE libraries in the resistant accession that might have led to high FDR values. Since the FDR cut value we used was a stringent one, only the most reliable expression changes were considered.

### Genes expressed by both common bean accessions under control and not differentially expressed under stress conditions

Several stress-related genes were simultaneously expressed under control conditions by both accessions. Among the higher expressed in the resistant accession in relation to the susceptible one, we identified a MLP-like protein 43 described as a positive regulator during ABA responses and able to drought tolerance in *A. thaliana*^46^; two NB-ARC domain-containing disease resistance proteins that act as molecular switches in plant defense mechanism; a purple acid phosphatase (PAP) 29 with several PAP implicated in plant defense responses, as for example PAP5 described to maintain basal resistance against *Pseudomonas syringae* in *Arabidopsis*^47^; a delta tonoplast integral (TIP), reported to have a special role in regulating the flow of water and nutrients during drought stress^48^; a calmodulin-like 11 which is an important Ca^2+^ sensor, playing a significant role in mediating plant stress tolerance^49^; and a wall-associated kinase 2 reported to participate in basal defense against rice blast fungus^50^.

To conclude, this study demonstrated, using phenotypic and transcriptomic data, that common bean response to a combination of stresses is not the sum of the responses to the individual stresses, and because of that, cannot be predicted from individual single stress analysis. The combined stresses led to the activation of a gene network where the most representative functional categories were “stress”, “signaling”, “cell wall”, “hormone metabolism”, and “secondary metabolism”. Some genes were activated in both single and combined stresses for both accessions and might be considered candidate hub genes for stress response. The hub DEGs exclusive of the resistant accession were assigned to the functional categories “minor CHO metabolism”, “RNA”, “secondary metabolism”, and “stress”.

Several genes were exclusively differentially expressed in a tailored response when *Fop* and early WD stresses were combined. Among the ones up-regulated with higher expression in the resistant accession, we identified a CAP (Cysteine-rich secretory proteins, Antigen 5, and Pathogenesis-related 1 protein) superfamily protein involved in plant immune response^51^; a ribulose bisphosphate carboxylase (small chain) family protein whose activity was already related to drought stress^52^; and a chitinase A with multiple physiological roles and whose production was described to be induced in response to biotic and abiotic stress factors^53^. Those genes have great potential to be explored in multiple stresses breeding as they are involved in the more complex tailored responses to both abiotic and biotic stresses. Moreover, we verified that the number of DEGs was much higher under WD conditions than under *Fop* stress anticipating a more complex response to water deficit than to fusarium wilt in common bean. This is in agreement with previous reports that showed that resistance to pathogens seems to be genetically simpler than tolerance to abiotic stresses, such as drought^54^.

The development of multi-stress-tolerant varieties will be accomplished with the combining introgression of several genes/QTLs for abiotic and biotic stresses. This might be achieved in a more efficient way through genomic selection than via marker-assisted selection^55^. The recent advances in genomic selection research for complex traits in many crops, including legumes^56^, foresee an increase in the efficiency of breeding for multi-stress resistance^57^. In the case of common bean, the availability of resistant accessions from both gene pools—Andean and Mesoamerican—and of intermediate origin, as the ones identified within the Portuguese germplasm^27,28^ might bring favorable genetic combination useful for multi-stress breeding.

Overall, our results provide clues to further understand the regulation of plant response to simultaneous stresses. The stress-related DEGs identified in this study implicated in common bean water deficit and *Fop* infection response, allow a better understanding of the mechanisms of defense and resistance to these stresses and will support the selection of a set of genes more likely to be major regulatory hubs, or main players on tailored responses that can be used as targets for multiple resistance selection.

## Material and methods

### Plant material and growing conditions

Two Portuguese common bean accessions—R-645 of Andean origin, and S-1955 of Mesoamerican origin—were chosen based on their contrasting response to water deficit and fusarium wilt^27,28^. Accession R-645 is resistant and accession S-1955 is susceptible to both stresses. The disease severity score (DS visual scale 1–5, where 1 represents no symptoms and 5 represents a dead plant) 30 days after inoculation with *Fusarium oxysporum* f. sp. *phaseoli* (isolate FOP-SP1 race 6, herein called *Fop*) was 1.8 for accession R-645, and 5.0 for accession S-1955 (ref. ^27^). Also, accession R-645 was more resilient than S-1955 to water deprivation, with photosynthesis-related traits showing higher variation in the latter accession when comparing plants, with the second trifoliate leaf established, under well-watered and water deficit conditions. For example, in accession R-645, the net CO_2_ assimilation rate was only 10% lower under water deficit (defined as 40% of field capacity) than under well-watered conditions, while for accession S-1955 it was 3.4 times lower under water deficit^28^.

Thirty-six seeds per accession were sown, 72 seeds in total, one seed per pot, and pots were placed in trays in a growth chamber kept at 26 ± 2 °C during day and 18 ± 2 °C during night, under a photoperiod of 16 h light (~ 295 μmol m^−2 ^s^−1^) and 8 h dark, with a relative humidity of 50% and a CO_2_ concentration of 370 ppm, approximately. Sowing was done in 8 × 8 × 9 cm plastic pots (0.5 L), filled with vermiculite, which were previously watered to full water capacity and weighted. Three pots were filled with dry vermiculite and weighed to estimate the value of the dry weight of the pots. This dry weight value was used to calculate the soil water content of each pot during the experiment.

### Experimental design

The seventy-two pots were divided into three groups of 24 pots for stress response phenotypic evaluation and root sampling for RNA extraction at three different time points − 48 h (T1), 96 h (T2), and 8 days (T3) after stress imposition. Stress was imposed when the first trifoliate leaf was fully expanded (V1 growth stage). At each time point, 12 plants per accession were phenotypically evaluated as follows: three plants were evaluated for response to soil water deficit, defined as 40% of field capacity, three other plants for response to fusarium wilt (*Fusarium oxysporum* f. sp. *phaseoli* inoculation), three other for the two stresses combined imposition, and the final three plants were used as controls with no stress applied (Supplementary Fig. S7).

### Water deficit imposition

All pots were watered every other day with tap water, to keep the well-watered conditions until the first trifoliate leaf was fully expanded. At the defined growth chamber conditions and for the type of substrate used (vermiculite), three days without watering were enough for the pots to reach the 40% of field capacity. Based on this, the water supply was interrupted 3 days before the *F. oxysporum* inoculation day for the plants under the water deficit treatment (single or combined). These plants were then kept at 40% of field capacity until the end of the experiment, by daily weighing the pots and adding the amount of water needed to reach this percentage. The remaining plants (fusarium wilt single stress and control group) were kept under well-watered conditions until the end of the experiment.

### Fungal isolate and inoculation

*Fusarium oxysporum* f. sp. *phaseoli* isolate FOP-SP1 race 6 was kindly provided by Prof. José María Díaz Mínguez (University of Salamanca, Spain), and stored as microconidial suspensions at −80 °C in 30% glycerol. This fungal strain was identified in common bean cultivars in Avila, Spain, and classified as highly virulent^21^.

Microconidia multiplication followed the protocol described in Leitão et al.^27^. Briefly, the fungal culture was let to grow at 28 °C under constant shaking (170 rpm), for 4 days, and a suspension of 5.0 × 10^6^ conidia mL^−1^ was prepared to be used on the same day to inoculate the common bean seedlings.

For the inoculation, seedlings were removed from the pots, vermiculite was cleaned out of roots that were then immersed in the fungal suspension for 30 min. Roots from control plants were immersed for 30 min in water, to mimic the stress of immersing the root in the fungal suspension during inoculation. Seedlings were re-planted in the pots and maintained in the same growth chamber, under the same photoperiod and temperature conditions, until sampling.

To verify the inoculation success and the full development of the disease in the susceptible accession, three extra plants of each accession were inoculated together with the trial plants and kept under well-watered conditions for a month.

### Photosynthetic performance

At the three defined time points after stress imposition and before root sampling, gas exchange photosynthetic parameters—stomatal CO_2_ conductance (gs), net CO_2_ assimilation (A), and transpiration rate (E)—were measured in every plant of each treatment. The measurements were carried out using a portable Infra-Red Gas Analyzer system (IRGA, LCpro+ ADC BioScientific Ltd., Herfordshire, UK), with controlled atmosphere (approximately 370 μmol mol^−1^ CO_2_ concentration, 23 ± 2 °C and relative humidity of 50–60%) and a saturating external light source of 1044 μmol m^−2^ s^−1^. Measurements were made in the non-detached first trifoliate fully-expanded leaf in plants at V1 to V2 growth stage, depending on the time point. A, E, and gs values were used to calculate instantaneous and intrinsic water use efficiencies (A/E and A/gs, respectively).

### Leaf photosynthetic pigments

Leaf photosynthetic pigments—chlorophylls *a* (C*a*) and *b* (C*b*), and carotenes and xanthophylls (C*cx*)—were quantified for all the plants^58^, at each of the three time points. Briefly, two leaf discs, with 0.636 cm^2^ each, were sampled from the same first trifoliate leaf used in the previous gas exchange measurements, immediately submerged in 95% ethanol, and kept in the dark at 4 °C until full extraction of the pigments. The absorbance of the extract was measured at 470, 648.6, and 664.1 nm in an Ultrospec 4000 UV–visible spectrophotometer (Pharmacia Biotech), and the concentrations of C*a*, C*b*, and C*cx* estimated. The sum of C*a* and C*b*, their ratio, and the ratio between the sum of chlorophylls and carotenes and xanthophylls [(C*a* + C*b*) /C*cx*] were subsequently calculated to characterize the plants’ physiological state.

### Leaf water status

Leaf relative water content (RWC) was calculated to estimate the water status of the leaves^59^ at the time of the gas exchange measurements. Three discs per plant were punched out of leaves and weighed immediately to obtain the fresh weight (FW). Then, leaf discs, with 0.636 cm^2^ each, were put floating in distilled water, in the dark, at 24 °C overnight to obtain the turgid weight (TW). Finally, the discs were oven-dried at 80 °C, until constant weight to obtain the dry weight (DW). RWC was calculated using the formula RWC (%) = [(FW−DW)/(TW−DW)] × 100.

### Phenotypic data analysis

The restricted maximum likelihood (REML) framework of Genstat® software, 19th edition (VSN International, Hemel Hempstead, UK) was followed with accession (resistant, susceptible), treatment (control, fusarium wilt, water deficit, combined stresses fusarium wilt + water deficit) and time point (48 h, 96 h, and 8 days after stress imposition) as factors to determine the variance components and consequently the contribution of their effects in the variation of each of the 12 traits measured (RWC, A, E, gs, A/E, A/gs, C*a*, C*b*, C*cx*, C*a* + C*b*, C*a*/C*b*, (C*a* + C*b*)/C*cx*). A Wald test for fixed effects was performed and their significance, as well as of the interaction between factors, was evaluated. A Tukey’s post-hoc multiple comparison test at a significance level of 95% was used for means comparison between accessions, treatments, and time points.

Phenotypic correlations (Pearson’s r) were calculated between traits, and principal component analysis (PCA) was performed based on the eigenvalue decomposition of the correlation matrix obtained.

### Root sample collection, RNA isolation, quantification, and quality assessment, and cDNA synthesis

After trait measurements at each of the three time points, plants from all treatments were removed from the pots and quickly washed under tap water to remove the vermiculite. Root samples were collected from individual plants, immediately frozen in liquid nitrogen, and stored at −80 °C for the subsequent molecular analysis. Sampling was performed 48 h, 96 h, and 8 days after single and combined stress imposition. Roots from control plants were also sampled at the same time points.

For total RNA isolation, frozen roots were ground to a fine powder in liquid nitrogen using a mortar and pestle. Total RNA was isolated separately for each of three plants of the different accessions and treatments, using the GeneJET^TM^ Plant RNA Purification Mini Kit (Thermo Scientific^TM^, Massachusetts, USA) according to the manufacturer’s protocol. Trace amounts of DNA contamination were removed from RNA after treatment with TURBO^TM^ DNase (Invitrogen^TM^ by ThermoFisher Scientific^TM^, California, USA), following the manufacturer’s instructions. RNA quantification was performed using Qubit RNA BR (Broad-Range) Assay Kit *(*Life Technologies^TM^, California, USA) on a Qubit 2.0 Fluorometer (Invitrogen^TM^, ThermoFisher Scientific^TM^, California, USA). RNA purity was estimated based on the 260/280 and 260/230 absorbance ratios using a NanoDrop^TM^ 2000c Spectrophotometer (Thermo Scientific^TM^, Passau, Germany), which were 2.1 and 1.8 on average, respectively, after DNAse treatment. RNA integrity was also checked by electrophoresis in a 1% agarose gel stained with SYBR^TM^ Safe (Life Technologies^TM^, California, USA). cDNA was synthesized from one μg of total RNA from each sample following the manufacturer’s instructions of the iScript™ cDNA synthesis kit (Biorad, California, USA).

### Massive analysis of cDNA ends (3′ mRNA-Seq) and data analysis

Total RNA isolated from roots 96 h after inoculation from three plants per accession and treatment, totalizing 24 samples (2 accessions × 4 treatments × 3 biological replicates), was sent to RNA-seq variant MACE—massive analysis of cDNA ends—provider (GenXPro GmbH, Frankfurt am Main, Germany). This time point (96 h after stresses imposition) was chosen since it showed the largest photosynthetic related phenotypic differences between resistant and susceptible accessions (see Result section).

Triplicated MACE libraries for each accession and treatment were prepared and sequenced by the service provider following in-house developed protocols^60^. Poly-adenylated mRNA was isolated from 1 µg of the large fraction of total RNA using Dynabeads® mRNA Purification Kit (ThermoFisher Scientific^TM^, California, USA). First- and second-strand synthesis of cDNA was performed using SuperScript® III First-Strand Synthesis System (Invitrogen^TM^, ThermoFisher Scientific^TM^, California, USA), with modified bar-coded 5′-end biotinylated poly-T adapters suitable for the Illumina Hiseq2000 flow cell (Illumina, San Diego, USA). Subsequently, the cDNA was fragmented to yield 250 base pair (bp) fragments. The 3′-ends of the fragmented cDNA were captured with streptavidin beads, while PCR bias-proof technology “TrueQuant” was used, by ligation of TrueQuant adapters (GenXPro GmbH, Frankfurt am Main, Germany), to distinguish PCR copies from original copies. The barcoded samples were sequenced simultaneously in one lane of an Illumina Hiseq2000 with 1 × 100 bps. Low-quality sequence-bases were removed using “cutadapt” tool^61^. Poly(A)-tails were clipped by an in-house Python-Script.

The reads were aligned to “Pvulgaris_442_v2.0.fa.gz” (from Phytozome v12.1 *P. vulgaris* v2.1, http://phytozome.jgi.doe.gov/) using Bowtie 2^62^. The annotation information was taken from the files “Pvulgaris_442_v2.1.gene.gff3” and “Pvulgaris_442_v2.1.annotation_info.txt” (these sequence data were produced by the US Department of Energy Joint Genome Institute, DOE-JGI and USDA-NIFA). Normalization and test for differential gene expression between libraries were performed using DEGseq R-package version 1.16.0 (ref. ^63^). Genes were considered expressed when they presented a raw read value number ≥ 100 in the three biological replicates of at least one experimental condition of the comparison. Differential gene expression was quantified as the log base 2-fold change (log_2_FC) of the ratio of normalized values in pair-wised comparisons. The *P*-value and correction for multiple testing with the Benjamimi-Hochberg false discovery rate (FDR) were computed to determine the significance of gene expression differences. Genes were considered differentially expressed (DEGs – differentially expressed genes) between conditions when the |log_2_FC | ≥ 1.5, *P*-value ≤ 0.05, and FDR ≤ 0.01.

Within each accession, transcriptome comparisons were made between control conditions and the three studied treatments—water deficit single stress (WD), *Fop* single stress (*Fop*), and the combined stresses (*Fop*WD)—to extract DEGs with changes induced by the applied treatments. To identify the pre-stress imposition gene expression differences between genotypes, a comparison between resistant and susceptible accessions for the control treatment was also established. All raw sequencing data have been deposited in NCBI sequence read archive (SRA) database under the project PRJNA694365 with SRA numbers SAMN17517910- SAMN17517933.

### Sequencing data validation by quantitative real-time PCR

#### Gene selection and primer design

Five target genes were selected from the DEGs dataset for MACE data validation by quantitative real-time PCR (qRT-PCR): Phvul.001G145600—Disease resistance-responsive (dirigent-like) family protein, Phvul.001G201300—RCIA peroxidase superfamily protein, Phvul.003G096700 Drought-induced 21, Phvul.007G203400—GolS2 galactinol synthase 2, and Phvul.007G231800—MYB79 myb domain protein 79. These genes were selected based on their level of expression and transcript count, to represent a broad range of expression profiles (Supplementary File S3).

The expression stability (M-value) of eight potential reference genes was tested using the geNorm and NormFinder software package, from GenEx v.5 software (MultiD, Goteborg, Sweden),

Specific primers were designed for reference and target candidate genes using the Primer3Plus online tool (https://primer3plus.com/) (Boston, USA), and checked for specificity using the Primer-BLAST NCBI tool (National Center for Biotechnology Information, USA). Primer design parameters were defined using the default setting for qRT-PCR optimal conditions, on Primer3Plus online tool. Primers were designed in the 3′ intra-exonic region and were synthesized by STABVida (Caparica, Portugal) (Supplementary Table S5).

#### Quantitative real-time PCR

The relative expression of the five selected target genes was determined by quantitative real-time PCR (qRT-PCR) to validate RNA-sequencing (MACE). The qRT-PCR reactions were performed using three biological replicates per accession (resistant and susceptible) and treatment (Ctrl, WD, *Fop*, and *Fop*WD) for the time point 96 h (T2) after stress imposition, in a total of 24 samples. The qRT-PCR reactions were carried out in a PikoReal™ Real-Time PCR System (Thermo Scientific^TM^) using PerfeCTa® SYBR® Green SuperMix™ (Quantabio, Massachusetts, USA). Primer PCR efficiencies were tested for all primer pairs using a 10-fold dilution series of the cDNA samples. Each reaction of 10 µL was performed twice (technical replicates) containing 2.5 ng of cDNA, 200 nM of each primer, and 5 µL of PerfeCTa® SYBR® Green SuperMix™. Thermal cycling for target and reference genes started with a denaturation step at 95 °C for 5 min, followed by 40 cycles of denaturation at 95 °C for 10 s and 60 °C for 30 s. For each reaction, a melting curve (dissociation stage) was performed to verify non-specific PCR products or contaminants. Also, a negative template control (NTC) without cDNA was included in each PCR plate to detect possible contaminations.

The relative expression values (fold change) of the five target genes were normalized to the control samples and to the two reference genes using the Pfaffl method^64^. Finally, fold change data were transformed into a logarithmic scale (base 2) for graphical representation and statistical analyses. Student’s t-tests were performed to compare the expression levels of each sample in relation to the control.

### Bioinformatic data analysis

A principal component analysis (PCA) with normalized reads counts of DEGs as loading vectors was used to assess the relative distribution of all biological replicates of each experimental condition, using Genstat® software, 19th edition (VSN 2017).

The functional categorization of DEGs was performed using MapMan web tools (Max Planck Institute for Molecular Plant Physiology, Golm, Germany). Unspliced gene sequences of *P. vulgaris* genome version v2.1 retrieved from Phytozome v12.1 were used to create a mapping file for the Mercator pipeline from MapMan.

Venn diagrams were set up using the Bioinformatics & Evolutionary Genomics platform (http://bioinformatics.psb.ugent.be/webtools/Venn/) to compare DEGs identified between each accession/treatment.

Gene expression profiles were analyzed and heat maps produced with Morpheus software (https://software.broadinstitute.org/morpheus).

Cytoscape software^65^, version 3.8.0, was used to visualize the molecular interaction networks associated with each RNA MACE library.

Pearson’s correlation was calculated between the log_2_ fold change values obtained by MACE and qRT-PCR for the five selected target genes in all conditions. Additionally, linear regressions were fitted to compare log_2_ fold change values obtained by MACE and qRT-PCR separately for each accession.

## Supplementary information

Supplementary Figures S1-S7

Supplementary Tables_S1-S5

Supplementary File S1_Raw data and DEGs

Supplementary File S3_genes validation MACE

Supplementary File S2_M value
