# Supplementary material for: Shared and tailored common bean transcriptomic responses to combined fusarium wilt and water deficit
Source: Hortic Res. 2021 Jul 1;8:149. doi: 10.1038/s41438-021-00583-2 (PMC8245569; doi:10.1038/s41438-021-00583-2)
Supplement: Supplementary file 1 — Supplementary Figures S1-S7 [file 41438_2021_583_MOESM1_ESM.pdf]

A - CO_2 assimilation rate

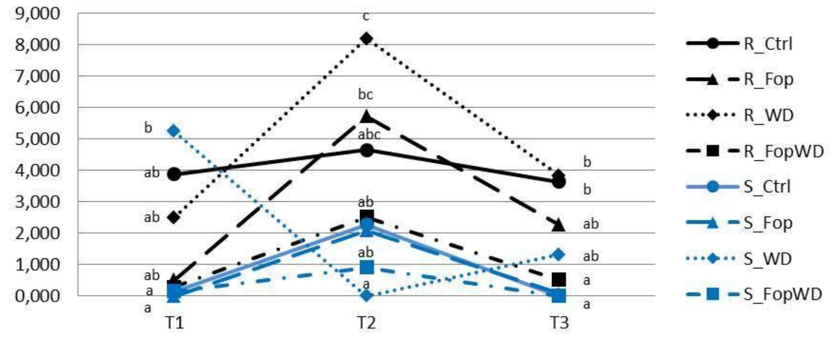

gs - stomatal conductance

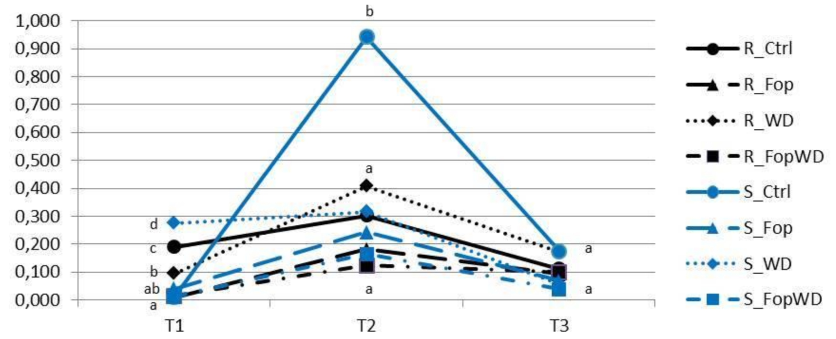

E - transpiration rate

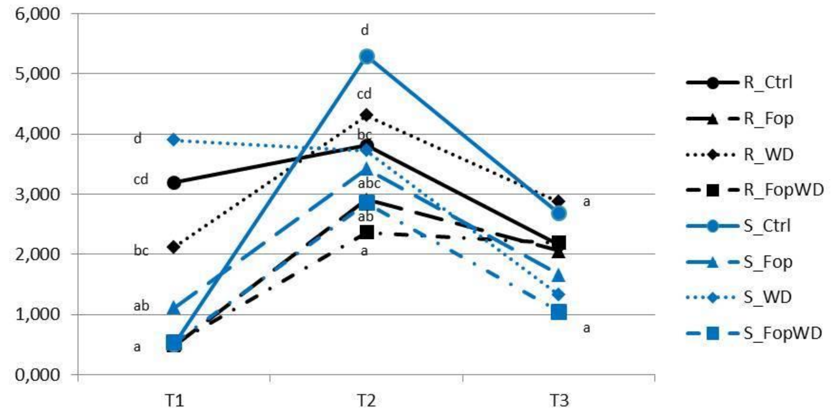

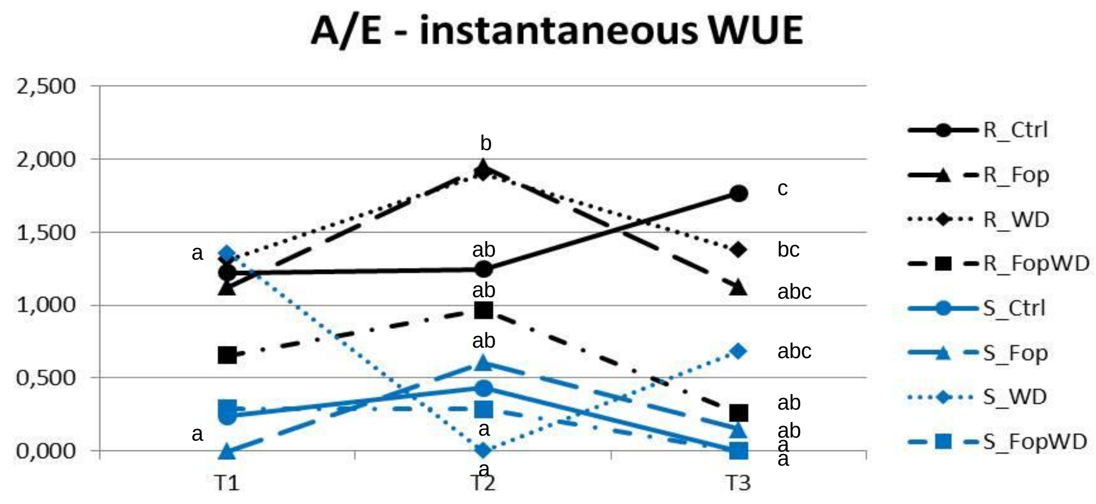
A/E - instantaneous WUE
2,500
2,000
1,500
1,000
0,500
0,000
T1
T2
T3
R_Ctrl
R_Fop
R_WD
R_FopWD
S_Ctrl
S_Fop
S_WD
S_FopWD

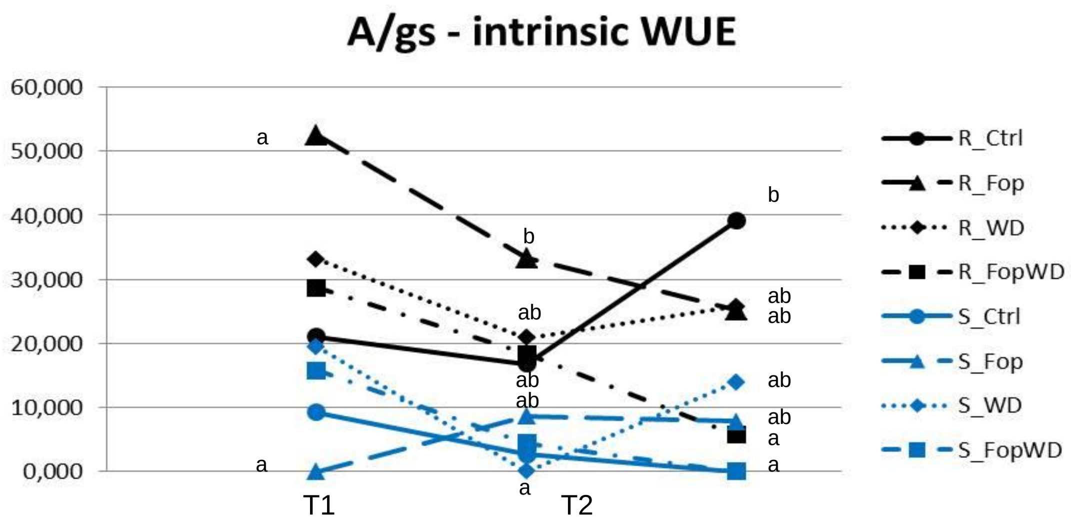
A/gs - intrinsic WUE
60,000
50,000
40,000
30,000
20,000
10,000
0,000
T1
T2
R_Ctrl
R_Fop
R_WD
R_FopWD
S_Ctrl
S_Fop
S_WD
S_FopWD

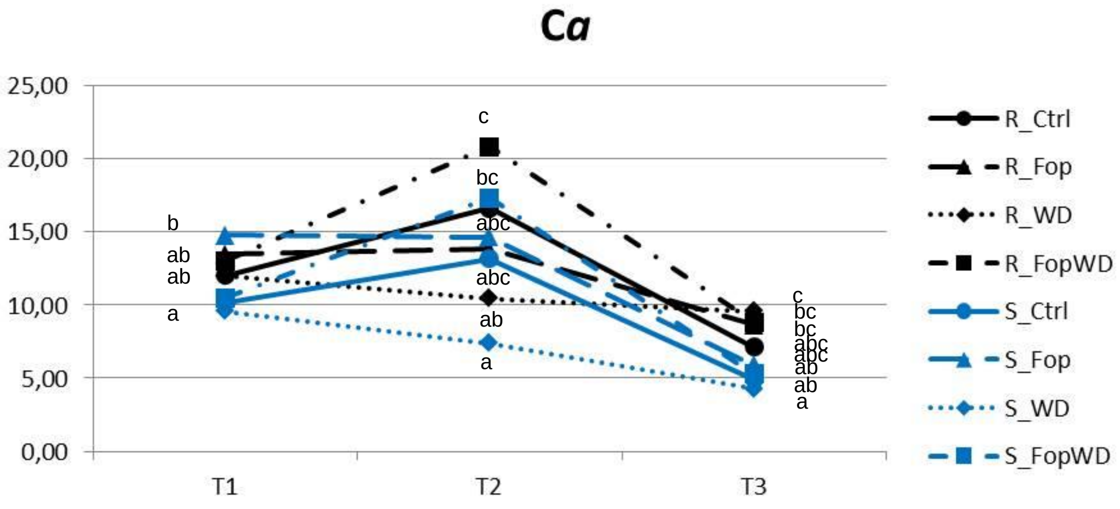
Ca
25,00
20,00
15,00
10,00
5,00
0,00
T1
T2
T3
R_Ctrl
R_Fop
R_WD
R_FopWD
S_Ctrl
S_Fop
S_WD
S_FopWD

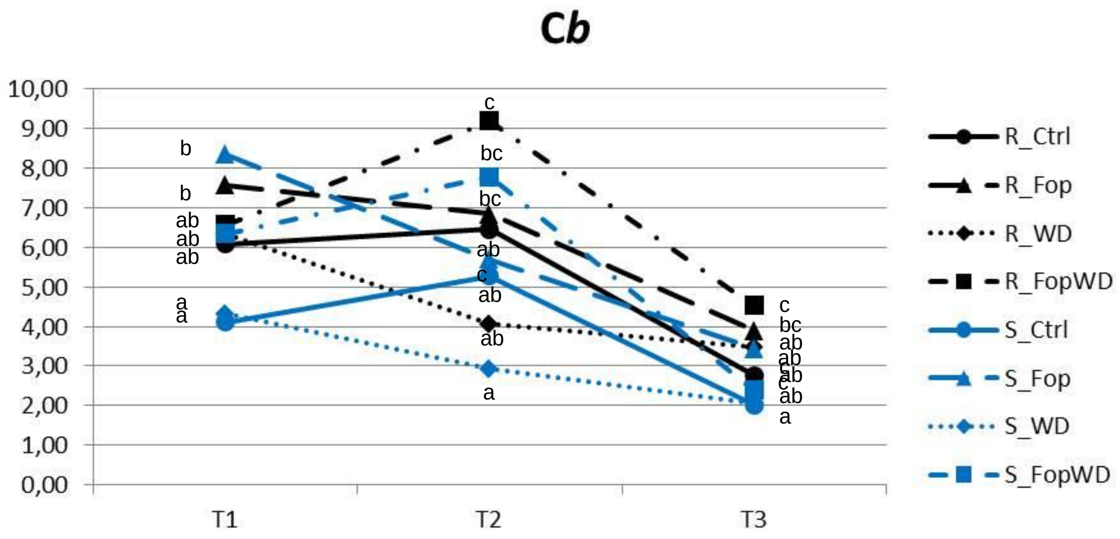
Cb
10,00
9,00
8,00
7,00
6,00
5,00
4,00
3,00
2,00
1,00
0,00
T1
T2
T3
R_Ctrl
R_Fop
R_WD
R_FopWD
S_Ctrl
S_Fop
S_WD
S_FopWD

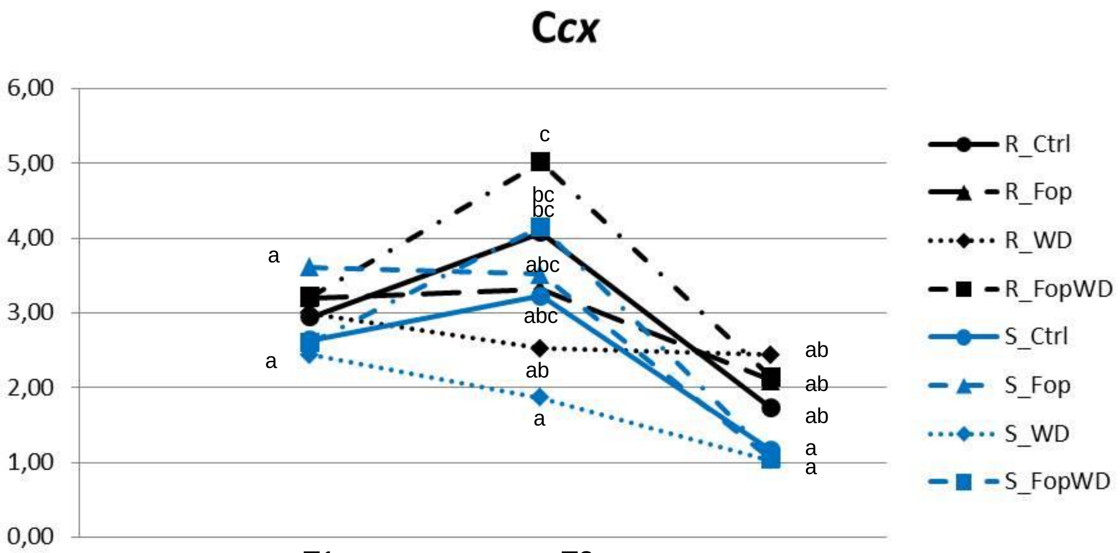
Ccx
6,00
5,00
4,00
3,00
2,00
1,00
0,00
R_Ctrl
R_Fop
R_WD
R_FopWD
S_Ctrl
S_Fop
S_WD
S_FopWD

T1 T2
T3

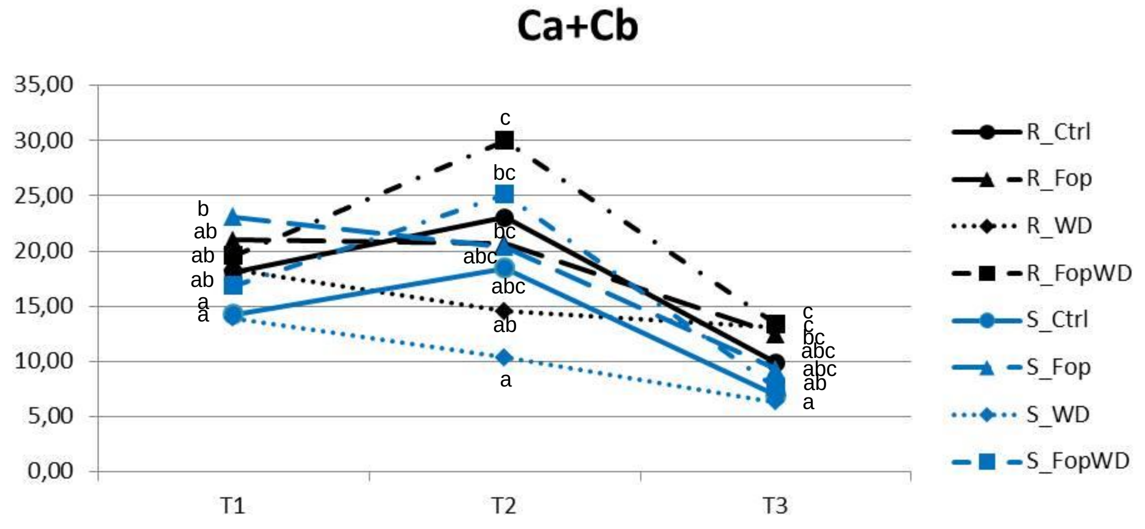
Ca+Cb
35,00
30,00
25,00
20,00
15,00
10,00
5,00
0,00
T1
T2
T3
R_Ctrl
R_Fop
R_WD
R_FopWD
S_Ctrl
S_Fop
S_WD
S_FopWD

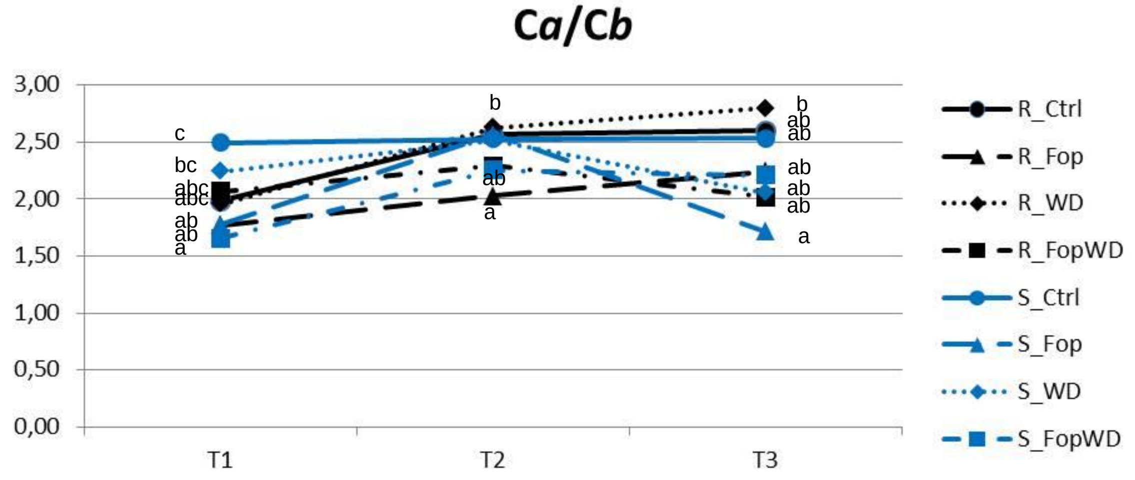
Ca/Cb
3,00
2,50
2,00
1,50
1,00
0,50
0,00
T1
T2
T3
R_Ctrl
R_Fop
R_WD
R_FopWD
S_Ctrl
S_Fop
S_WD
S_FopWD

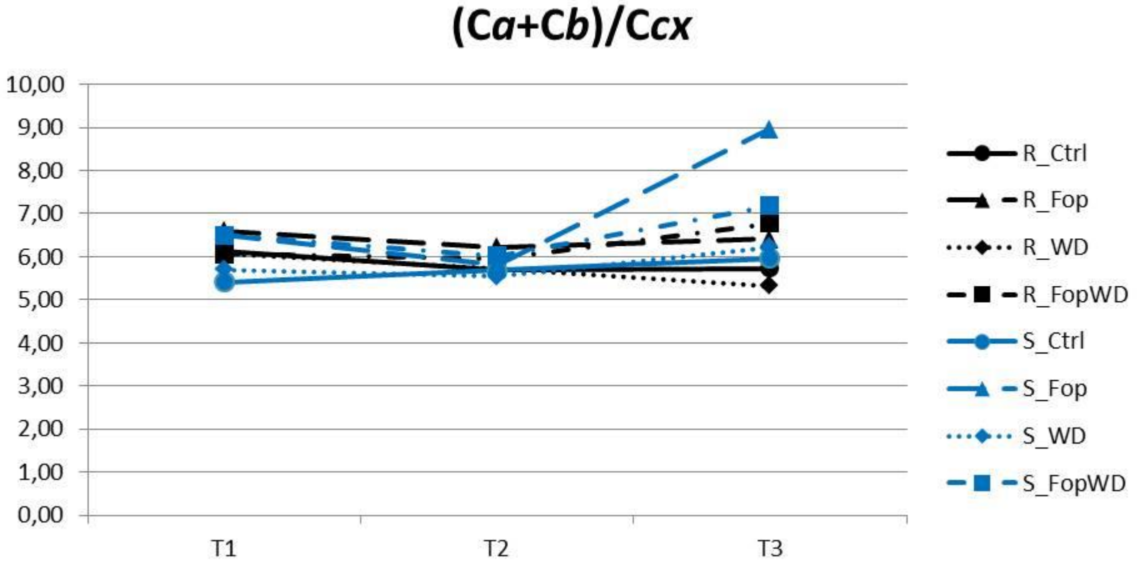
(Ca+Cb)/Ccx
10,00
9,00
8,00
7,00
6,00
5,00
4,00
3,00
2,00
1,00
0,00
T1
T2
T3
R_Ctrl
R_Fop
R_WD
R_FopWD
S_Ctrl
S_Fop
S_WD
S_FopWD

Figure S1: Photosynthesis-related traits variation in two contrasting common bean accessions (on resistant R and one susceptible S), evaluated at three time points (T1= 48 h, T2= 96 h and T3= 8days) after stress imposition. Fop = fusarium wilt caused by *Fusarium oxysporum* f. sp. *phaseoli* SP1- race 6, WD = water deficit (40 % field capacity), *Fop*WD = combined fusarium wilt + water deficit stress.
Different letters at each time point represent Tukey's test significant differences between conditions at 95 % confidence interval.

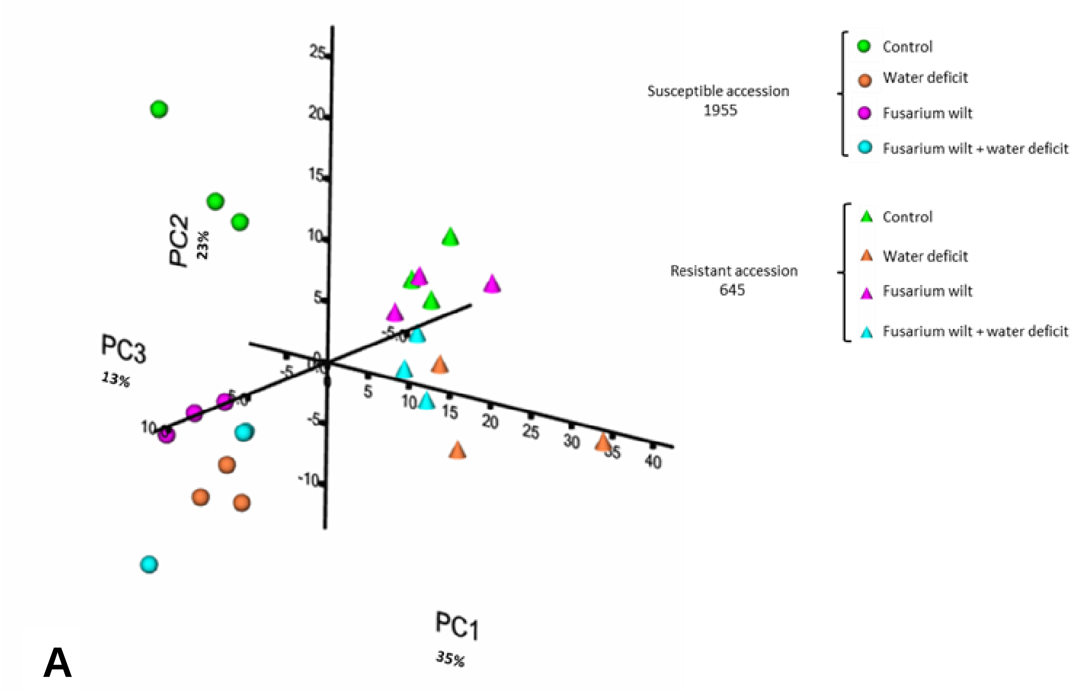

PC2
23%
PC3
13%
PC1
35%
Susceptible accession
1955
Control
Water deficit
Fusarium wilt
Fusarium wilt + water deficit
Resistant accession
645
Control
Water deficit
Fusarium wilt
Fusarium wilt + water deficit
A

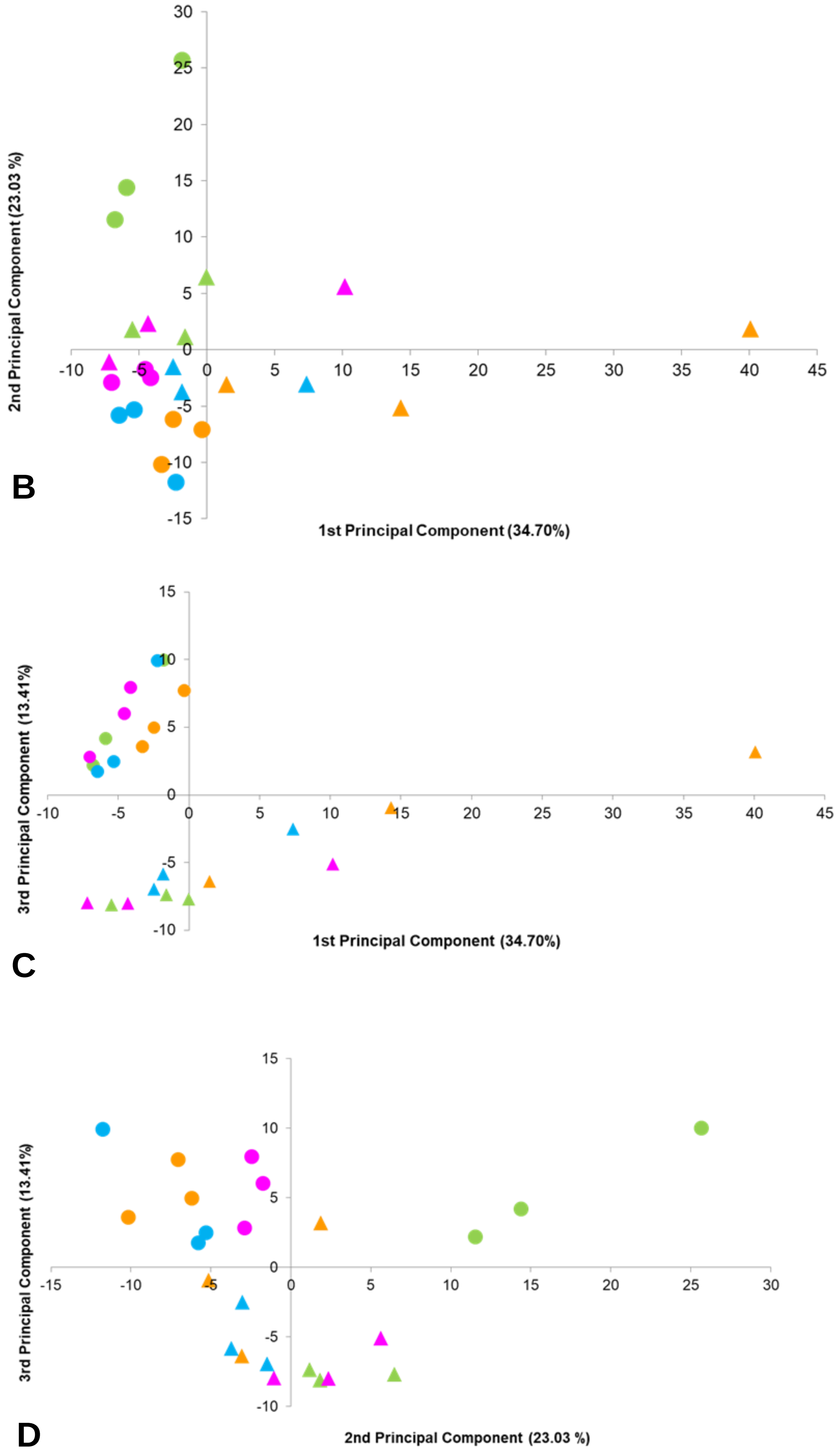

B
2nd Principal Component (23.03 %)
1st Principal Component (34.70%)
30
25
20
15
10
5
0
-5
-10
-15
-10
-5
0
5
10
15
20
25
30
35
40
45
C
3rd Principal Component (13.41%)
1st Principal Component (34.70%)
15
10
5
0
-5
-10
-10
-5
0
5
10
15
20
25
30
35
40
45
D
3rd Principal Component (13.41%)
2nd Principal Component (23.03 %)
15
10
5
0
-5
-10
-15
-10
-5
0
5
10
15
20
25
30

Figure S2: Principal component (PC) analysis of gene expression levels (read counts of 294 genes expressed in all conditions) in two common bean accessions (1955 susceptible, and 645 resistant) 96 h after exposure to water deficit (orange), fusarium wilt (pink), and to the combination of both stresses (blue). The control condition is depicted in green. The susceptible accession is represented by circles and the resistant accession by triangles. **A)** Together the first three PCs explained 71% of the total variance observed. **B)** PC1vsPC2 explained 58% of the total variation. PC1 mainly separates one sample of the resistant accession under water deficit (single stress) conditions. **C)** PC1vsPC3 explained 48% of the total variation. PC3 mainly separated the resistant from the susceptible samples. **D)** PC2vsPC3 explained 36% of the total variation. In this plot, the clustering of the samples from the same accession/condition is easily observed.

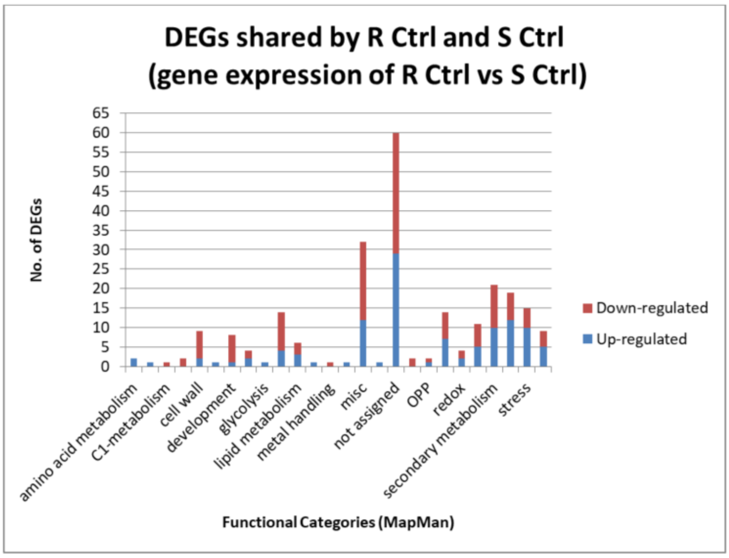
DEGs shared by R Ctrl and S Ctrl
(gene expression of R Ctrl vs S Ctrl)
No. of DEGs
65
60
55
50
45
40
35
30
25
20
15
10
5
0
amino acid metabolism
C1-metabolism
cell wall
development
glycolysis
lipid metabolism
metal handling
misc
not assigned
OPP
redox
secondary metabolism
stress
Functional Categories (MapMan)
Down-regulated
Up-regulated

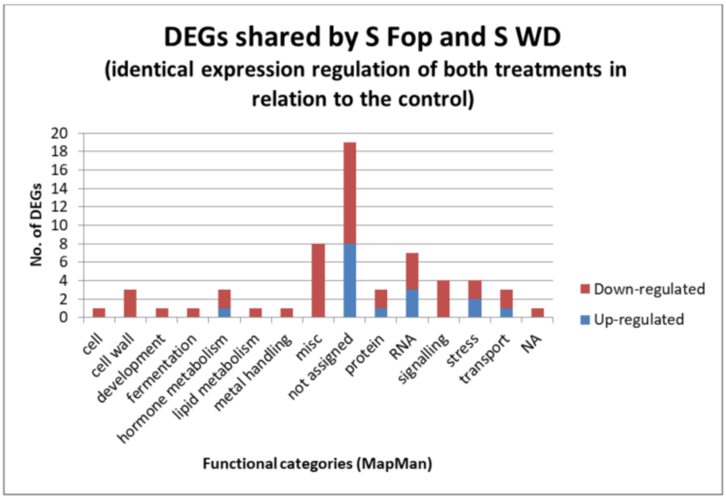
DEGs shared by S Fop and S WD
(identical expression regulation of both treatments in relation to the control)
No. of DEGs
20
18
16
14
12
10
8
6
4
2
0
cell
cell wall
development
fermentation
hormone metabolism
lipid metabolism
metal handling
misc
not assigned
protein
RNA
signalling
stress
transport
NA
Functional categories (MapMan)
Down-regulated
Up-regulated

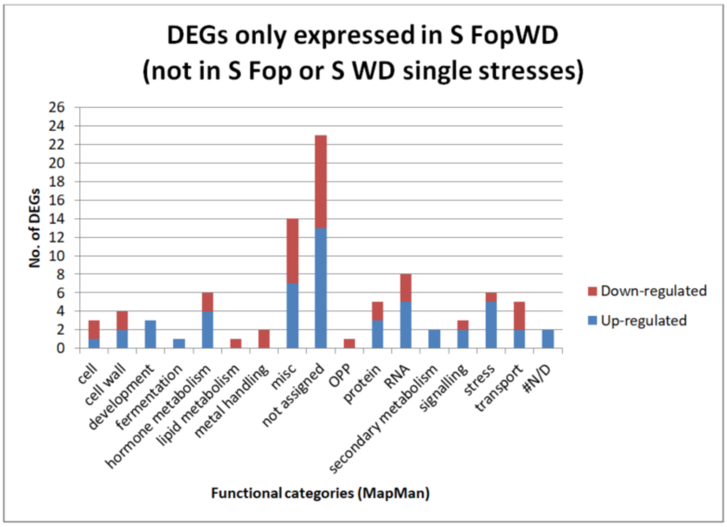
DEGs only expressed in S FopWD
(not in S Fop or S WD single stresses)
No. of DEGs
26
24
22
20
18
16
14
12
10
8
6
4
2
0
cell
cell wall
development
fermentation
hormone metabolism
lipid metabolism
metal handling
misc
not assigned
OPP
protein
RNA
secondary metabolism
signalling
stress
transport
#N/D
Functional categories (MapMan)
Down-regulated
Up-regulated

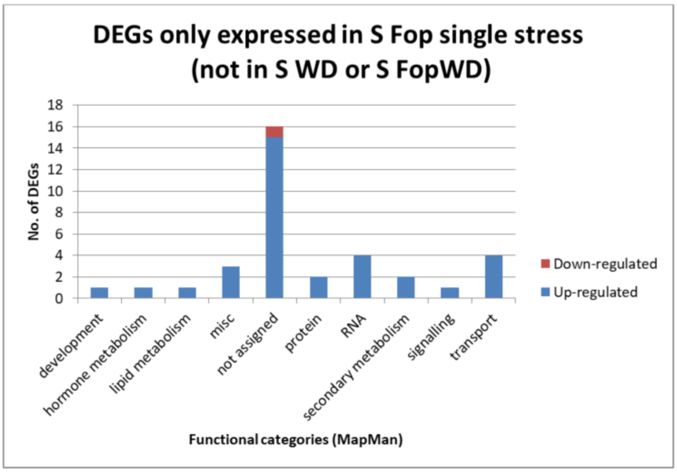
DEGs only expressed in S Fop single stress
(not in S WD or S FopWD)
No. of DEGs
Functional categories (MapMan)
development
hormone metabolism
lipid metabolism
misc
not assigned
protein
RNA
secondary metabolism
signalling
transport
Down-regulated
Up-regulated

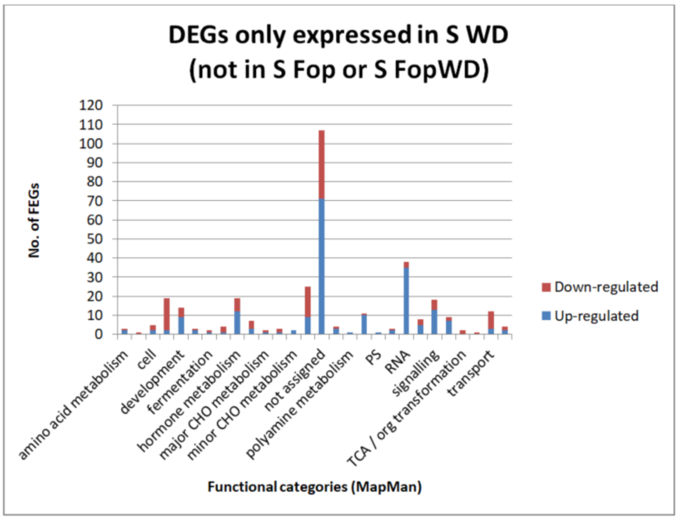
DEGs only expressed in S WD
(not in S Fop or S FopWD)
No. of FEGs
Functional categories (MapMan)
amino acid metabolism
cell
development
fermentation
hormone metabolism
major CHO metabolism
minor CHO metabolism
not assigned
polyamine metabolism
PS
RNA
signalling
TCA / org transformation
transport
Down-regulated
Up-regulated

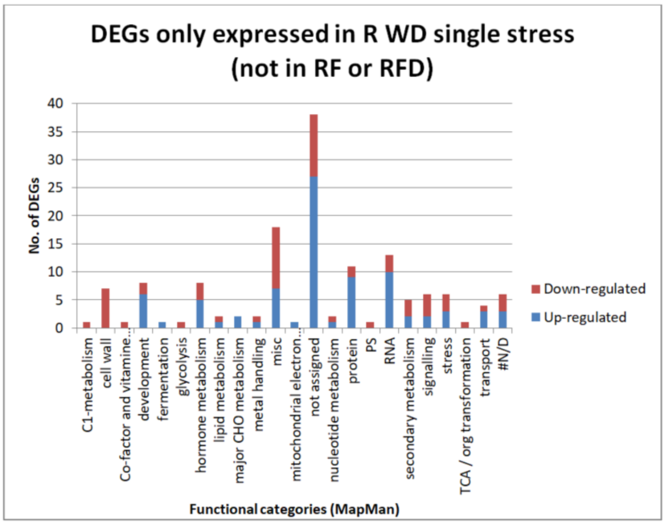
DEGs only expressed in R WD single stress
(not in RF or RFD)
No. of DEGs
Functional categories (MapMan)
C1-metabolism
cell wall
Co-factor and vitamine...
development
fermentation
glycolysis
hormone metabolism
lipid metabolism
major CHO metabolism
metal handling
misc
mitochondrial electron...
not assigned
nucleotide metabolism
protein
PS
RNA
secondary metabolism
signalling
stress
TCA / org transformation
transport
#N/D
Down-regulated
Up-regulated

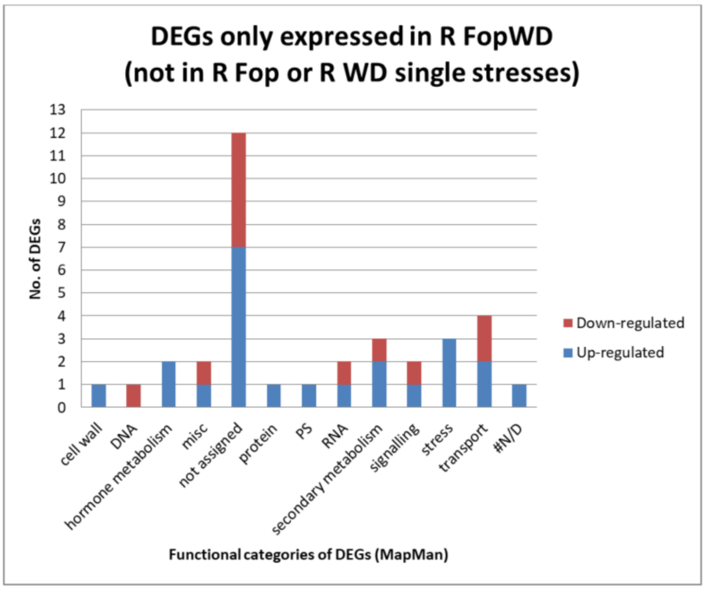

Figure S3: Number of differentially expressed genes (DEGs) in the different treatments for the two accessions, with the indication of the up and the down-regulated genes, in relation to control conditions. The functional categories were assigned to the genes using Mercator and MapMan web tools. R = resistant accession, S = susceptible accession, Ctrl = control, WD = water deficit single stress, *Fop* = fusarium wilt single stress, *Fop*WD = combined fusarium wilt + water

Figure S4: Percentage of differentially expressed genes (DEGs), in relation to control conditions, from each functional category for the two accessions under the different treatments. DEGs functional categories retrieved from Mercator and MapMan web tools. Functional categories of DEGs for the conditions **A)** R WD, **B)** R FopWD, **C)** S WD, **D)** S Fop, **E)** S FopWD.
R = resistant accession (645), S = susceptible accession (1955), WD = water deficit single stress, *Fop* = fusarium wilt single stress, *Fop*WD = combined fusarium wilt + water deficit stress.

R WD

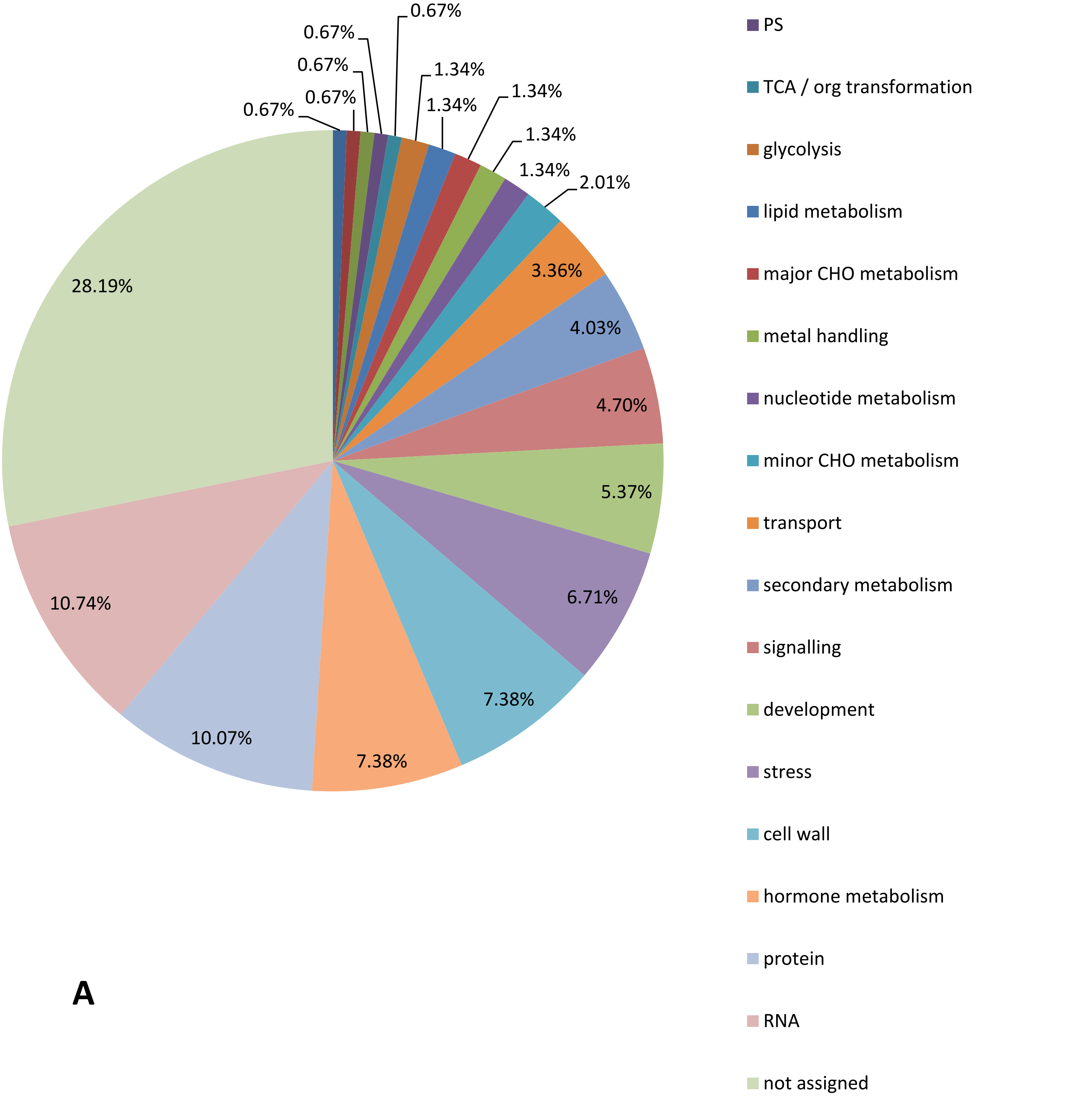

0.67%
0.67%
0.67%
0.67%
0.67%
1.34%
1.34%
1.34%
1.34%
1.34%
2.01%
3.36%
4.03%
4.70%
5.37%
6.71%
7.38%
7.38%
10.07%
10.74%
28.19%
C1-metabolism
Co-factor and vitamine metabolism
mitochondrial electron transport / ATP synthesis
PS
TCA / org transformation
glycolysis
lipid metabolism
major CHO metabolism
metal handling
nucleotide metabolism
minor CHO metabolism
transport
secondary metabolism
signalling
development
stress
cell wall
hormone metabolism
protein
RNA
not assigned

A

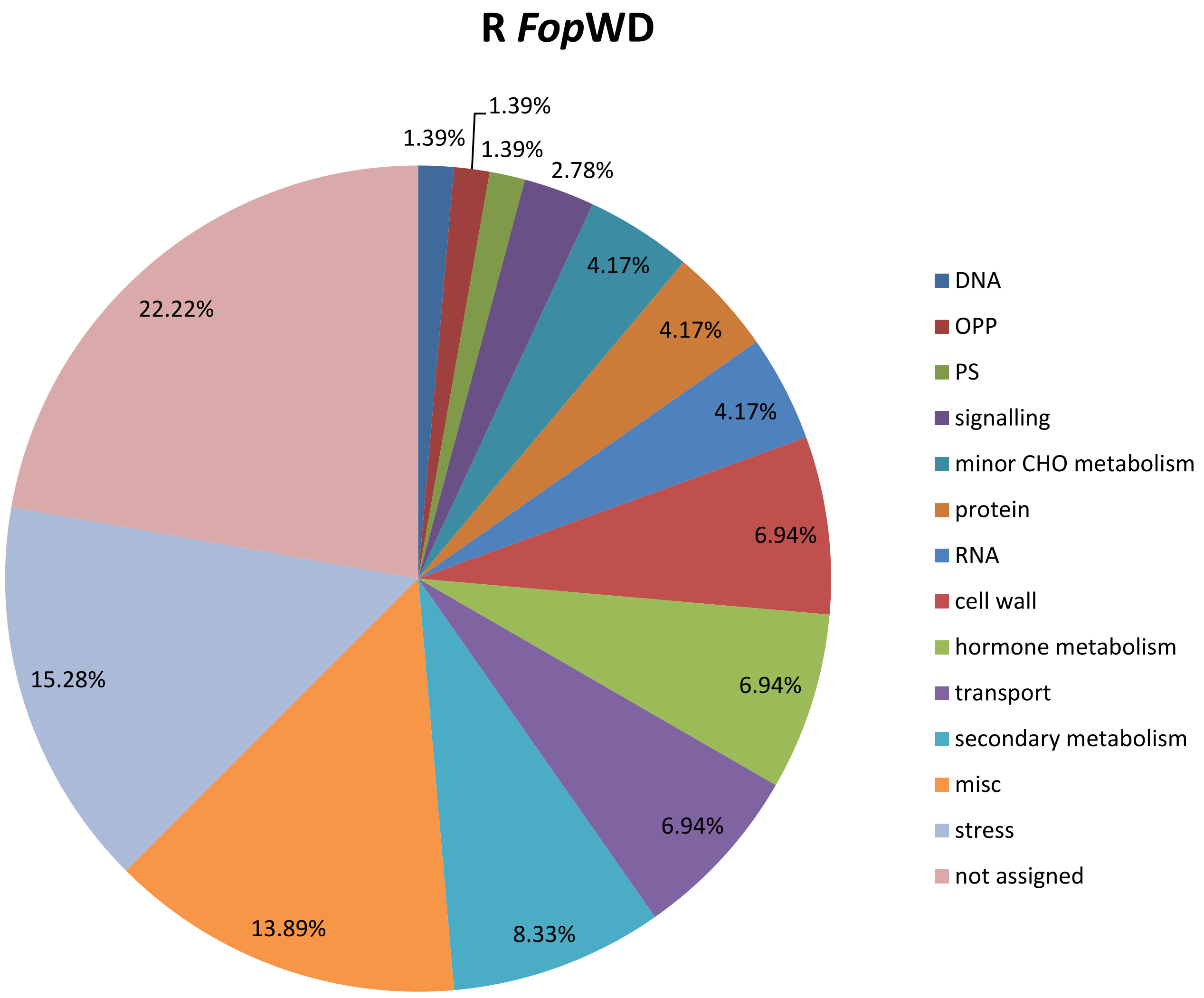
R *Fop*WD
1.39%
1.39%
1.39%
2.78%
4.17%
4.17%
4.17%
6.94%
6.94%
6.94%
8.33%
13.89%
15.28%
22.22%
DNA
OPP
PS
signalling
minor CHO metabolism
protein
RNA
cell wall
hormone metabolism
transport
secondary metabolism
misc
stress
not assigned

B

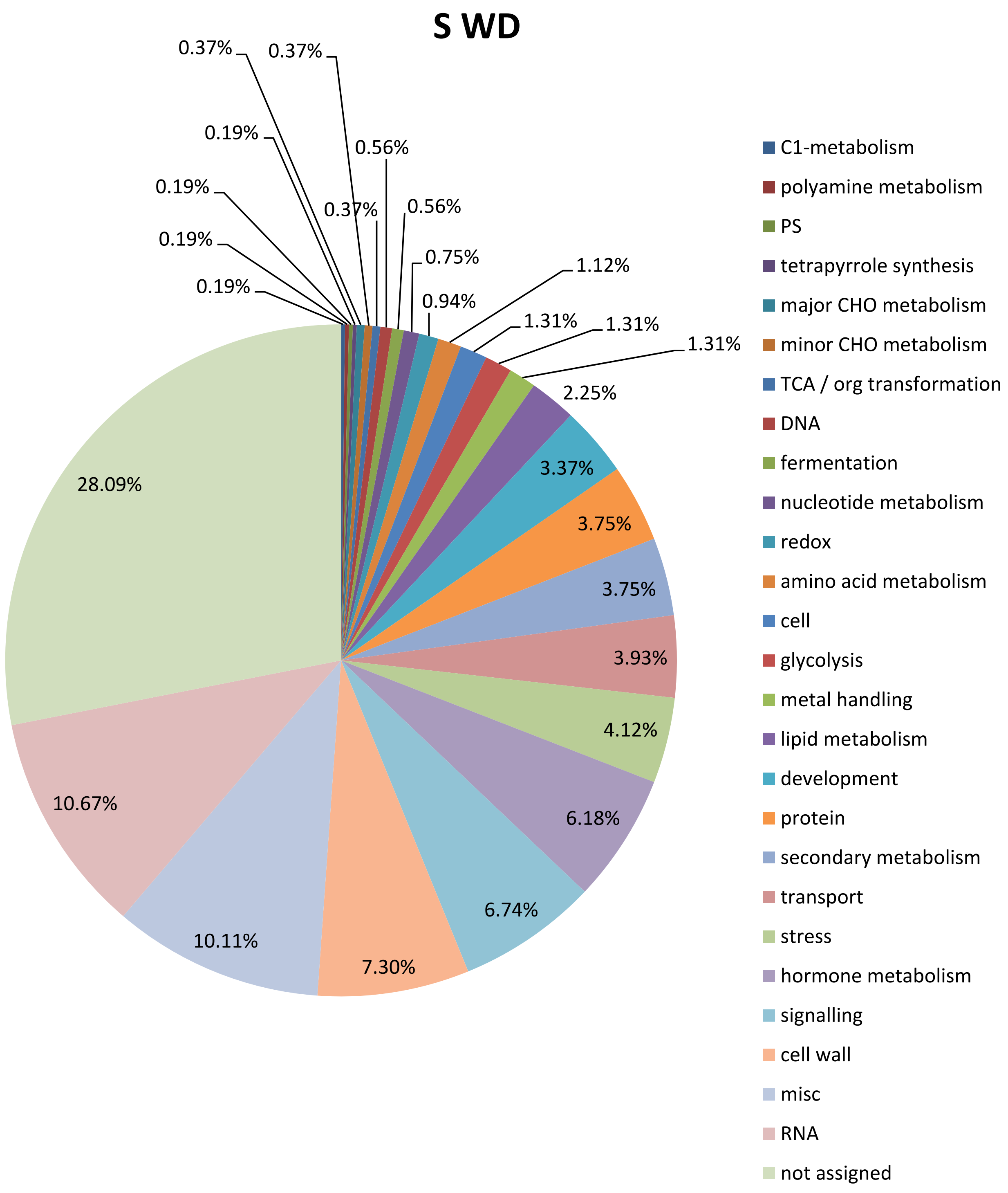
S WD
0.37%
0.37%
0.19%
0.56%
0.19%
0.37%
0.56%
0.19%
0.75%
1.12%
0.19%
0.94%
1.31%
1.31%
1.31%
2.25%
28.09%
3.37%
3.75%
3.75%
3.93%
4.12%
10.67%
6.18%
6.74%
10.11%
7.30%
C1-metabolism
polyamine metabolism
PS
tetrapyrrole synthesis
major CHO metabolism
minor CHO metabolism
TCA / org transformation
DNA
fermentation
nucleotide metabolism
redox
amino acid metabolism
cell
glycolysis
metal handling
lipid metabolism
development
protein
secondary metabolism
transport
stress
hormone metabolism
signalling
cell wall
misc
RNA
not assigned

C

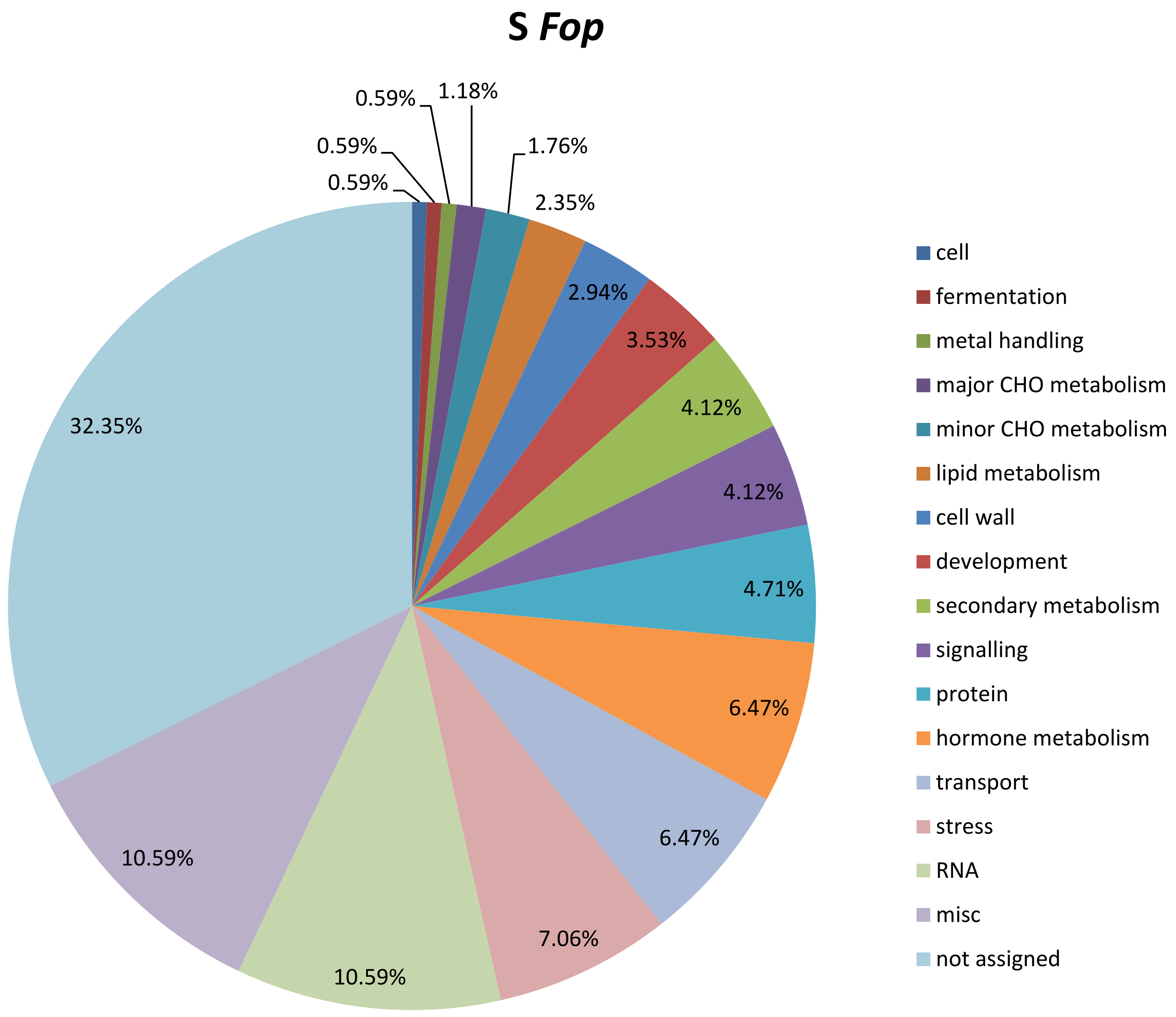
S Fop
0.59%
0.59%
0.59%
1.18%
1.76%
2.35%
2.94%
3.53%
4.12%
4.12%
4.71%
6.47%
6.47%
7.06%
10.59%
10.59%
32.35%
cell
fermentation
metal handling
major CHO metabolism
minor CHO metabolism
lipid metabolism
cell wall
development
secondary metabolism
signalling
protein
hormone metabolism
transport
stress
RNA
misc
not assigned

D

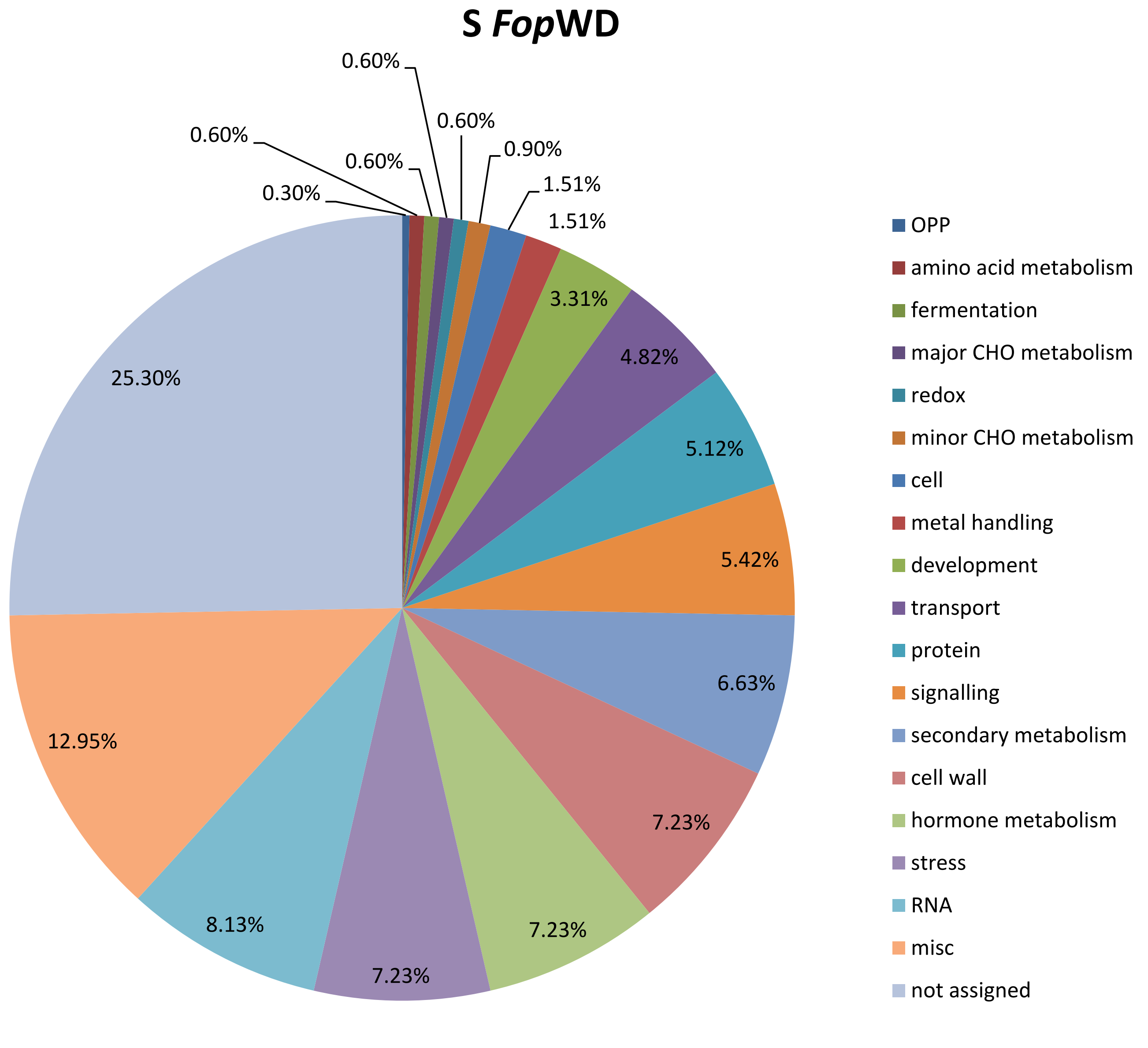
S *Fop*WD
0.60%
0.60%
0.90%
1.51%
1.51%
0.60%
0.60%
0.30%
3.31%
4.82%
5.12%
5.42%
6.63%
7.23%
7.23%
7.23%
8.13%
12.95%
25.30%
OPP
amino acid metabolism
fermentation
major CHO metabolism
redox
minor CHO metabolism
cell
metal handling
development
transport
protein
signalling
secondary metabolism
cell wall
hormone metabolism
stress
RNA
misc
not assigned

E

Figure S5: Heat maps and hierarchical clustering of the differentially expressed genes in the resistant (R-645) and susceptible (S-1955) common bean accessions. In each figure one functional category is represented. WD = water deficit single stress, Fop = fusarium wilt single stress, FopWD = combined fusarium wilt + water deficit stress. The colour scale represents the log2 fold change in relation to control conditions. Heat maps performed with Morpheus software available at https://software.broadinstitute.org/Morpheus

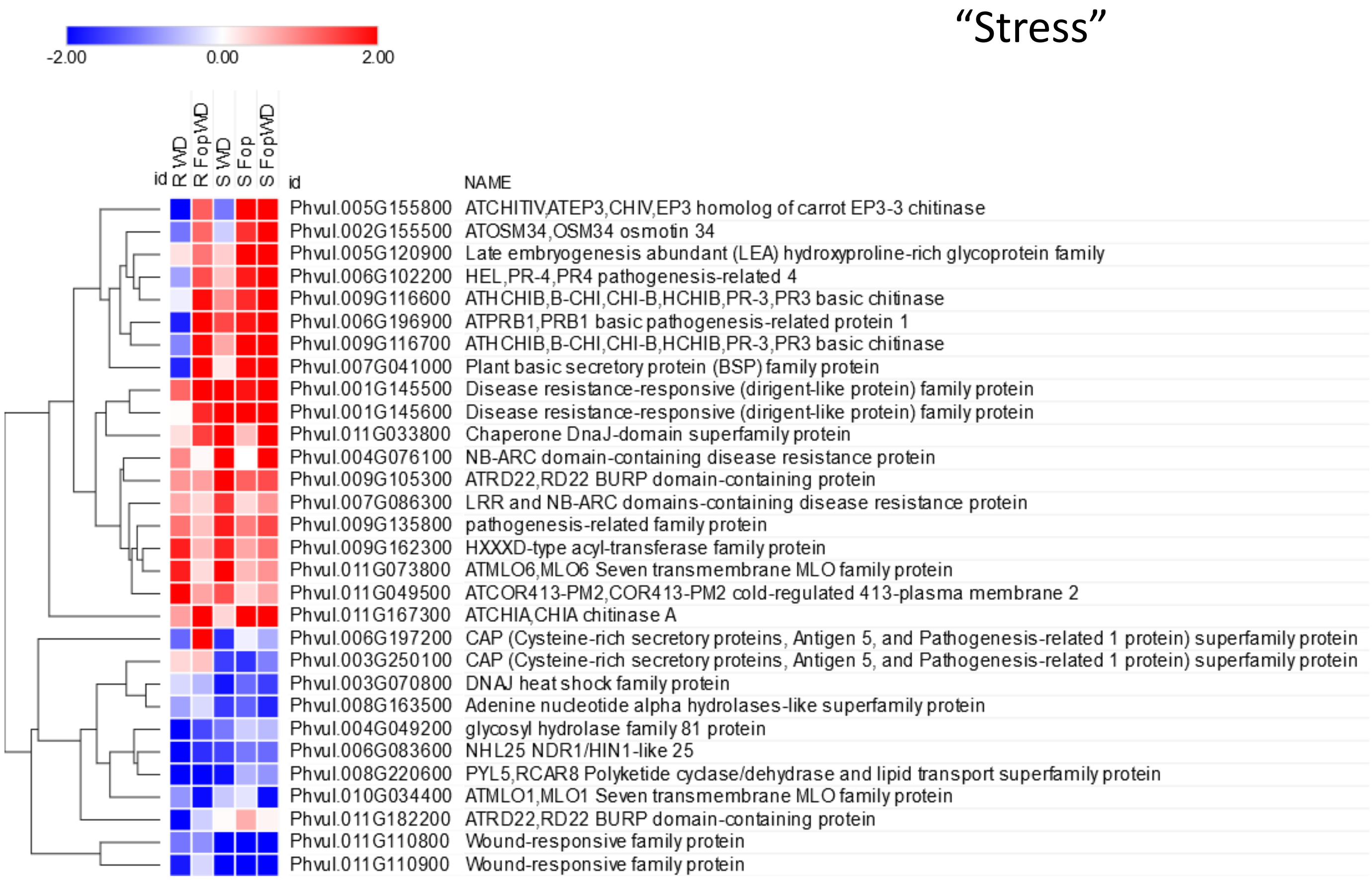

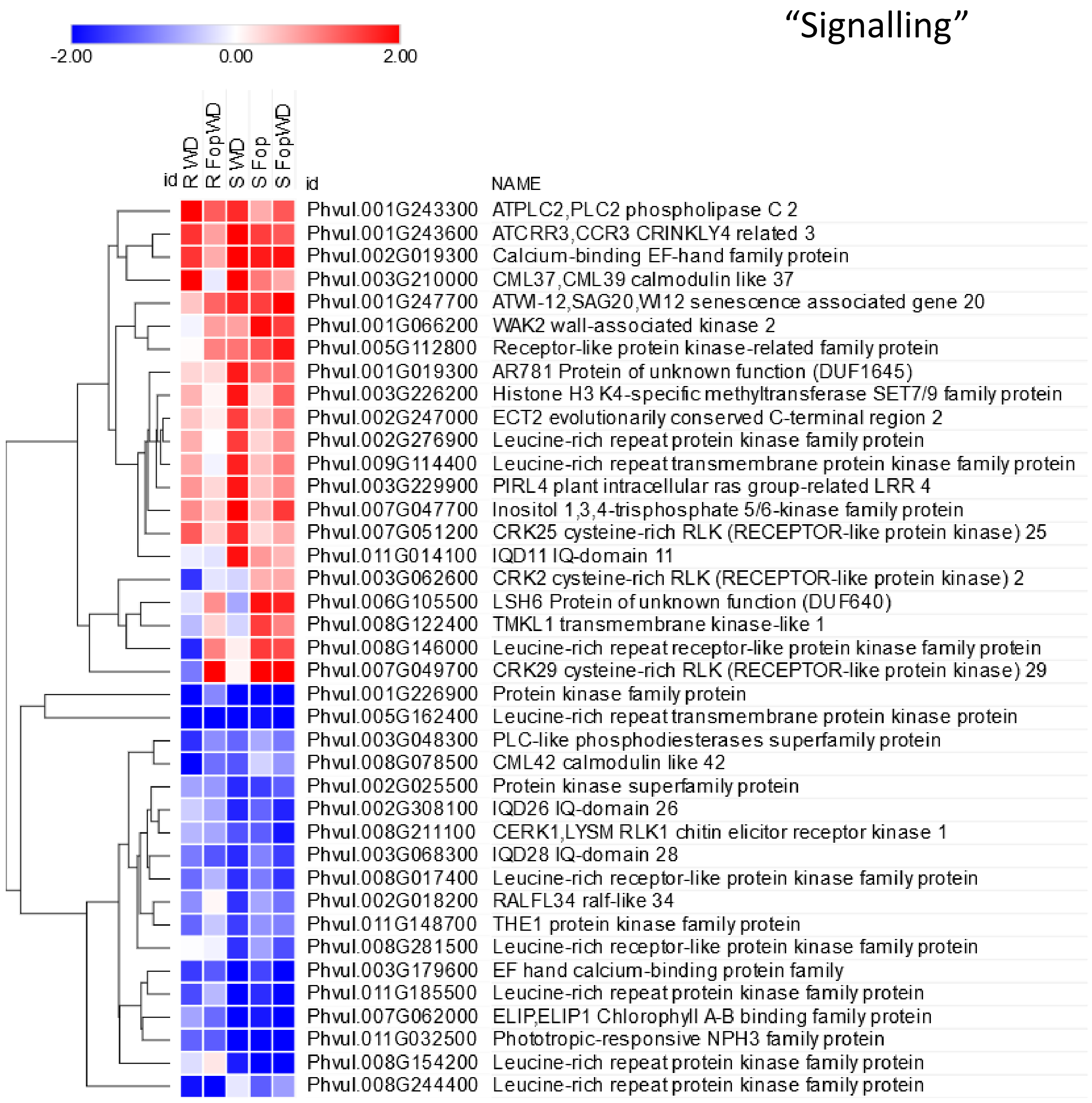
"Signalling"
-2.00
0.00
2.00
id
R WD
R FopWD
S WD
S Fop
S FopWD
id
NAME
Phvul.001G243300 ATPLC2,PLC2 phospholipase C 2
Phvul.001G243600 ATCRR3,CCR3 CRINKLY4 related 3
Phvul.002G019300 Calcium-binding EF-hand family protein
Phvul.003G210000 CML37,CML39 calmodulin like 37
Phvul.001G247700 ATWI-12,SAG20,WI12 senescence associated gene 20
Phvul.001G066200 WAK2 wall-associated kinase 2
Phvul.005G112800 Receptor-like protein kinase-related family protein
Phvul.001G019300 AR781 Protein of unknown function (DUF1645)
Phvul.003G226200 Histone H3 K4-specific methyltransferase SET7/9 family protein
Phvul.002G247000 ECT2 evolutionarily conserved C-terminal region 2
Phvul.002G276900 Leucine-rich repeat protein kinase family protein
Phvul.009G114400 Leucine-rich repeat transmembrane protein kinase family protein
Phvul.003G229900 PIRL4 plant intracellular ras group-related LRR 4
Phvul.007G047700 Inositol 1,3,4-trisphosphate 5/6-kinase family protein
Phvul.007G051200 CRK25 cysteine-rich RLK (RECEPTOR-like protein kinase) 25
Phvul.011G014100 IQD11 IQ-domain 11
Phvul.003G062600 CRK2 cysteine-rich RLK (RECEPTOR-like protein kinase) 2
Phvul.006G105500 LSH6 Protein of unknown function (DUF640)
Phvul.008G122400 TMKL1 transmembrane kinase-like 1
Phvul.008G146000 Leucine-rich repeat receptor-like protein kinase family protein
Phvul.007G049700 CRK29 cysteine-rich RLK (RECEPTOR-like protein kinase) 29
Phvul.001G226900 Protein kinase family protein
Phvul.005G162400 Leucine-rich repeat transmembrane protein kinase protein
Phvul.003G048300 PLC-like phosphodiesterases superfamily protein
Phvul.008G078500 CML42 calmodulin like 42
Phvul.002G025500 Protein kinase superfamily protein
Phvul.002G308100 IQD26 IQ-domain 26
Phvul.008G211100 CERK1,LYSM RLK1 chitin elicitor receptor kinase 1
Phvul.003G068300 IQD28 IQ-domain 28
Phvul.008G017400 Leucine-rich receptor-like protein kinase family protein
Phvul.002G018200 RALFL34 ralf-like 34
Phvul.011G148700 THE1 protein kinase family protein
Phvul.008G281500 Leucine-rich receptor-like protein kinase family protein
Phvul.003G179600 EF hand calcium-binding protein family
Phvul.011G185500 Leucine-rich repeat protein kinase family protein
Phvul.007G062000 ELIP,ELIP1 Chlorophyll A-B binding family protein
Phvul.011G032500 Phototropic-responsive NPH3 family protein
Phvul.008G154200 Leucine-rich repeat protein kinase family protein
Phvul.008G244400 Leucine-rich repeat protein kinase family protein

“Secondary metabolism”

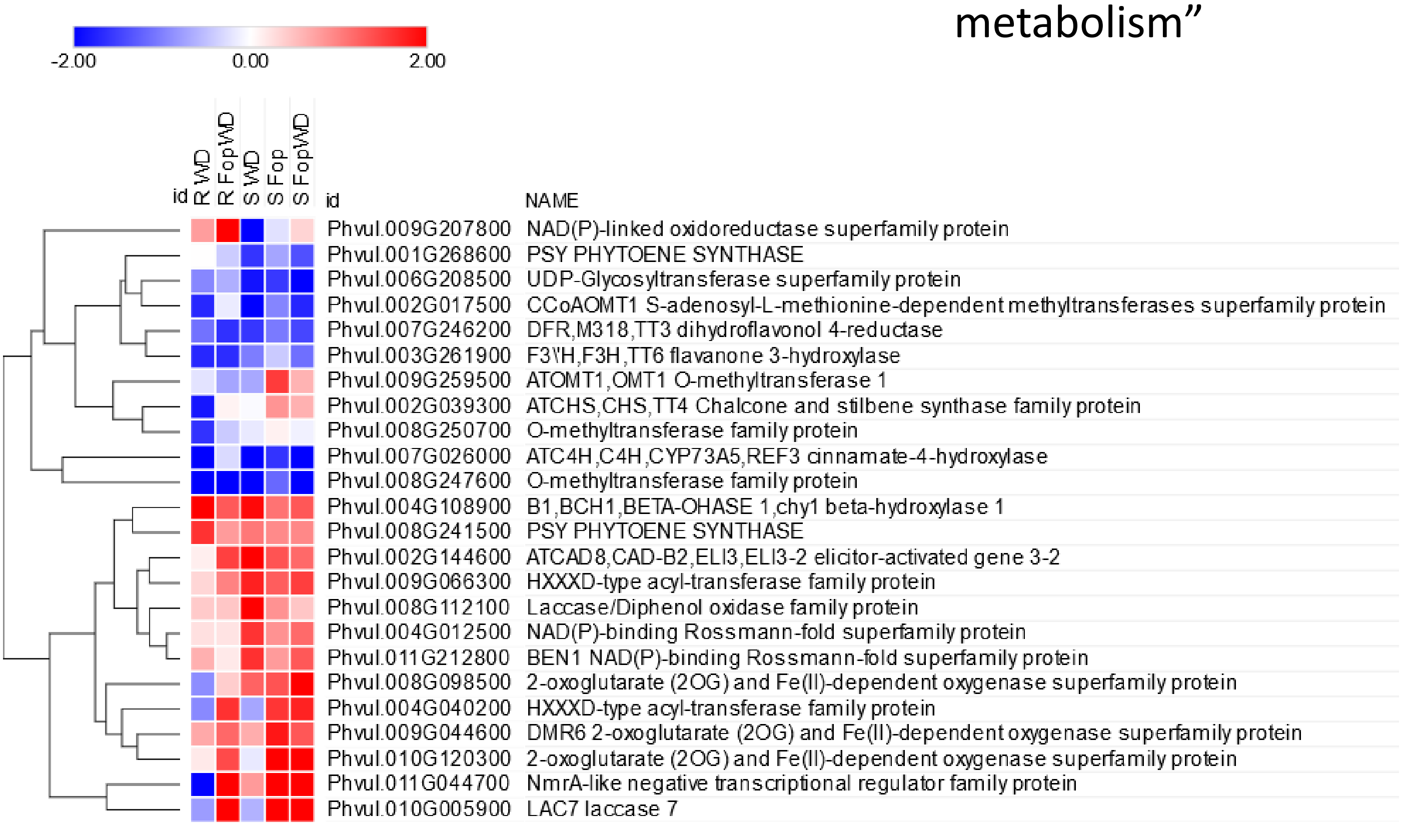

“Hormone metabolism”

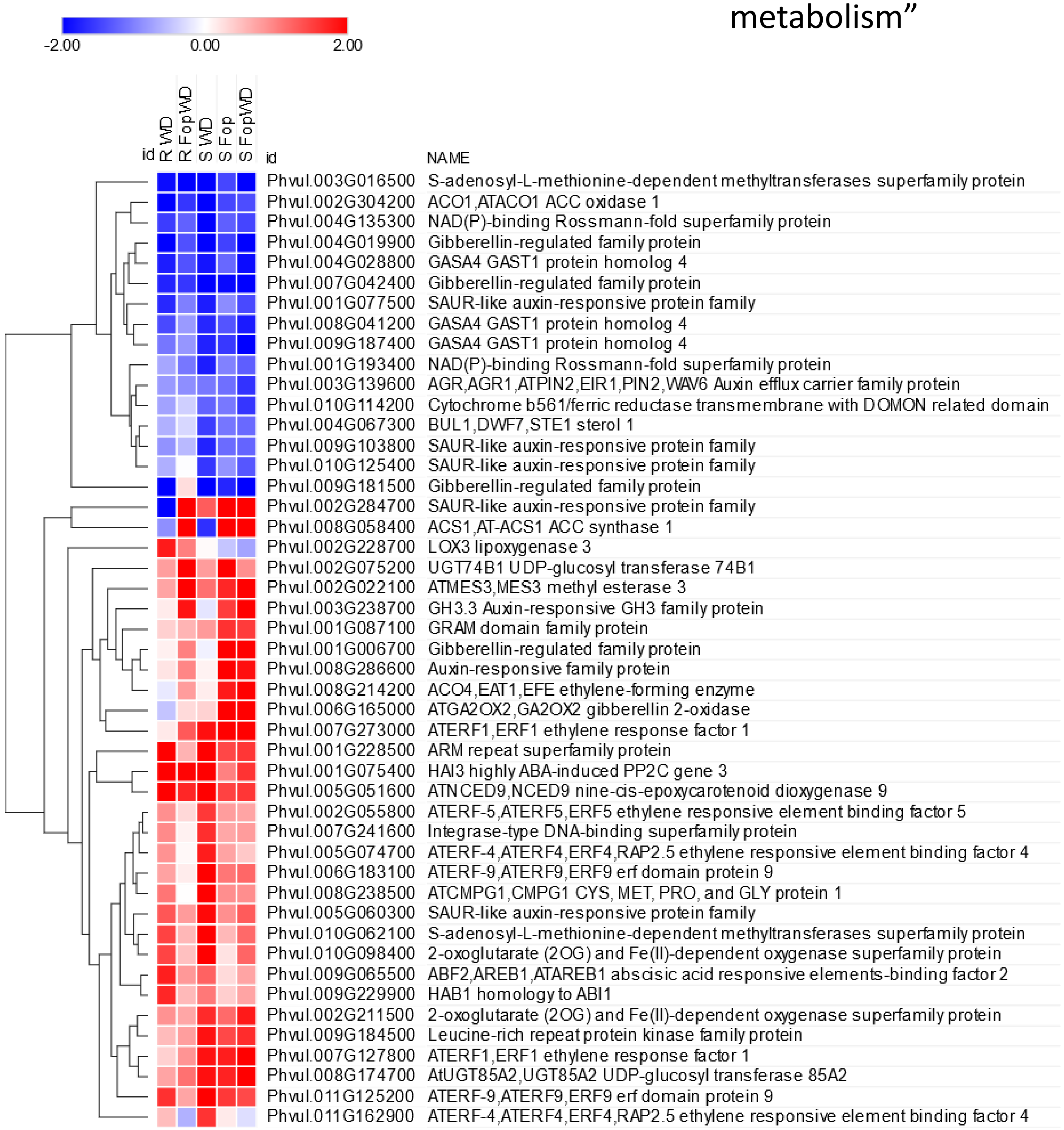

“Transport”

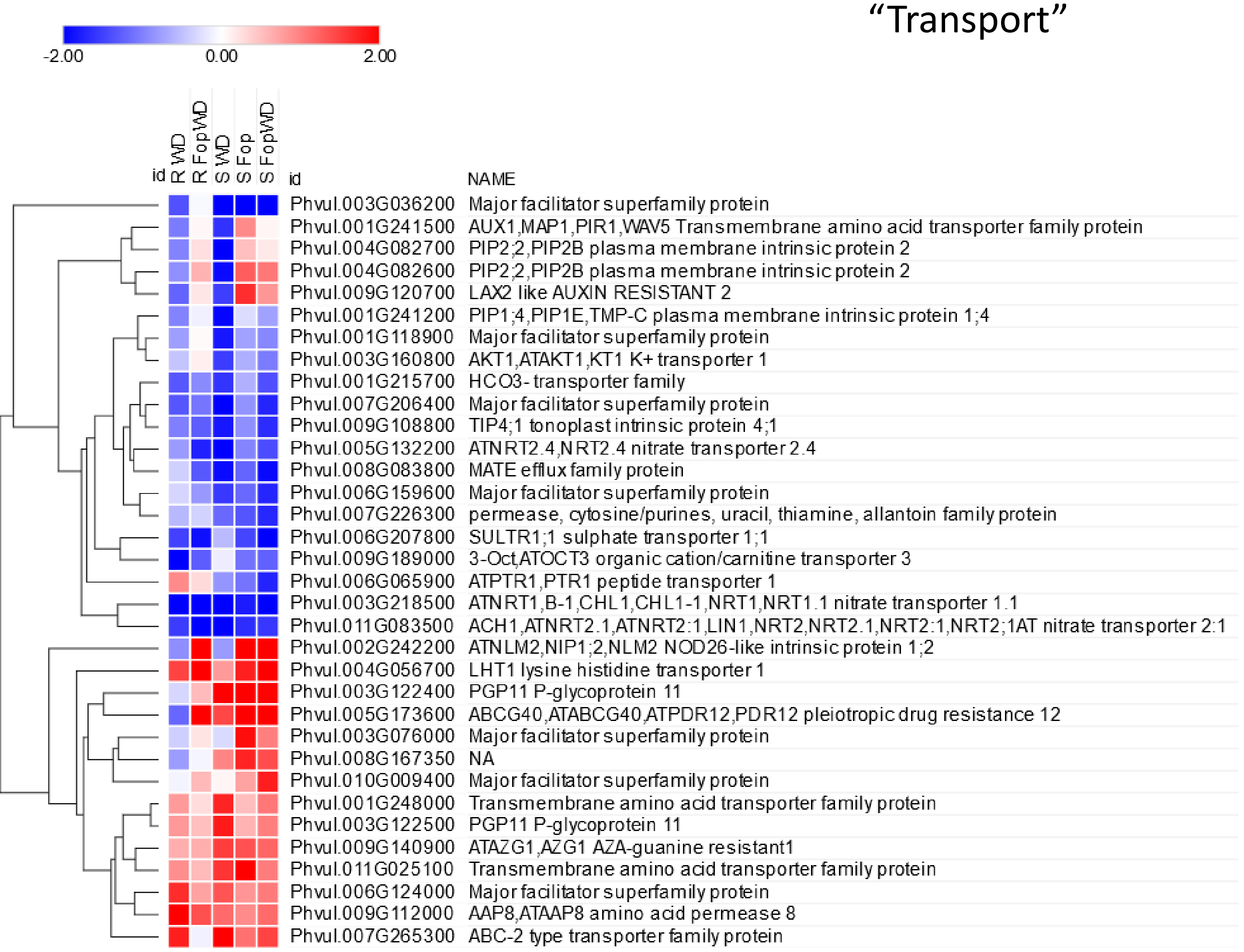

-2.00
0.00
2.00
id
R WD
R FopWD
S WD
S Fop
S FopWD
id
NAME
Phvul.003G036200 Major facilitator superfamily protein
Phvul.001G241500 AUX1,MAP1,PIR1,WAV5 Transmembrane amino acid transporter family protein
Phvul.004G082700 PIP2;2,PIP2B plasma membrane intrinsic protein 2
Phvul.004G082600 PIP2;2,PIP2B plasma membrane intrinsic protein 2
Phvul.009G120700 LAX2 like AUXIN RESISTANT 2
Phvul.001G241200 PIP1;4,PIP1E,TMP-C plasma membrane intrinsic protein 1;4
Phvul.001G118900 Major facilitator superfamily protein
Phvul.003G160800 AKT1,ATAKT1,KT1 K+ transporter 1
Phvul.001G215700 HCO3- transporter family
Phvul.007G206400 Major facilitator superfamily protein
Phvul.009G108800 TIP4;1 tonoplast intrinsic protein 4;1
Phvul.005G132200 ATNRT2.4,NRT2.4 nitrate transporter 2.4
Phvul.008G083800 MATE efflux family protein
Phvul.006G159600 Major facilitator superfamily protein
Phvul.007G226300 permease, cytosine/purines, uracil, thiamine, allantoin family protein
Phvul.006G207800 SULTR1;1 sulphate transporter 1;1
Phvul.009G189000 3-Oct,ATOCT3 organic cation/carnitine transporter 3
Phvul.006G065900 ATPTR1,PTR1 peptide transporter 1
Phvul.003G218500 ATNRT1,B-1,CHL1,CHL1-1,NRT1,NRT1.1 nitrate transporter 1.1
Phvul.011G083500 ACH1,ATNRT2.1,ATNRT2:1,LIN1,NRT2,NRT2.1,NRT2:1,NRT2;1AT nitrate transporter 2:1
Phvul.002G242200 ATNLM2,NIP1;2,NLM2 NOD26-like intrinsic protein 1;2
Phvul.004G056700 LHT1 lysine histidine transporter 1
Phvul.003G122400 PGP11 P-glycoprotein 11
Phvul.005G173600 ABCG40,ATABCG40,ATPDR12,PDR12 pleiotropic drug resistance 12
Phvul.003G076000 Major facilitator superfamily protein
Phvul.008G167350 NA
Phvul.010G009400 Major facilitator superfamily protein
Phvul.001G248000 Transmembrane amino acid transporter family protein
Phvul.003G122500 PGP11 P-glycoprotein 11
Phvul.009G140900 ATAZG1,AZG1 AZA-guanine resistant1
Phvul.011G025100 Transmembrane amino acid transporter family protein
Phvul.006G124000 Major facilitator superfamily protein
Phvul.009G112000 AAP8,ATAAP8 amino acid permease 8
Phvul.007G265300 ABC-2 type transporter family protein

“RNA”

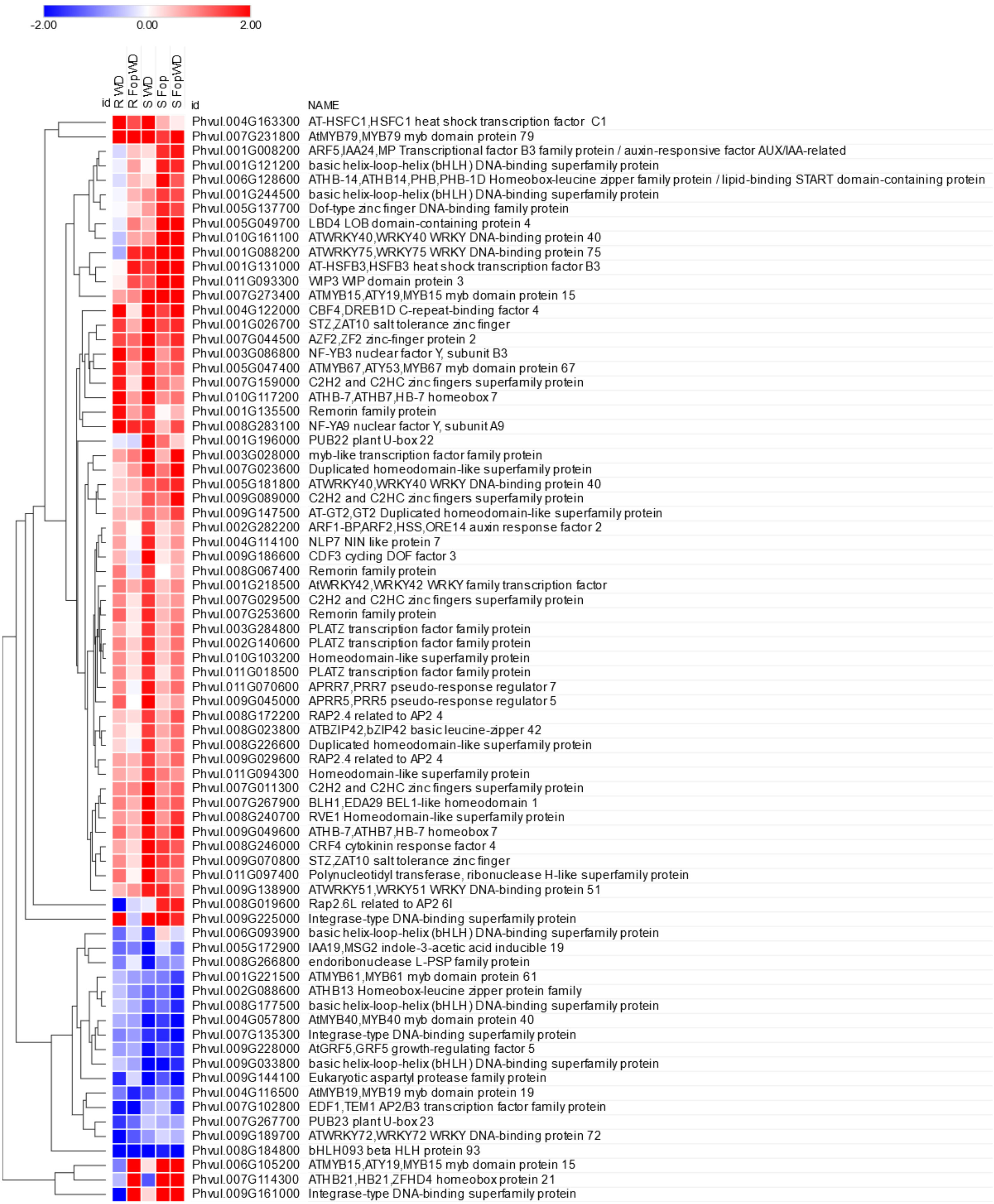

-2.00
0.00
2.00
id
R WD
R FopWD
S WD
S Fop
S FopWD
id
NAME
Phvul.004G163300 AT-HSFC1,HSFC1 heat shock transcription factor C1
Phvul.007G231800 AtMYB79,MYB79 myb domain protein 79
Phvul.001G008200 ARF5,IAA24,MP Transcriptional factor B3 family protein / auxin-responsive factor AUX/IAA-related
Phvul.001G121200 basic helix-loop-helix (bHLH) DNA-binding superfamily protein
Phvul.006G128600 ATHB-14,ATHB14,PHB,PHB-1D Homeobox-leucine zipper family protein / lipid-binding START domain-containing protein
Phvul.001G244500 basic helix-loop-helix (bHLH) DNA-binding superfamily protein
Phvul.005G137700 Dof-type zinc finger DNA-binding family protein
Phvul.005G049700 LBD4 LOB domain-containing protein 4
Phvul.010G161100 ATWRKY40,WRKY40 WRKY DNA-binding protein 40
Phvul.001G088200 ATWRKY75,WRKY75 WRKY DNA-binding protein 75
Phvul.001G131000 AT-HSFB3,HSFB3 heat shock transcription factor B3
Phvul.011G093300 WIP3 WIP domain protein 3
Phvul.007G273400 ATMYB15,ATY19,MYB15 myb domain protein 15
Phvul.004G122000 CBF4,DREB1D C-repeat-binding factor 4
Phvul.001G026700 STZ,ZAT10 salt tolerance zinc finger
Phvul.007G044500 AZF2,ZF2 zinc-finger protein 2
Phvul.003G086800 NF-YB3 nuclear factor Y, subunit B3
Phvul.005G047400 ATMYB67,ATY53,MYB67 myb domain protein 67
Phvul.007G159000 C2H2 and C2HC zinc fingers superfamily protein
Phvul.010G117200 ATHB-7,ATHB7,HB-7 homeobox 7
Phvul.001G135500 Remorin family protein
Phvul.008G283100 NF-YA9 nuclear factor Y, subunit A9
Phvul.001G196000 PUB22 plant U-box 22
Phvul.003G028000 myb-like transcription factor family protein
Phvul.007G023600 Duplicated homeodomain-like superfamily protein
Phvul.005G181800 ATWRKY40,WRKY40 WRKY DNA-binding protein 40
Phvul.009G089000 C2H2 and C2HC zinc fingers superfamily protein
Phvul.009G147500 AT-GT2,GT2 Duplicated homeodomain-like superfamily protein
Phvul.002G282200 ARF1-BP,ARF2,HSS,ORE14 auxin response factor 2
Phvul.004G114100 NLP7 NIN like protein 7
Phvul.009G186600 CDF3 cycling DOF factor 3
Phvul.008G067400 Remorin family protein
Phvul.001G218500 AtWRKY42,WRKY42 WRKY family transcription factor
Phvul.007G029500 C2H2 and C2HC zinc fingers superfamily protein
Phvul.007G253600 Remorin family protein
Phvul.003G284800 PLATZ transcription factor family protein
Phvul.002G140600 PLATZ transcription factor family protein
Phvul.010G103200 Homeodomain-like superfamily protein
Phvul.011G018500 PLATZ transcription factor family protein
Phvul.011G070600 APRR7,PRR7 pseudo-response regulator 7
Phvul.009G045000 APRR5,PRR5 pseudo-response regulator 5
Phvul.008G172200 RAP2.4 related to AP2 4
Phvul.008G023800 ATBZIP42,bZIP42 basic leucine-zipper 42
Phvul.008G226600 Duplicated homeodomain-like superfamily protein
Phvul.009G029600 RAP2.4 related to AP2 4
Phvul.011G094300 Homeodomain-like superfamily protein
Phvul.007G011300 C2H2 and C2HC zinc fingers superfamily protein
Phvul.007G267900 BLH1,EDA29 BEL1-like homeodomain 1
Phvul.008G240700 RVE1 Homeodomain-like superfamily protein
Phvul.009G049600 ATHB-7,ATHB7,HB-7 homeobox 7
Phvul.008G246000 CRF4 cytokinin response factor 4
Phvul.009G070800 STZ,ZAT10 salt tolerance zinc finger
Phvul.011G097400 Polynucleotidyl transferase, ribonuclease H-like superfamily protein
Phvul.009G138900 ATWRKY51,WRKY51 WRKY DNA-binding protein 51
Phvul.008G019600 Rap2.6L related to AP2 6l
Phvul.009G225000 Integrase-type DNA-binding superfamily protein
Phvul.006G093900 basic helix-loop-helix (bHLH) DNA-binding superfamily protein
Phvul.005G172900 IAA19,MSG2 indole-3-acetic acid inducible 19
Phvul.008G266800 endoribonuclease L-PSP family protein
Phvul.001G221500 ATMYB61,MYB61 myb domain protein 61
Phvul.002G088600 ATHB13 Homeobox-leucine zipper protein family
Phvul.008G177500 basic helix-loop-helix (bHLH) DNA-binding superfamily protein
Phvul.004G057800 AtMYB40,MYB40 myb domain protein 40
Phvul.007G135300 Integrase-type DNA-binding superfamily protein
Phvul.009G228000 AtGRF5,GRF5 growth-regulating factor 5
Phvul.009G033800 basic helix-loop-helix (bHLH) DNA-binding superfamily protein
Phvul.009G144100 Eukaryotic aspartyl protease family protein
Phvul.004G116500 AtMYB19,MYB19 myb domain protein 19
Phvul.007G102800 EDF1,TEM1 AP2/B3 transcription factor family protein
Phvul.007G267700 PUB23 plant U-box 23
Phvul.009G189700 ATWRKY72,WRKY72 WRKY DNA-binding protein 72
Phvul.008G184800 bHLH093 beta HLH protein 93
Phvul.006G105200 ATMYB15,ATY19,MYB15 myb domain protein 15
Phvul.007G114300 ATHB21,HB21,ZFHD4 homeobox protein 21
Phvul.009G161000 Integrase-type DNA-binding superfamily protein

"Protein"

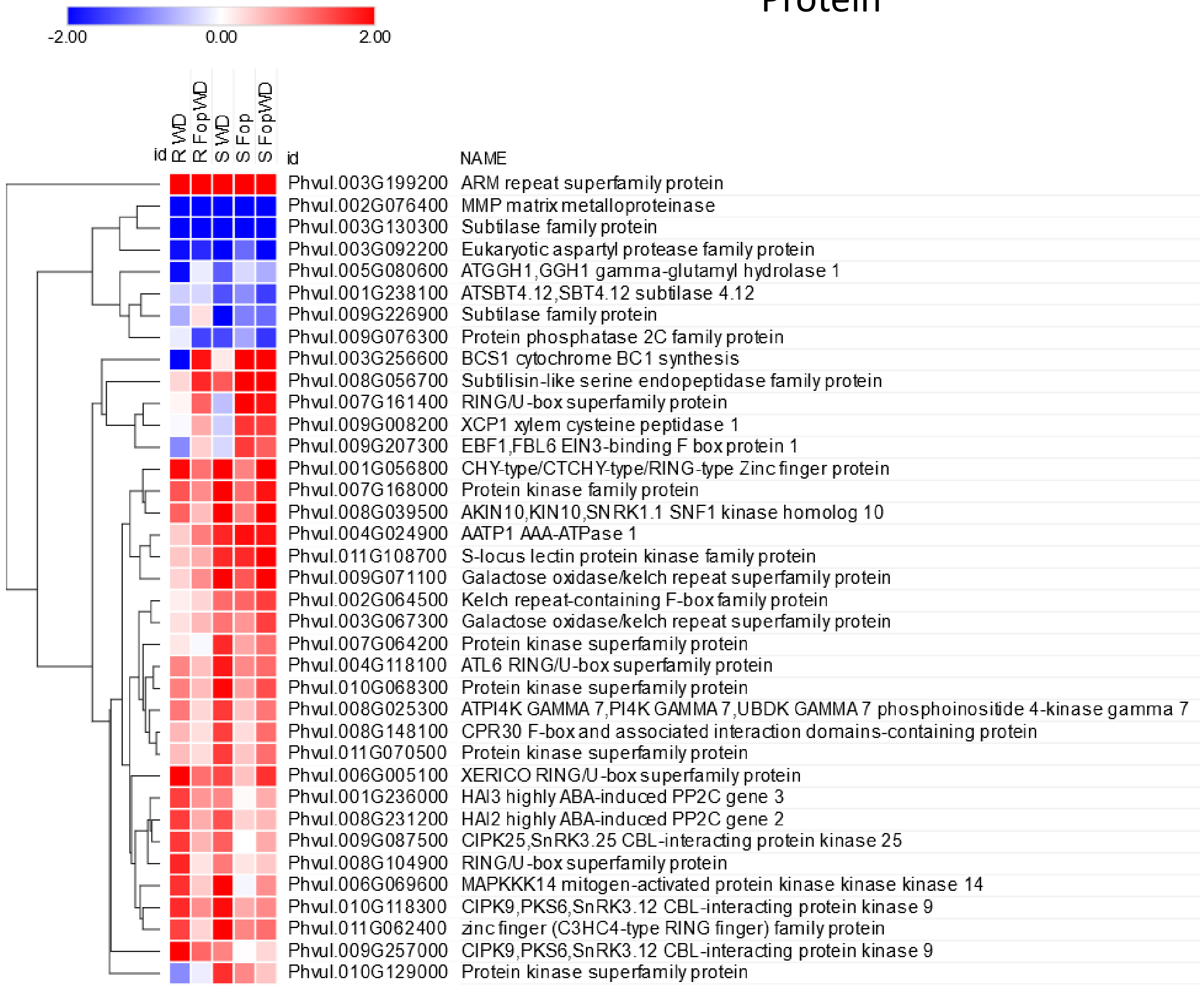

-2.00
0.00
2.00
id
R WD
R FopWD
S WD
S Fop
S FopWD
id
NAME
Phvul.003G199200 ARM repeat superfamily protein
Phvul.002G076400 MMP matrix metalloproteinase
Phvul.003G130300 Subtilase family protein
Phvul.003G092200 Eukaryotic aspartyl protease family protein
Phvul.005G080600 ATGGH1,GGH1 gamma-glutamyl hydrolase 1
Phvul.001G238100 ATSBT4.12,SBT4.12 subtilase 4.12
Phvul.009G226900 Subtilase family protein
Phvul.009G076300 Protein phosphatase 2C family protein
Phvul.003G256600 BCS1 cytochrome BC1 synthesis
Phvul.008G056700 Subtilisin-like serine endopeptidase family protein
Phvul.007G161400 RING/U-box superfamily protein
Phvul.009G008200 XCP1 xylem cysteine peptidase 1
Phvul.009G207300 EBF1,FBL6 EIN3-binding F box protein 1
Phvul.001G056800 CHY-type/CTCHY-type/RING-type Zinc finger protein
Phvul.007G168000 Protein kinase family protein
Phvul.008G039500 AKIN10,KIN10,SNRK1.1 SNF1 kinase homolog 10
Phvul.004G024900 AATP1 AAA-ATPase 1
Phvul.011G108700 S-locus lectin protein kinase family protein
Phvul.009G071100 Galactose oxidase/kelch repeat superfamily protein
Phvul.002G064500 Kelch repeat-containing F-box family protein
Phvul.003G067300 Galactose oxidase/kelch repeat superfamily protein
Phvul.007G064200 Protein kinase superfamily protein
Phvul.004G118100 ATL6 RING/U-box superfamily protein
Phvul.010G068300 Protein kinase superfamily protein
Phvul.008G025300 ATPI4K GAMMA 7,PI4K GAMMA 7,UBDK GAMMA 7 phosphoinositide 4-kinase gamma 7
Phvul.008G148100 CPR30 F-box and associated interaction domains-containing protein
Phvul.011G070500 Protein kinase superfamily protein
Phvul.006G005100 XERICO RING/U-box superfamily protein
Phvul.001G236000 HAI3 highly ABA-induced PP2C gene 3
Phvul.008G231200 HAI2 highly ABA-induced PP2C gene 2
Phvul.009G087500 CIPK25,SnRK3.25 CBL-interacting protein kinase 25
Phvul.008G104900 RING/U-box superfamily protein
Phvul.006G069600 MAPKKK14 mitogen-activated protein kinase kinase kinase 14
Phvul.010G118300 CIPK9,PKS6,SnRK3.12 CBL-interacting protein kinase 9
Phvul.011G062400 zinc finger (C3HC4-type RING finger) family protein
Phvul.009G257000 CIPK9,PKS6,SnRK3.12 CBL-interacting protein kinase 9
Phvul.010G129000 Protein kinase superfamily protein

“Cell wall”

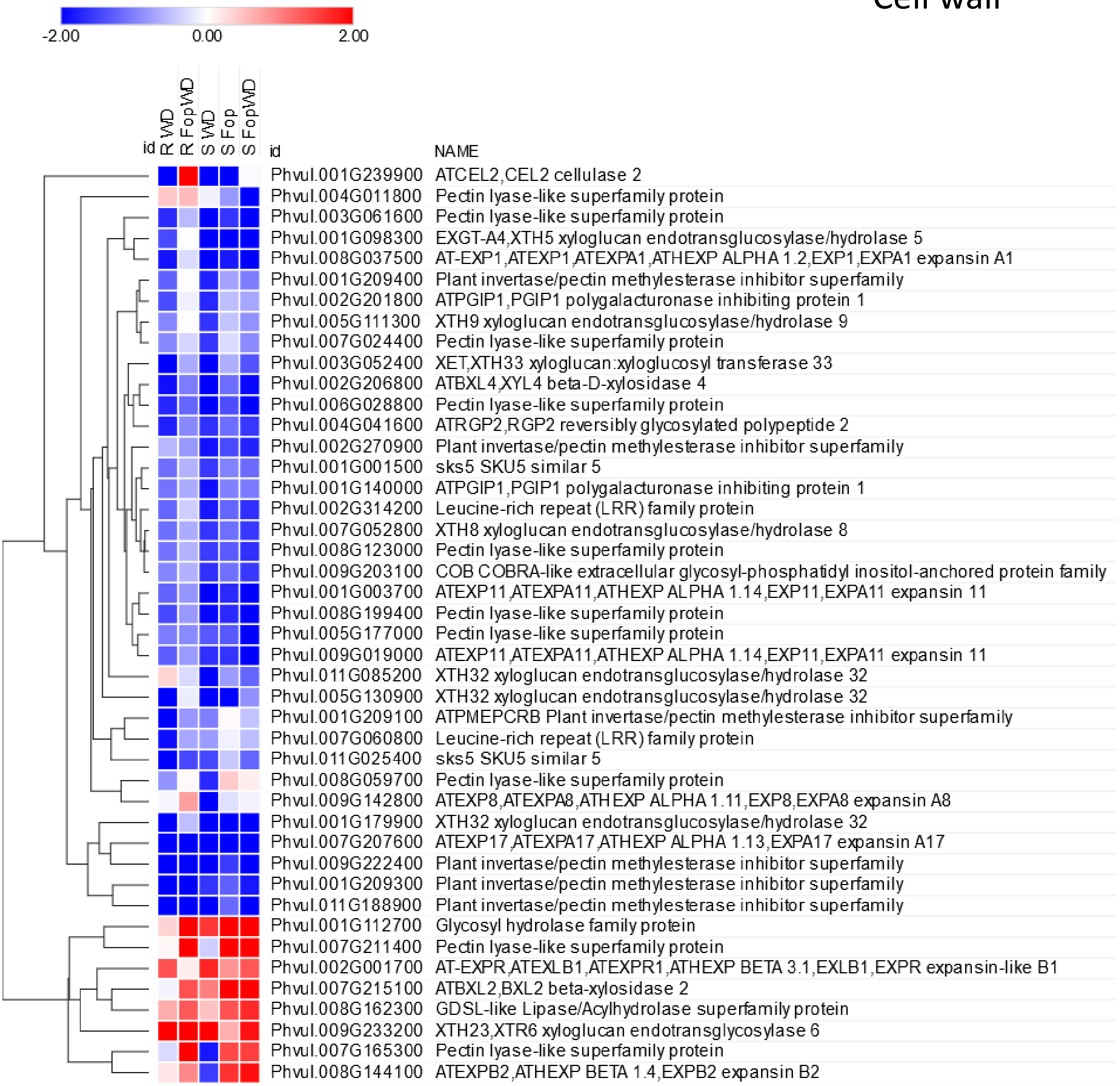
-2.00
0.00
2.00
id
R WD
R FopWD
S WD
S Fop
S FopWD
id
NAME
Phvul.001G239900 ATCEL2,CEL2 cellulase 2
Phvul.004G011800 Pectin lyase-like superfamily protein
Phvul.003G061600 Pectin lyase-like superfamily protein
Phvul.001G098300 EXGT-A4,XTH5 xyloglucan endotransglucosylase/hydrolase 5
Phvul.008G037500 AT-EXP1,ATEXP1,ATEXPA1,ATHEXP ALPHA 1.2,EXP1,EXPA1 expansin A1
Phvul.001G209400 Plant invertase/pectin methylesterase inhibitor superfamily
Phvul.002G201800 ATPGIP1,PGIP1 polygalacturonase inhibiting protein 1
Phvul.005G111300 XTH9 xyloglucan endotransglucosylase/hydrolase 9
Phvul.007G024400 Pectin lyase-like superfamily protein
Phvul.003G052400 XET,XTH33 xyloglucan:xyloglucosyl transferase 33
Phvul.002G206800 ATBXL4,XYL4 beta-D-xylosidase 4
Phvul.006G028800 Pectin lyase-like superfamily protein
Phvul.004G041600 ATRGP2,RGP2 reversibly glycosylated polypeptide 2
Phvul.002G270900 Plant invertase/pectin methylesterase inhibitor superfamily
Phvul.001G001500 sks5 SKU5 similar 5
Phvul.001G140000 ATPGIP1,PGIP1 polygalacturonase inhibiting protein 1
Phvul.002G314200 Leucine-rich repeat (LRR) family protein
Phvul.007G052800 XTH8 xyloglucan endotransglucosylase/hydrolase 8
Phvul.008G123000 Pectin lyase-like superfamily protein
Phvul.009G203100 COB COBRA-like extracellular glycosyl-phosphatidyl inositol-anchored protein family
Phvul.001G003700 ATEXP11,ATEXPA11,ATHEXP ALPHA 1.14,EXP11,EXPA11 expansin 11
Phvul.008G199400 Pectin lyase-like superfamily protein
Phvul.005G177000 Pectin lyase-like superfamily protein
Phvul.009G019000 ATEXP11,ATEXPA11,ATHEXP ALPHA 1.14,EXP11,EXPA11 expansin 11
Phvul.011G085200 XTH32 xyloglucan endotransglucosylase/hydrolase 32
Phvul.005G130900 XTH32 xyloglucan endotransglucosylase/hydrolase 32
Phvul.001G209100 ATPMEPCRB Plant invertase/pectin methylesterase inhibitor superfamily
Phvul.007G060800 Leucine-rich repeat (LRR) family protein
Phvul.011G025400 sks5 SKU5 similar 5
Phvul.008G059700 Pectin lyase-like superfamily protein
Phvul.009G142800 ATEXP8,ATEXPA8,ATHEXP ALPHA 1.11,EXP8,EXPA8 expansin A8
Phvul.001G179900 XTH32 xyloglucan endotransglucosylase/hydrolase 32
Phvul.007G207600 ATEXP17,ATEXPA17,ATHEXP ALPHA 1.13,EXPA17 expansin A17
Phvul.009G222400 Plant invertase/pectin methylesterase inhibitor superfamily
Phvul.001G209300 Plant invertase/pectin methylesterase inhibitor superfamily
Phvul.011G188900 Plant invertase/pectin methylesterase inhibitor superfamily
Phvul.001G112700 Glycosyl hydrolase family protein
Phvul.007G211400 Pectin lyase-like superfamily protein
Phvul.002G001700 AT-EXPR,ATEXLB1,ATEXPR1,ATHEXP BETA 3.1,EXLB1,EXPR expansin-like B1
Phvul.007G215100 ATBXL2,BXL2 beta-xylosidase 2
Phvul.008G162300 GDSL-like Lipase/Acylhydrolase superfamily protein
Phvul.009G233200 XTH23,XTR6 xyloglucan endotransglycosylase 6
Phvul.007G165300 Pectin lyase-like superfamily protein
Phvul.008G144100 ATEXPB2,ATHEXP BETA 1.4,EXPB2 expansin B2

"Cell"

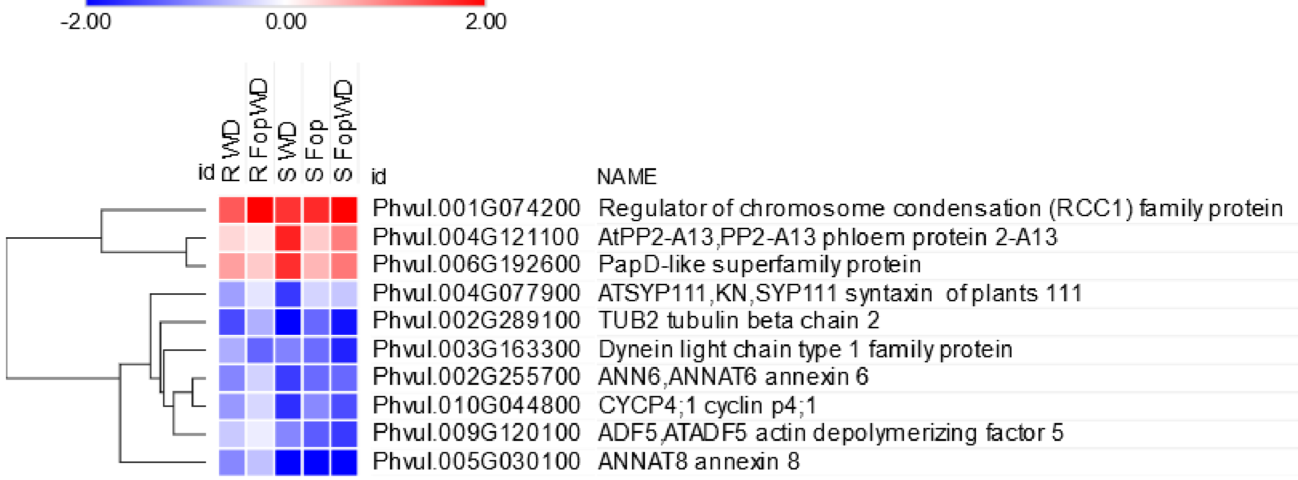

"Development"

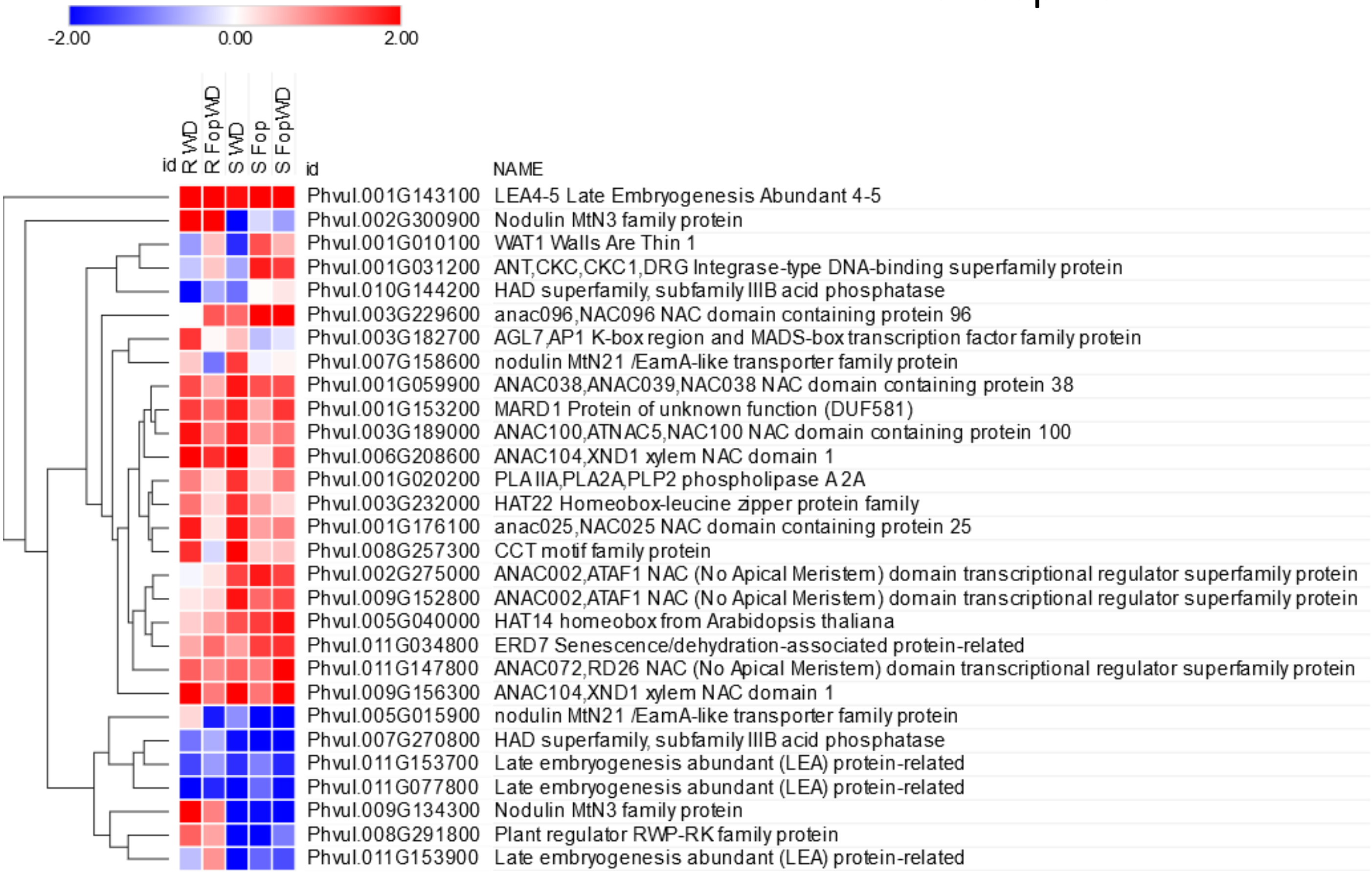

"PS"

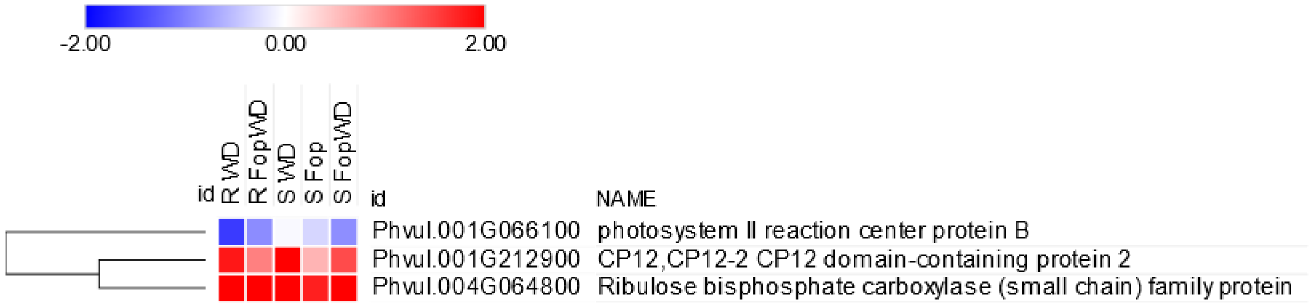

"Minor CHO metabolism"

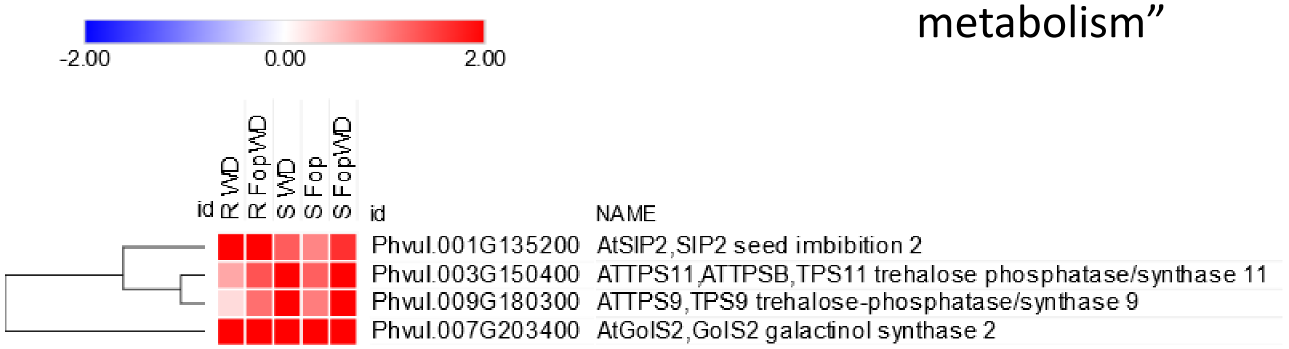

"Major CHO metabolism"

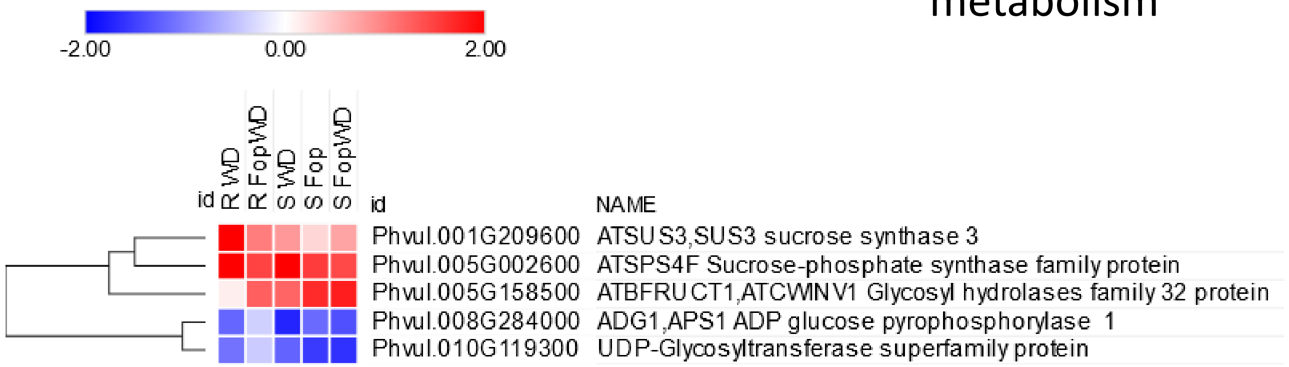

“Miscellaneous”

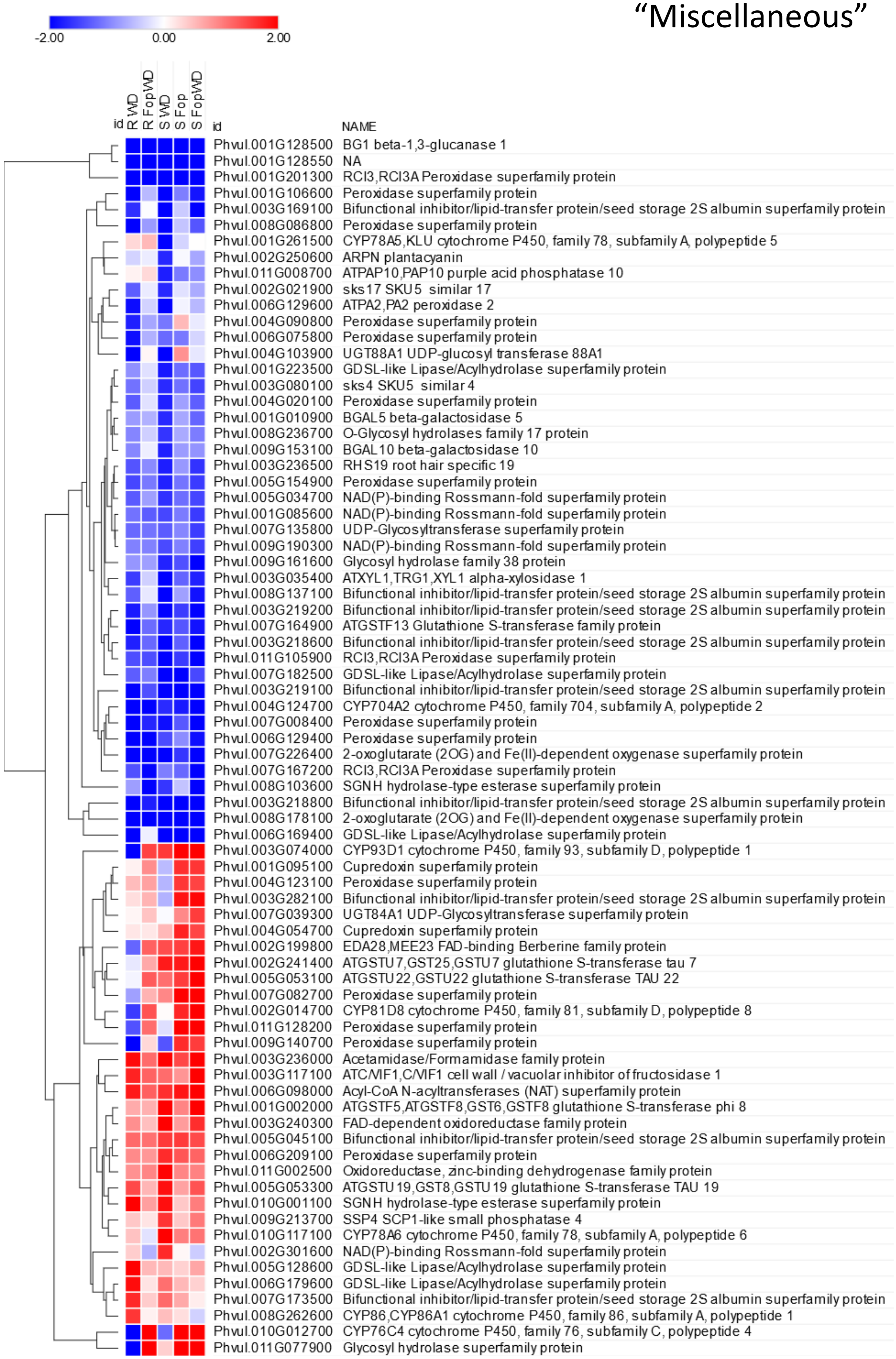

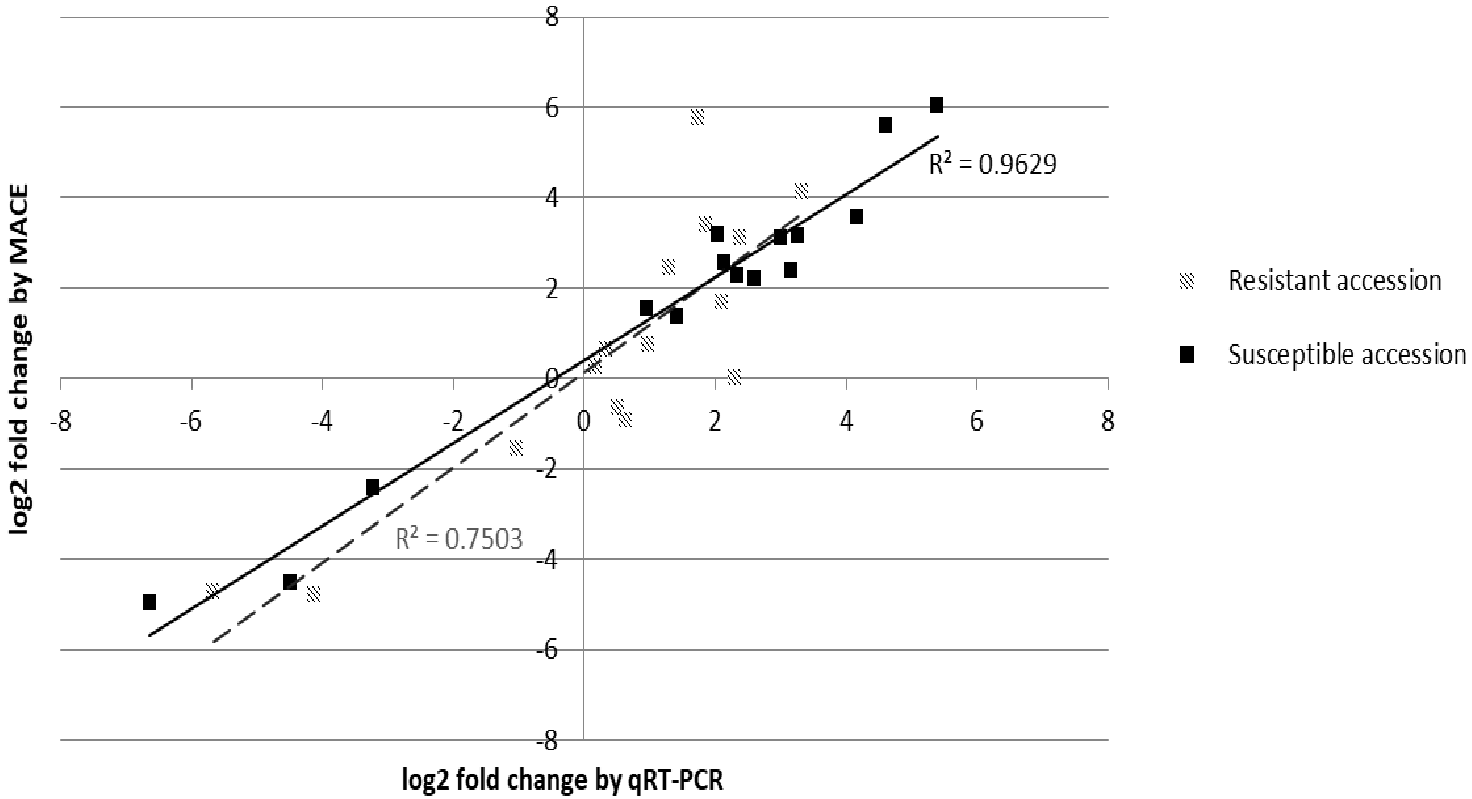

Figure S6: Comparison of relative expression levels of five genes, using $\log_2$ fold change of MACE and qRT-PCR, for the common bean resistant (R-645) (in striped grey) and susceptible (S-1955) (in solid black) accessions, under water deficit, after inoculation with *Fop* and under both stresses combined. Linear regressions for both accessions are shown.

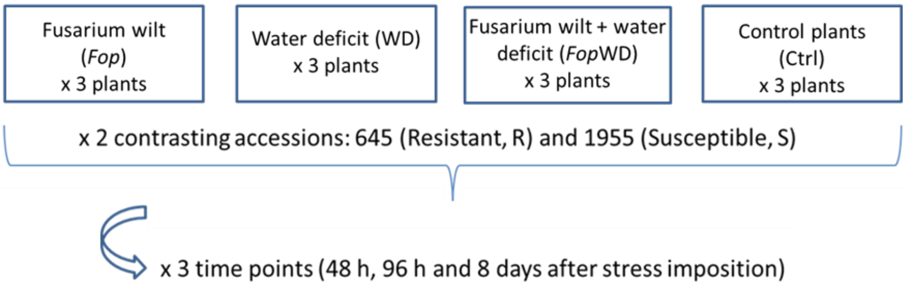

Figure S7: Schematic representation of the biotic-abiotic experimental design. Accessions R-645 (resistant) and S-1955 (susceptible) were evaluated for fusarium wilt, water deficit and the combined stresses (fusarium wilt plus water deficit). Roots from all the plants were collected at 48 h, 96 h and 8 days after stress imposition (*Fusarium oxysporum* f. sp. *phaseoli* inoculation and/or water deficit) for molecular analysis.
