# Supplementary material for: Shared and tailored common bean transcriptomic responses to combined fusarium wilt and water deficit
Source: Hortic Res. 2021 Jul 1;8:149. doi: 10.1038/s41438-021-00583-2 (PMC8245569; doi:10.1038/s41438-021-00583-2)
Supplement: Supplementary file 2 — Supplementary Tables_S1-S5 [file 41438_2021_583_MOESM2_ESM.pdf]

Table S1: Analysis of variance components using REML framework in Genstat (19th ed) and mean values of the leaf relative water content (RWC) and eleven photosynthesis-related traits measured in two Portuguese common bean accessions (one resistant, R-645, and one susceptible, S-1955) under control conditions (no stress applied), after inoculation with *Fusarium oxysporum* f. sp. *phaseoli* (*Fop*), water deficit imposition (WD) and the combination of both stresses (*Fop*WD). The evaluations were performed at three time-points: 48 h, 96 h and 8 days after stress imposition. The interaction between accession, treatment and time-point was analyzed. Post hoc Tukey's test[1] for multiple comparisons of means was performed at a significance level of 95% when the mean value of a trait was different between treatments or time-points. C*a* = chlorophyll *a* concentration, C*b* = chlorophyll *b* concentration, C*cx* = total carotenoids concentration, A = net CO_2 assimilation rate, E = transpiration rate, gs = stomatal conductance.

		RWC		C*a*		C*b*		C*cx*		C*a*+C*b*		Ca/Cb		(Ca+Cb)/Ccx		A		E		gs		A/E		A/gs	
**Effect**		*F pr*	Mean	*F pr*	Mean	*F pr*	Mean	*F pr*	Mean	*F pr*	Mean	*F pr*	Mean	*F pr*	Mean	*F pr*	Mean	*F pr*	Mean	*F pr*	Mean	*F pr*	Mean	*F pr*	Mean
**Accession**	**R-645 (Resistant)**	0.209	80.3	0.005	12.10 b	0.019	5.60 b	0.007	2.95 b	0.006	17.70 b	0.453	2.25	0.480	6.04	<0.001	3.432 b	0.294	2.389	0.118	0.139	<0.001	1.301 b	<0.001	27.117 b
	**S-1955 (Susceptible)**		78.8		10.02 a		4.62 a		2.42 a		14.64 a		2.22		6.22		1.165 a		2.462		0.227		0.359 a		6.767 a
**Treatment**	**Control**	0.543	80.50	<0.001	10.68 ab	<0.001	4.415 a	0.002	2.637 ab	<0.001	15.10 ab	<0.001	2.462 b	<0.001	5.751 a	<0.001	2.526 bc	<0.001	3.074 b	<0.001	0.3271 b	0.039	0.8646 ab	0.716	15.90
	Fop		79.86		11.93 b		6.002 b		2.833 ab		17.93 bc		2.032 a		6.611 b		1.956 ab		1.950 a		0.1046 a		0.8376 ab		19.69
	**WD**		77.60		9.15 a		3.921 a		2.281 a		13.07 a		2.395 b		5.743 a		3.883 c		3.157 b		0.2389 b		1.1505 b		18.52
	***Fop*WD**		80.07		12.47 b		6.102 b		3.004 b		18.57 c		2.059 a		6.418 b		0.828 a		1.522 a		0.0608 a		0.4669 a		13.66
**Time-point**	**T1 - 48 h**	<0.001	80.41 b	<0.001	11.98 b	<0.001	6.214 b	<0.001	2.971 b	<0.001	18.19 b	<0.001	2.002 a	0.026	6.098 ab	0.001	1.784 a	<0.001	1.643 a	<0.001	0.0863 a	0.383	0.7956	0.253	21.47
	**T2 - 96 h**		74.28 a		14.14 c		5.980 b		3.425 c		20.12 b		2.412 b		5.859 a		3.586 b		3.636 b		0.3601 b		0.9754		13.34
	**T3 - 8 days**		83.84 b		7.06 a		3.135 a		1.671 a		10.20 a		2.298 b		6.434 b		1.526 a		1.998 a		0.1020 a		0.7186		16.02
**Interactions**	**Accession x Treatment**	0.218		0.078		0.283		0.129		0.102		0.416		0.674		0.372		0.940		0.014		0.211		0.260	
	**Accession x Time-point**	0.059		0.380		0.953		0.128		0.571		0.077		0.010		0.020		0.095		0.024		0.344		0.939	
	**Treatment x Time-point**	0.225		<0.001		<0.001		<0.001		<0.001		0.931		0.231		0.677		0.082		0.006		0.539		0.576	

[1] Different letters in front of the mean value of a trait indicate significant differences.

		RWC		*Ca*		*Cb*		*Ccx*		*Ca+Cb*		Ca/Cb		(Ca+Cb)/Ccx		A		E		gs		A/E		A/gs	
**Effect**		*F pr*	Mean	*F pr*	Mean	*F pr*	Mean	*F pr*	Mean	*F pr*	Mean	*F pr*	Mean	*F pr*	Mean	*F pr*	Mean	*F pr*	Mean	*F pr*	Mean	*F pr*	Mean	*F pr*	Mean
**Accession**	**R-645 (Resistant)**	0.209	80.3	0.005	12.10 b	0.019	5.60 b	0.007	2.95 b	0.006	17.70 b	0.453	2.25	0.480	6.04	<0.001	3.432 b	0.294	2.389	0.118	0.139	<0.001	1.301 b	<0.001	27.117 b
	**R-1955 (Susceptible)**		78.8		10.02 a		4.62 a		2.42 a		14.64 a		2.22		6.22		1.165 a		2.462		0.227		0.359 a		6.767 a
**R-645 (Resistant)**																									
**Treatment**	**Control**	0.150	84.46	0.006	11.95 ab	<0.001	5.194 ab	0.018	2.914 ab	0.002	17.14 a	0.011	2.338 ab	0.197	5.920	0.002	3.884 b	<0.001	3.175 b	<0.001	0.2089 b	0.029	1.318 ab	0.168	23.64
	Fop		79.46		11.97 ab		6.102 bc		2.873 ab		18.08 ab		2.014 a		6.409		2.648 ab		1.984 a		0.1067 a		1.283 ab		34.50
	**WD**		78.08		10.67 a		4.634 a		2.649 a		15.31 a		2.457 b		5.730		5.002 b		3.050 b		0.2230 b		1.611 b		28.60
	***Fop*WD**		80.04 a		14.27 b		6.814 c		3.454 b		21.08 b		2.098 ab		6.327		1.047 a		1.838 a		0.0928 a		0.575 a		16.61
**Time-point**	**T1 - 48 h**	<0.001	82.97 b	<0.001	12.64 b	<0.001	6.683 b	<0.001	3.085 b	<0.001	19.32 b	<0.001	1.922 a	0.558	6.281	<0.001	1.813 a	<0.001	1.671 a	<0.001	0.0871 a	0.333	1.070	0.145	33.87
	**T2 - 96 h**		73.58 a		15.46 c		6.705 b		3.734 c		22.16 b		2.345 b		5.952		5.147 b		3.436 c		0.2589 b		1.445		20.84
	**T3 - 8 days**		84.98 b		8.55 a		3.669 a		2.099 a		12.22 a		2.414 b		6.057		2.476 a		2.427 b		0.1276 a		1.076		22.82
**Interactions**	**Treatment x Time-point**	0.485		<0.001		0.006		0.001		<0.001		0.237		0.763		0.278		<0.001		0.013		0.347		0.175	
**S-1955 (Susceptible)**																									
**Treatment**	**Control**	0.533	77.52	<0.001	9.62 b	<0.001	3.857 a	<0.001	2.397 b	<0.001	13.48 ab	<0.001	2.522 b	<0.001	5.661 a	<0.001	0.889 a	<0.001	2.848 c	<0.001	0.3946 b	0.010	0.2420 a	0.552	3.944
	Fop		80.43		12.21 b		5.980 b		2.847 b		18.19 c		2.023 a		7.043 c		0.756 a		2.113 b		0.1381 a		0.2343 a		4.105
	**WD**		77.91		6.79 a		2.992 a		1.694 a		9.78 a		2.273 ab		5.845 a		2.214 b		2.999 c		0.2159 a		0.6784 b		10.779
	***Fop*WD**		79.98		11.43 b		5.682 b		2.718 b		17.11 bc		2.037 a		6.561 b		0.217 a		1.477 a		0.0762 a		0.1368 a		5.313
**Time-point**	**T1 - 48 h**	0.034	78.27 ab	<0.001	11.26 b	<0.001	5.778 b	<0.001	2.826 b	<0.001	17.04 b	<0.001	2.042 a	<0.001	6.024 a	0.010	1.440 b	<0.001	1.513 a	<0.001	0.0894 a	0.063	0.4922	0.142	11.615
	**T2 - 96 h**		76.46 a		13.32 b		5.525 b		3.240 b		18.84 b		2.458 b		5.771 a		1.277 b		3.822 b		0.4139 b		0.3177		3.644
	**T3 - 8 days**		82.14 b		5.45 a		2.580 a		1.177 a		8.04 a		2.141 a		7.038 b		0.340 a		1.743 a		0.1152 a		0.1587		2.846
**Interactions**	**Treatment x Time-point**	0.189		0.006		0.002		0.005		0.005		0.003		<0.001		<0.001		<0.001		<0.001		0.003		0.451	

[1] Different letters in front of the mean value of a trait indicate significant differences.

Table S2: Tukey's test[1] for multiple comparisons of means performed at a significance level of 95% for each time point (T1 = 48 h, T2 = 96 h and T3 = 8 days after stress imposition). Two Portuguese common bean accessions (one resistant, R-645, and one susceptible, S-1955) were tested together under four different treatments: control conditions (Ctrl, no stress applied), after inoculation with *Fusarium oxysporum* f. sp. *phaseoli* (*Fop*), water deficit imposition (WD), and the combination of both stresses (*Fop*WD). Leaf relative water content (RWC) and eleven photosynthesis-related traits were evaluated. C*a* = chlorophyll *a* concentration, C*b* = chlorophyll *b* concentration, C*cx* = total carotenoids concentration, A = net CO_2 assimilation rate, E = transpiration rate, gs = stomatal conductance.
For each column (time point), different letters in front of the mean value of a trait indicate significant differences between samples.

**Sample / time point**	**RWC**			**A**			**E**			**gs**			**A/E**			**A/gs**		
	T1	T2	T3	T1	T2	T3	T1	T2	T3	T1	T2	T3	T1	T2	T3	T1	T2	T3
R Ctrl	88.21 a	80.13 a	83.99 a	3.882 ab	4.654 abc	3.635 b	3.198 cd	3.812 bc	2.164 a	0.19083 c	0.3025 a	0.1130 a	1.2218 a	1.2455 ab	1.7674 c	21.04 a	16.73 ab	39.09 b
R *Fop*	82.43 a	70.30 a	85.66 a	0.526 a	5.732 bc	2.278 ab	0.487 a	2.912 ab	2.055 a	0.01002 a	0.1820 a	0.0930 a	1.1220 a	1.9494 b	1.1238 abc	52.48 a	33.30 b	25.15 ab
R WD	77.79 a	70.59 a	86.01 a	2.495 ab	8.202 c	3.831 b	2.117 bc	4.307 cd	2.875 a	0.09583 b	0.4075 a	0.1725 a	1.3095 a	1.8990 b	1.3748 bc	33.13 a	20.87 ab	25.71 ab
R *Fop*WD	83.25 a	72.25 a	84.26 a	0.287 a	2.519 ab	0.514 a	0.514 a	2.364 a	2.192 a	0.01502 ab	0.1233 a	0.1000 a	0.6554 a	0.9650 ab	0.2605 ab	28.64 a	18.38 ab	5.71 a
S Ctrl	72.82 a	76.86 a	83.00 a	0.139 a	2.279 ab	0.001 a	0.513 a	5.295 d	2.682 a	0.01252 a	0.9417 b	0.1750 a	0.2401 a	0.4332 a	0.0006 a	9.17 a	2.61 a	0.01 a
S *Fop*	81.93 a	76.46 a	82.65 a	0.000 a	2.087 ab	0.078 a	1.117 ab	3.425 abc	1.664 a	0.04000 ab	0.2436 a	0.0694 a	0.0000 a	0.6074 ab	0.1492 ab	0.00 a	8.62 ab	7.83 ab
S WD	74.13 a	77.32 a	82.59	5.249 b	0.003 a	1.325	3.896 d	3.727 bc	1.325	0.27583 d	0.3168 a	0.0575	1.3535 a	0.0007 a	0.6848	19.42 a	0.01 a	13.95

			a			ab			a			a			abc			ab
S *Fop*WD	84.21 a	73.97 a	80.29 a	0.157 a	0.912 ab	0.001 a	0.530 a	2.853 ab	1.044 a	0.01250 a	0.1644 a	0.0384 a	0.2917 a	0.2860 a	0.0006 a	15.75 a	4.41 a	0.01 a

Continuation of Suppl Table 2

**Sample / time point**	**C*a***			**C*b***			**C*cx***			**C*a*+C*b***			**C*a*/C*b***			**(C*a*+C*b*)/C*cx***		
	T1	T2	T3	T1	T2	T3	T1	T2	T3	T1	T2	T3	T1	T2	T3	T1	T2	T3
R Ctrl	12.01 ab	16.61bc	7.141 abc	6.084 ab	6.449 abc	2.768 ab	2.947 a	4.074 bc	1.728 ab	18.09 ab	23.06 bc	9.909 abc	1.982 abc	2.565 b	2.601 ab	6.135 ab	5.682 a	5.730 a
R *Fop*	13.46 ab	13.82 abc	8.642 bc	7.566 b	6.849 bc	3.890 bc	3.200 a	3.326 abc	2.094 ab	21.03 ab	20.67 abc	12.532 bc	1.773 ab	2.029 a	2.241 ab	6.599 b	6.222 b	6.407 ab
R WD	11.97 ab	10.47 ab	9.564 c	6.330 ab	4.063 ab	3.484 abc	2.997 a	2.523 ab	2.137 ab	18.30 ab	14.53 ab	13.049 c	1.960 abc	2.628 b	2.797 b	6.063 ab	5.730 ab	5.323 a
R *Fop*WD	13.05 ab	20.85 c	8.852 bc	6.562 ab	9.180 c	4.534 c	3.220 a	5.019 c	2.137 ab	19.61 ab	30.03 c	13.386 c	2.068 ab	2.292 ab	2.017 ab	6.069 ab	5.962 ab	6.768 ab
S Ctrl	10.17 ab	13.17 abc	4.936 ab	4.115 a	5.279 ab	2.018 a	2.636 a	3.229 abc	1.160 a	14.29 a	18.45 abc	6.953 a	2.492 c	2.525 b	2.534 ab	5.410 a	5.690 a	5.969 a
S *Fop*	14.78 b	14.67 abc	5.903 abc	8.344 b	5.701 abc	3.440 abc	3.617 a	3.518 abc	1.044 a	23.12 b	20.37 abc	9.343 abc	1.778 ab	2.567 b	1.718 a	6.493 b	5.798 ab	8.966 b
S WD	9.60 a	7.39 a	4.284 a	4.317 a	2.936 a	2.072 a	2.443 a	1.868 a	1.025 a	13.91 a	10.33 a	6.355 a	2.244 bc	2.515 b	2.056 ab	5.697 ab	5.540 a	6.227 ab
S	10.50 ab	17.35 bc	5.280	6.335 ab	7.780 bc	2.391	2.607 a	4.155	1.068 a	16.84 ab	25.13 bc	7.671	1.656 a	2.253 ab	2.208	6.498 b	6.027 ab	7.181

*Fop*WD			ab			ab		bc				ab			ab			ab

[1] For each column (time point), different letters in front of the mean value of a trait indicate significant differences.

Table S3: Tukey's test[1] for multiple comparisons of means performed at a significance level of 95% for each time point (T1 = 48 h, T2 = 96 h and T3 = 8 days after stress imposition). Two Portuguese common bean accessions (one resistant, R-645, and one susceptible S-1955) were tested separately under four different treatments: control conditions (Ctrl, no stress applied), after inoculation with *Fusarium oxysporum* f. sp. *phaseoli* (*Fop*), water deficit imposition (WD), and the combination of both stresses (*Fop*WD). Leaf relative water content (RWC) and eleven photosynthesis-related traits were evaluated. C*a* = chlorophyll *a* concentration, C*b* = chlorophyll *b* concentration, C*cx* = total carotenoids concentration, A = net CO_2 assimilation rate, E = transpiration rate, gs = stomatal conductance. For each column (time point), different letters in front of the mean value of a trait indicate significant differences between samples.

**Sample / time point**	**RWC**			**A**			**E**			**gs**			**A/E**			**A/gs**		
	T1	T2	T3	T1	T2	T3	T1	T2	T3	T1	T2	T3	T1	T2	T3	T1	T2	T3
R Ctrl	88.21 a	80.13 a	83.99 a	3.882 a	4.654 ab	3.635 b	3.198 b	3.812 ab	2.164 a	0.19083 b	0.3025 ab	0.1130 a	1.222 a	1.246 a	1.767 b	21.04 a	16.73 a	39.09 b
R *Fop*	82.43 a	70.30 a	85.66 a	0.526 a	5.732 ab	2.278 ab	0.487 a	2.912 ab	2.055 a	0.01002 a	0.1820 a	0.0930 a	1.123 a	1.949 a	1.124 ab	52.46 a	33.30 a	25.15 ab
R WD	77.79 a	70.59 a	86.01 a	2.495 a	8.202 b	3.831 b	2.117 b	4.307 b	2.875 a	0.09583 ab	0.4075 b	0.1725 a	1.309 a	1.899 a	1.375 ab	33.13 a	20.87 a	25.71 ab
R *Fop*WD	83.25 a	72.25 a	84.26 a	0.288 a	2.519 a	0.515 a	0.514 a	2.364 a	2.192 a	0.01502 a	0.1233 a	0.1000 a	0.656 a	0.965 a	0.261 a	28.65 a	18.38 a	5.72 a

**Sample / time point**	**RWC**			**A**			**E**			**gs**			**A/E**			**A/gs**		
	T1	T2	T3	T1	T2	T3	T1	T2	T3	T1	T2	T3	T1	T2	T3	T1	T2	T3
S Ctrl	72.82 a	76.86 a	83.00 a	0.138 a	2.279 a	0.0002 a	0.513 a	5.295 c	2.682 a	0.01252 a	0.9417 b	0.17498 b	0.2395 a	0.4332 a	0.0001 a	9.20 a	2.612 a	0.004 a
S *Fop*	81.93 a	76.46 a	82.65 a	0.000 a	2.085 a	0.0783 a	1.117 a	3.425 ab	1.664 a	0.04000 a	0.2435 a	0.06944 ab	0.0000 a	0.6069 a	0.1492 a	0.00 a	8.616 a	7.833 a
S WD	74.13 a	77.33 a	82.59 a	5.249 b	0.001 a	1.3243 a	3.896 b	3.727 b	1.325 a	0.27583 b	0.3168 a	0.05751 a	1.3535 b	0.0002 a	0.6844 a	19.42 a	0.003 a	13.942 a
S *Fop*WD	84.21 a	73.98 a	80.29 a	0.158 a	0.912 a	0.0002 a	0.530 a	2.853 a	1.044 a	0.01250 a	0.1644 a	0.03834 a	0.2917 a	0.2860 a	0.0001 a	15.75 a	4.414 a	0.004 a

**Sample / time point**	**C*a***			**C*b***			**C*cx***			**C*a*+C*b***			**C*a*/C*b***			**(C*a*+C*b*)/C*cx***		
	T1	T2	T3	T1	T2	T3	T1	T2	T3	T1	T2	T3	T1	T2	T3	T1	T2	T3
R Ctrl	12.01 a	16.61 ab	7.141 a	6.084 a	6.449 ab	2.768 a	2.947 a	4.074 ab	1.728 a	18.09 a	23.06 ab	9.91 a	1.982 a	2.565 b	2.601 a	6.135 a	5.682 a	5.730 a
R *Fop*	13.46 a	13.82 a	8.642 a	7.566 a	6.849 ab	3.890 ab	3.200 a	3.326 a	2.094 a	21.03 a	20.67 ab	12.53 a	1.773 a	2.029 a	2.241 a	6.599 a	6.222 a	6.407 a
R WD	11.97 a	10.47 a	9.564 a	6.330 a	4.063 a	3.484 ab	2.997 a	2.523 a	2.437 a	18.30 a	14.53 a	13.05 a	1.960 a	2.628 b	2.797 a	6.063 a	5.730 a	5.323 a
R *Fop*WD	13.05 a	20.85 b	8.852 a	6.562 a	9.180 b	4.534 b	3.220 a	5.019 b	2.137 a	19.61 a	30.03 b	13.39 a	2.068 a	2.292 ab	2.017 a	6.069 a	5.962 a	6.768 a

**Sample / time point**	**C*a***			**C*b***			**C*cx***			**C*a*+C*b***			**C*a*/C*b***			**(C*a*+C*b*)/C*cx***		
	T1	T2	T3	T1	T2	T3	T1	T2	T3	T1	T2	T3	T1	T2	T3	T1	T2	T3
S Ctrl	10.17 ab	13.17 ab	4.934 a	4.115 a	5.279 ab	2.017 a	2.636 a	3.229 ab	1.160 a	14.29 a	18.45 ab	6.951 a	2.492 c	2.525 a	2.534 b	5.410 a	5.690 a	5.969 a
S *Fop*	14.78 b	14.67 ab	5.901 a	8.344 c	5.701 ab	3.440 a	3.617 a	3.518 ab	1.044 a	23.12 b	20.37 ab	9.341 a	1.778 ab	2.567 a	1.718 a	6.493 b	5.798 ab	8.967 b
S WD	9.60 a	7.39 a	4.284 a	4.317 ab	2.936 a	2.072 a	2.443 a	1.867 a	1.025 a	13.91 a	10.33 a	6.355 a	2.244 bc	2.515 a	2.056 ab	5.697 ab	5.540 a	6.227 a
S *Fop*WD	10.50 ab	17.34 b	5.278 a	6.335 bc	7.780 b	2.390 a	2.607 a	4.155 b	1.068 a	16.84 ab	25.12 b	7.669 a	1.656 a	2.253 a	2.208 ab	6.498 b	6.027 b	7.182 a

Table S4 - Functional categories of the DEGs identified for the resistant (R-645) and susceptible (S-1955) common bean accessions, in water deficit conditions (WD), after *Fusarium oxysporum* f. sp. *phaseoli* SP1-race 6 inoculation (Fop), applied as single and combined stresses (FopWD). The functional categorization was made in relation to the control conditions, using MapMan software.

**Functional categories for R WD**	**No. genes**
Not assigned	42
Misc (peroxidades, cytochrome P450, lipases, glucanases, oxidades, transferases)	27
RNA regulation of transcription	16
Protein modification	15
Cell wall	11
Hormone metabolism	11
Stress (Biotic/Abiotic)	10
Development	8
Signaling	7
Secondary metabolism	6
Transport	5
Minor CHO metabolism	3
Lipid metabolism	2
Metal handling	2
Major CHO metabolism	2
Nucleotide metabolism	2
Glycolysis	2
PS light reaction	1
Co-factor and vitamin metabolism	1
TCA	1
Mitochondrial electron transport	1
**Functional categories for R *Fop*WD**	**No. genes**
Not assigned	16
Stress (Biotic/Abiotic)	11
Misc (Cytochrome P450, peroxidases, transferases, hydrolases, glucosidases, oxidases, nitrilases)	10
Secondary metabolism	6
Hormone metabolism	5
Cell Wall	5
Transport	5
RNA regulation of transcription	3
Protein modification	3
Minor CHO metabolism	3
Signaling	2
PS light reaction	1
DNA synthesis	1
OPP non-reductive	1
**Functional categories for S WD**	**No. genes**
Not assigned	150
RNA (regulation of transcription)	57
Misc (peroxidases, transferases, proteases inhibitor, oxidases, glucosidases etc)	54

Cell Wall	39
Signaling	36
Hormone metabolism	33
Stress (Biotic/Abiotic)	22
Transport	21
Protein (modification)	20
Secondary metabolism	20
Development	18
Lipid metabolism	12
Metal handling	7
Glycolysis	7
Cell	7
Amino acid metabolism	6
Redox	5
Nucleotide metabolism	4
DNA	3
Fermentation	3
TCA	2
Major CHO metabolism	2
Minor CHO metabolism	2
C1 metabolism	1
Polyamine metabolism	1
PS	1
Tetrapyrrole synthesis	1
**Functional categories for S *Fop***	**No. genes**
Not assigned	55
Misc (peroxidases, plastocyanin-like, transferases, lipases, glucosidades)	18
RNA regulation of transcription	18
Stress (Biotic/Abiotic)	12
Hormone metabolism	11
Transport	11
Protein modification	8
Secondary metabolism	7
Signaling	7
Development	6
Cell Wall	5
Lipid metabolism	4
Minor CHO metabolism	3
Major CHO metabolism	2
Metal handling	1
Fermentation	1
**Functional categories for S *Fop*WD**	**No. genes**
Not assigned	84
Misc (cytochrome P450, protease inhibitor, lipases, glucosidases, hydrolases, nitrilases, oxidases)	43
RNA regulation of transcription	27
Hormone metabolism	24
Cell wall	24
Stress (Biotic and Abiotic)	24
Secondary metabolism	22

Signaling	18
Protein modification	17
Transport	16
Development	11
Lipid metabolism	7
Metal Handling	5
Cell division, organization	5
Minor CHO metabolism	3
Amino acids metabolism	2
Major CHO metabolism	2
Redox	2
Fermentation	2
OPP.non-reductive	1

Table S5: List of selected genes and primers used in quantitative real-time PCR**.**

Gene ID	Annotation	Primer forward sequence (5'-3')	Primer reverse sequence(5'-3')
Phvul.002G104100 (reference gene)	RNA-binding (RRM/RBD/RNP motifs) family protein	CGAAGGAAGCGTGCAAGAAC	CACGCTCTCCACGATATGCA
Phvul.003G082700 (reference gene)	Indole-3-butyric acid response 1 (IBR1, SDRA)	CTGAGATGGCCCCAGACAC	TCTGTTGTACCAAGCCTTCCA
Phvul.001G145600 (target gene)	Disease resistance-responsive (dirigent-like protein) family protein	TCATGACCATGGCCTTCACC	ATGGCCATTTCCCTCACAGG
Phvul.001G201300 (target gene)	Peroxidase superfamily protein (RCI3A)	AGTATCCATCAGTGGTGAAGC	GACCAACTCCAAATGTGTGTGC
Phvul.003G096700 (target gene)	Drought-induced 21	TTCTCTCTCGCAACTCTGTGTC	ATTACCCAGGCCAAGTCTCAC
Phvul.007G203400 (target gene)	Galactinol synthase 2 (GolS2)	CCATGTTATGGCGTCACCCT	CCTCTTTCCCACTGTACCTCC
Phvul.007G231800 (target gene)	Myb domain protein 79 (MYB79)	GTTGCATGCTAGATGGGGAAAC	CTGCATCAGAGGGACTTTTTGC
